# A Combined Experimental and Computational Study of Novel Benzotriazinone Carboxamides as Alpha-Glucosidase Inhibitors

**DOI:** 10.3390/molecules28186623

**Published:** 2023-09-14

**Authors:** Zunera Khalid, Syed Salman Shafqat, Hafiz Adnan Ahmad, Munawar Ali Munawar, Sadaf Mutahir, Safaa M. Elkholi, Syed Rizwan Shafqat, Rahila Huma, Abdullah Mohammed Asiri

**Affiliations:** 1 Department of Chemistry, Kinnaird College for Women, Lahore 54000, Pakistan; zunairah.umar@gmail.com (Z.K.); rahila.huma@kinnaird.edu.pk (R.H.); 2 School of Chemistry, University of the Punjab, Lahore 54590, Pakistan; adnan.ahmad.pu@hotmail.com; 3 Department of Chemistry, Division of Science and Technology, University of Education, Lahore 54770, Pakistan; 4 Department of Basic and Applied Chemistry, Faculty of Science and Technology, University of Central Punjab, Lahore 54000, Pakistan; 5 School of Chemistry and Chemical Engineering, Linyi University, Linyi 276000, China; sadafmutahir@hotmail.com; 6 Department of Rehabilitation Sciences, College of Health and Rehabilitation Sciences, Princess Nourah bint Abdulrahman University, P.O. Box 84428, Riyadh 11671, Saudi Arabia; smelkholi@pnu.edu.sa; 7 Department of Chemistry, University of Sialkot, Sialkot 51310, Pakistan; syedrizwanshafqat@gmail.com; 8 Chemistry Department, Faculty of Science, King Abdulaziz University, Jeddah 64274, Saudi Arabia; aasiri2@kau.edu.sa

**Keywords:** diabetes mellitus, benzotriazinone, alpha-glucosidase inhibitor, molecular docking

## Abstract

Diabetes is a chronic metabolic disorder of the endocrine system characterized by persistent hyperglycemia appears due to the deficiency or ineffective use of insulin. The glucose level of diabetic patients increases after every meal and medically recommended drugs are used to control hyperglycemia. Alpha-glucosidase inhibitors are used as antidiabetic medicine to delay the hydrolysis of complex carbohydrates. Acarbose, miglitol, and voglibose are commercial drugs but patients suffer side effects of flatulence, bloating, diarrhea, and loss of hunger. To explore a new antidiabetic drug, a series of benzotriazinone carboxamides was synthesized and their alpha-glucosidase inhibition potentials were measured using *in vitro* experiments. The compounds **14k** and **14l** were found to be strong inhibitors compared to the standard drug acarbose with IC_50_ values of 27.13 ± 0.12 and 32.14 ± 0.11 μM, respectively. *In silico* study of **14k** and **14l** was carried out using molecular docking to identify the type of interactions developed between these compounds and enzyme sites. Both potent compounds **14k** and **14l** exhibited effective docking scores by making their interactions with selected amino acid residues. Chemical hardness and orbital energy gap values were investigated using DFT studies and results depicted affinity of **14k** and **14l** towards biological molecules. All computational findings were found to be in good agreement with *in vitro* results.

## 1. Introduction

Diabetes is a multifactorial metabolic chronic disease characterized by hyperglycemia. It is prevalent in all continents and affects an individual’s life with no distinction of age and gender [1,2]. Previously, it was known as an adult-onset disease, but in the last two decades, many children and teenagers have been reported as victims of this ailment [3]. According to a World Health Organization report, nearly 250 million people suffer from this disease and this population will increase to 366 million by 2030 [4]. This continuous increase in diabetic patients is an alarming situation for the world [5,6]. Early indications of diabetes are frequent urination, excessive thirst, shortness of breath, and nausea but continued uncontrolled glucose levels cause complications such as vascular syndrome, retinopathy, cardiomyopathy, neuropathy, and nephropathy [3,7,8]. Alpha-glucosidase inhibitor drugs are used to suppress the digestion and absorption of carbohydrates [9]. Acarbose (Glucobay, Precose, Prandase), voglibose (Basen), and miglitol (Glyset) are famous alpha-glucosidase inhibitor drugs, but they induce side effects such as bloating, flatulence, diarrhea, and loss of appetite. The synthetic route of marketed drugs is a long process and chemists are working to discover new class of alpha-glucosidase inhibitors [10].

**Figure 1:**
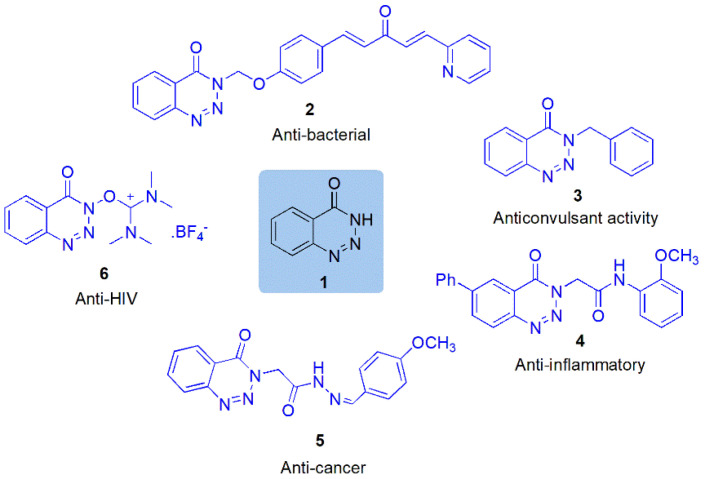
Reported benzotriazinone derivatives as bioactive compounds.

Heterocyclic compounds are widely used as pharmaceutical drugs and 1,2,3-benzotriazin-4(3*H*)-one is one of the emerging nuclei for use as a bioactive scaffold. Previously, several analogues of benzotriazinone **1** have been reported as antibacterial **2** [11], anticonvulsant **3** [12], anti-inflammatory **4** [13], anticancer **5** [14], and anti-HIV **6** [15] (Figure 1). Moreover, different benzotriazinone derivatives have been studied for their inhibition potency against hydrolase aminopeptidase [16], acetylcholinesterase [17], chorismate mutase [18], HepG2 [14], 4-hydroxyphenylpyruvate dioxygenase [19], and matrix metalloprotease [5]. Carboxamide is a popular pharmaceutical due to its diverse spectrum of activities. Carbamazepine [20], oxcarbazepine [21], rufinamide [22], and meloxicam [23] are recognized carboxamide drugs. Nateglinide and anagliptin are commercial antidiabetic pills [24,25]. In the recent literature, compounds like acridine-9-carboxamide and 6-amino-pyrazolo[1,5-a]pyrimidine-3-carboxamide have been explored as effective alpha-glucosidase inhibitors [9,26].

**Figure 2:**
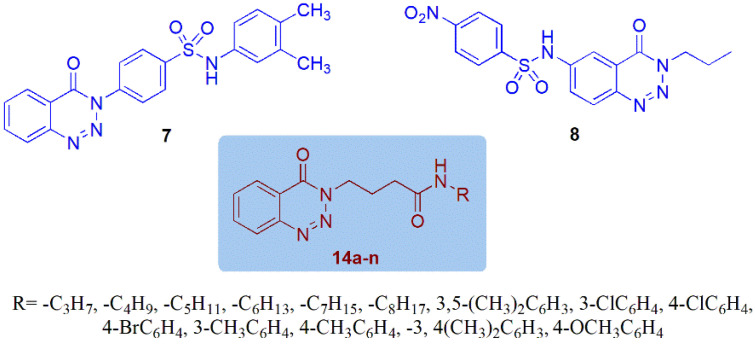
Design of present work.

Recent trends in medicinal research include molecular hybridization of two or more enzyme inhibitors to synthesize new, effective drugs [27,28]. This methodology has been used to prepare influential hybrids of benzotriazinone with other bioactive moieties. Our group is working on the discovery of new alpha-glucosidase inhibitors with better therapeutic activity. In this regard, two series of sulfonamides were synthesized in previous work and their inhibition potential was measured [5,29]. Among previously studied analogues, compounds **7** and **8** (Figure 2) were found to be most potent inhibitors compared to the commercial drug acarbose. In further continuation of work to develop better antidiabetic drugs, a series of benzotriazinone carboxamide derivatives (**14a**–**14n**) were prepared and evaluated for their enzyme inhibition potential. Molecular docking studies were also utilized to explain *in vitro* studies through ligand binding inside enzyme cavity. Electronic parameters of all newly synthesized hybrids were analyzed using DFT studies to explore structure competence in interaction with biomolecules.

## 2. Result and Discussion

### 2.1. Chemistry

**Scheme 1:**
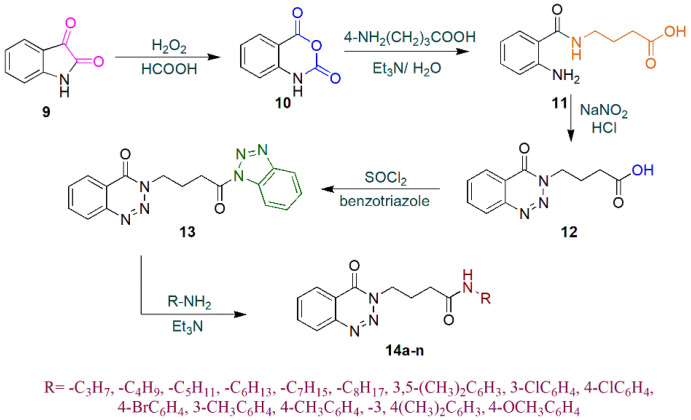
Oxobenzo[1,2,3]triazin-3(4*H*)-yl)-*N*-alkyl/phenyl butanamides.

A series of N-alkyl/phenyl-4-(4-oxobenzo[1,2,3]triazin-3(4*H*)-yl)butanamides (**14a**–**14n**) were synthesized at room temperature by utilizing isatin **9** as an inexpensive starting material. In the first step, hydrogen peroxide and formic acid were applied for the oxidation of **9** to isatoic anhydride **10**. Afterwards, 4-aminobutyric acid was taken in a water: triethylamine mixture and treated with anhydride **10** to afford *N*-(carboxybutyl)anthranilamide **11** that was subsequently diazotized with NaNO_2_/HCl solution to formulate 4-(4-oxobenzo[1,2,3]triazin-3(4*H*)-yl)butanoic acid **12** (Scheme 1). Further, it was reacted with benzotriazole and thionyl chloride to prepare the main precursor **13** and was used with different amines or anilines to afford different carboxamide derivatives **14a**–**14n** (Table 1).

### 2.2. Spectroscopic Analysis

Chemical structures of all compounds were characterized using FT-IR, ^1^H-NMR, ^13^C-NMR, and EIMS spectroscopic techniques. In FT-IR spectrum, isatoic anhydride **10** was confirmed by two carbonyl bands at 1765, 1722 cm^−1^ and these values were found to be consistent with the literature [29]. Further, this compound was used with 4-amino butyric acid to afford **11**, which was subsequently diazotized without purification, and product **12** was obtained. ^1^H-NMR showed one multiplet at 2.25 ppm and two triplets were seen at 2.48 and 4.54 ppm. Four proton signals were observed in the aryl region as two doublets and two triplets. Absence of NH and NH_2_ peaks in ^1^H-NMR spectrum confirmed cyclization of **11** to benzotriazinone **12**. Furthermore, the MS result depicted the highest fragmentation peak at *m*/*z* 187 due to the removal of the carboxylic group. A hydroxyl moiety of acid **12** was replaced by benzotriazole and structure **13** was obtained as the product. In the ^1^HNMR spectrum, three proton signals were observed in the region of 2.55–4.69 ppm and eight protons appeared in the aryl region of 7.48–8.31 ppm. ^13^C-NMR also verified this structure **13** by twelve carbon peaks between 114.37–146.61 ppm and two carbonyl carbon peaks at 155.74 and 171.37 ppm. EIMS spectra confirmed this structure by the molecular ion peak at *m*/*z* 333. Further, this compound was used to prepare a novel series of alkyl and aryl carboxamides **14a**–**14n**. ^1^H-NMR spectra of all carboxamides showed an NH peak in the range of 5.95–8.26 ppm.

**Table 1:** Reaction conditions for the preparation of benzotriazinone carboxamides.

Sr. No.	Codes	Molecular Structures	Time	Temperature (°C)	Yield (%)
1.	**14a**	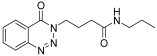	10 min	25	91
2.	**14b**	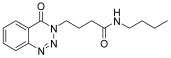	10 min	25	85
3.	**14c**	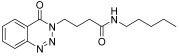	15 min	25	86
4.	**14d**	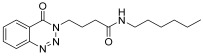	20 min	25	85
5.	**14e**	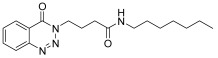	25 min	25	83
6.	**14f**	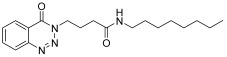	30 min	25	84
7.	**14g**	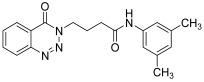	24 h	25	75
8.	**14h**	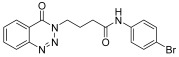	24 h	25	71
9.	**14i**	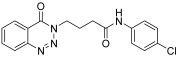	24 h	25	69
10.	**14j**	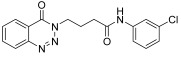	24 h	25	71
11.	**14k**	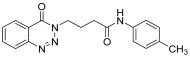	24 h	25	73
12.	**14l**	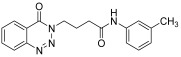	24 h	25	72
13.	**14m**	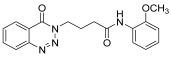	24 h	25	76
14.	**14n**	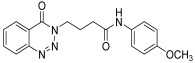	24 h	25	80

### 2.3. Enzyme Inhibition Assay

The alpha-glucosidase inhibitory potential of all synthesized compounds, **14a**–**14n**, were evaluated through their *in vitro* assays and results revealed moderate to excellent activity. The experimental results of all compounds are shown in Table 2. Acarbose was taken as a positive control and the inhibition results of all tested compounds were expressed as percentage inhibition and IC_50_ values. Among all compounds, **14k** and **14l** were found to be potent inhibitors with IC_50_ values of 27.13 ± 0.12 μM and 32.14 ± 0.11 μM in comparison to the standard drug acarbose with an IC_50_ value of 37.38 ± 0.12 μM. The nature of the group and its relative position on the phenyl ring of carboxamides affect inhibition potential. Compound **14k** was observed as more effective than **14l** due to methyl substituent at the *para* position, which enables it to be a better fit in the enzyme cavity. Moreover, compound **14g**, with two methyl substituents, was noticed to be less efficient than **14k**. Compounds **14g**–**14n** showed good inhibition compared to **14a**–**14f**, which demonstrates the importance of aryl groups in carboxamide analogues. Compound **14j** possessed a chloro group at the *meta* position, which enhanced its inhibition potential compared to *p*-chloro structure **14i**. On the other hand, the bromo group in compound **14h** reduced its inhibition potential, most probably due to its big size and steric hindrance. The *para* position of the methoxy group in **14n** augmented inhibition activity compared to ortho methoxy derivative **14m**.

### 2.4. Molecular Docking

*In silico* study is a modern technique used to predict the interactions between ligand and receptor proteins. It reduces laborious lab work and provides assistance to drug discovery [30]. Molecular docking studies of synthesized carboxamide derivatives were performed via Autodock vina 1.1.2 software pack and the results displayed their *in silico* interactions [5,31]. The docked conformations of all newly synthesized derivatives of benzotriazinones occupied the same region in the active site of the enzyme pocket where the standard drug acarbose’s structure was fitted. The binding modes and orientations of the two most potent compounds of the series were studied in detail to explore their binding interactions in the active site of the target enzyme. Figure S3 clearly depicts the binding mode of standard acarbose and shows superposition of the synthesized derivatives inside the enzyme cavity.

**Figure 3:**
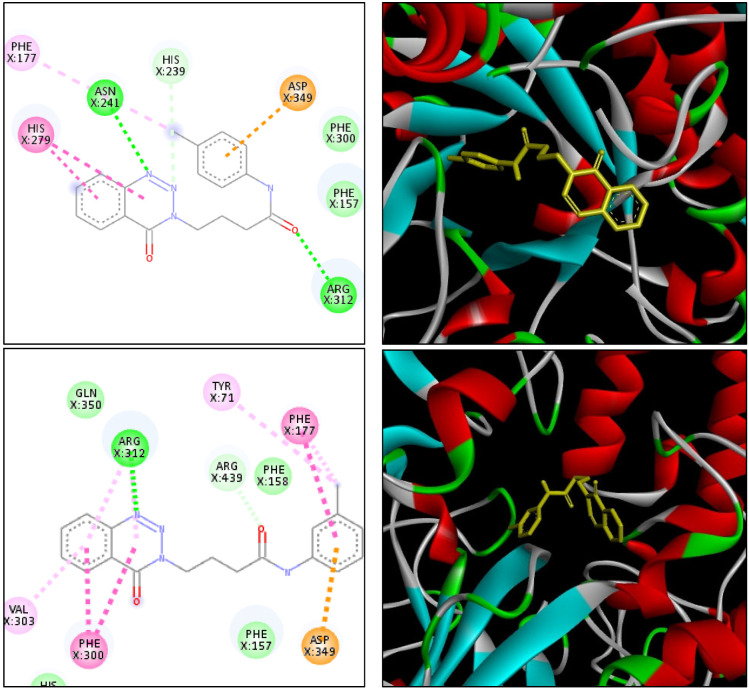
Two- and three-dimensional presentations of **14k** and **14l** inside alpha-glucosidase.

In the enzyme pocket, His279 residues were found to have hydrophobic pi–pi interactions with the benzene and the triazinone moiety of **14k**. One hydrogen bond was established by amino acid residue Asn241 with the nitrogen atom of the triazinone ring, and another nitrogen atom of the same ring formed a carbon–hydrogen bond with amino acid residue His239 (Figure 3). Oxygen of the carboxamide was found to involve in hydrogen bonding with selected residue Arg312. The benzene ring of the carboxamide developed its pi–anion interaction with Asp349 of the enzyme site (Table 3). Another hydrophobic pi–alkyl binding was observed between Phe177 and methyl residue. These results clearly demonstrated tight packing of **14k** in the selected enzyme pocket with a docking score of −9.9 Kcal/mol (Figure 3).

The ligand **14l** was found to be fit at the catalytic sites of alpha-glucosidase with a binding energy of −9.8 kcal/mol. A strong H-bond appeared between nitrogen of triazinone and Arg312 with a distance of 2.75 Å. Another residue, Arg439, developed its H-bonding with oxygen of the carbonyl group at a distance of 2.53 Å. In the ligand, there were aromatic moieties that were found to be involved in hydrophobic π-stacking interactions with Phe300 and Phe177. Benzene of carboxamide also formed pi–anion electrostatic interactions with Asp439 in the wall of the enzyme. Hydrophobic pi–alkyl interactions were also seen between alkyl, aryl, and amino acid residues Phe177, Tyr71, and val303.

### 2.5. Computational Study

#### 2.5.1. Frontier Molecular Orbital Analysis

Density frontier theory (DFT) calculations of all synthesized analogues were computed using Gaussian 09 software pack quantum chemical package. Geometrical structures were optimized at B3LYP/6-311 + G* level of theory, as reported in a previous manuscript [5,31,32]. Frontier molecular orbital (FMO) is a powerful computational tool for predicting the electrical, optical, and reactive properties of chemical structures. Quantum chemistry evaluation of the highest occupied molecular orbital (HOMO) and lowest unoccupied molecular orbital (LUMO) is known as frontier molecular orbital analysis (FMO). When an electron enters a molecule, LUMO acts as an electron acceptor, and in case of ionization, the electron is removed from HOMO. The study of HOMO–LUMO provides reactivity parameters of chemical structures like E_HOMO_, E_LUMO_, electron affinity, ionization potential, electronegativity, electrophilicity, chemical potential, dipole moment, chemical softness, and chemical hardness (Table 4). The energy difference (Egap) between HOMO and LUMO illustrates compound reactivity and kinetic stability. Among all series, structures **14k** and **14l** were found to have small energy gap values, revealing that these compounds are more reactive compared to others.

Ionization potential (I) is the amount of energy required to remove an electron from an isolated atom or molecule, and its higher value indicates stability. In series, compound **14k** showed the lowest ionization potential value, illustrating the reactive nature of these molecules [33]. The highest value of electron affinity in **14k** and **14l** depicts their tendency to accept electrons (Table 4). The chemical hardness (η) value shows the resistance of a molecule to changing its electronic distribution in a chemical reaction, and chemical softness (S) indicates an easily changeable electronic structure [33]. The lowest value of chemical hardness of **14k** shows its reactive nature towards biomolecules whereas the highest value of **14d** indicates poor inhibition potency.

The chemical potential (μ) presents the possibility of a chemical reaction in terms of negative value. A lower negative (more negative) value indicates the tendency of a molecule to accept electrons but **14k** and **14l** have comparable values to other analogues. Electrophilicity is a predictor of the electrophilic nature of a molecule, demonstrating its tendency to accepts electrons. The higher value of electrophilicity of **14k** and **14l** proved their high affinity towards other molecules. Least active compounds like **14b** and **14d** were found to be weak inhibitors with low electrophilicity values. Dipole moment presents charge delocalization on a molecule and the compounds **14k** and **14l** presented low values compared to other molecules [34].

From electronic parameters, it was observed that **14a**–**14g** have the same ionization potential, electron affinity, and chemical hardness and softness. But in the case of **14g**–**14n**, different groups are present on the benzene ring and their position affects electronic functions. Compound **14i** has a chloro group at the *para* position and its chemical hardness is 2.17 eV, but **14j** with *m*-chloro shows a value of 2.16 eV. The *para*-methylated compound **14k** indicates chemical hardness of 2.14 eV and the *meta*-methylated hybrid **14l** possesses a value of 2.16 eV. Among methoxy group derivatives, **14m** and **14n** both showed a value of 2.17 eV.

**Figure 4:**
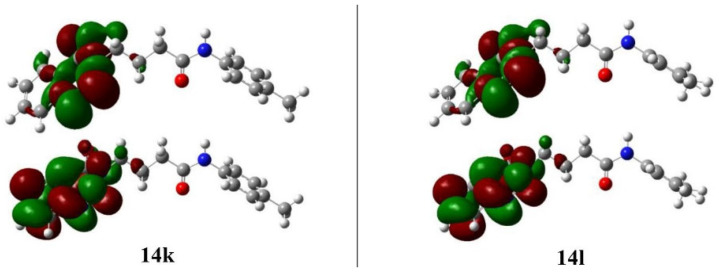
Pictorial illustration of charge density distribution in molecular orbitals of **14k**–**14l**. HOMO is shown at the bottom and LUMO is at the top.

Hence, all electronic parameters clearly indicate that **14k** and **14l** are effective candidates for inhibiting alpha-glucosidase due to high electron affinity and their reactive nature to develop interactions inside the enzyme cavity. FMO analysis clearly illustrated HOMO delocalization on the triazinone ring and LUMO electron density was found to be constrained on entire benzotriazinone rings (Figure 4 and Figure S2).

#### 2.5.2. Molecular Electrostatic Potential

**Figure 5:**
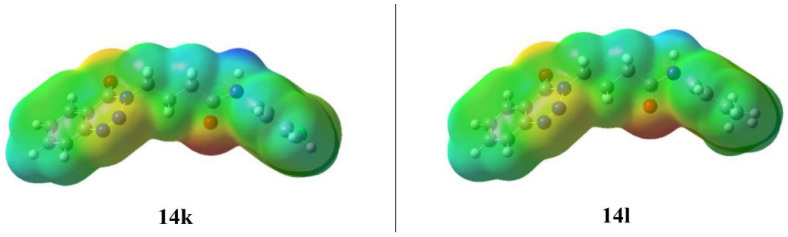
Molecular electrostatic potential mapping on the surface of **14k** and **14l**.

MEP is an effective method for predicting the distribution of electron density over the surface of a designed drug and provides information about the reactivity of molecules [35]. It also facilitates understanding of the most reactive sites of molecules towards electrophilic or nucleophilic attack [36,37]. The MEP for all benzotriazinone carboxamides was computed at B3LYP/6-311 + G* and is shown in Figure 5 and Figure S5. Different atoms and rings in a molecule contain different surface electrostatic potential values in the order red < orange < yellow < green < blue. The red color represents the most negative potential and blue color shows the most positive potential. The green color indicates the zero potential site while the yellow area appears slightly positive. Compounds **14k** and **14l** exhibited electron density at the oxygen atom of the carbonyl group and the red portion is favorable for electrophilic reactivity. The blue side of the molecule cognates to nucleophilic reactivity [38,39]. Hence, MEP mapping illustrated negative and positive sides of the molecule and indicated the tendency of the molecule to develop different interactions under the enzyme cavity [40].

## 3. Materials and Methods

### 3.1. General

Cyanuric chloride and sulfonyl chlorides were purchased from Sigma Aldrich (Burlington, MA, USA) and 1,2,3-benzotriazole was obtained from Daejung chemicals. Melting points were noted using Fisher John apparatus and FTIR was recorded using Agilent 630. ^1^H-NMR was scanned in CDCl_3_ using Bruker 400 MHz and TMS was employed as internal standard. GC-MS was recorded on MAT312.

#### 3.1.1. Isatoic Anhydride (**11**)

Isatoic anhydride was prepared using a previously developed method [29].

#### 3.1.2. 4-(4-Oxobenzo[1,2,3]triazin-3(4*H*)-yl)butanoic Acid (**12**)

4-Aminobutyric acid (2.60 g, 20 mmol) and triethyl amine (3.06 g, 20 mmol) were taken in distilled water (50 mL) and isatoic anhydride **10** was added in small portions. Mixture was stirred for 10–15 min at 60 °C and then cooled to 0 °C. Dilute HCl (30%, 20 mL) and 5 mL aqueous solution of sodium nitrite (1.70 g, 24.8 mmol) was added, and temperature was maintained at 0–5 °C. Then, it was stirred for two hours at room temperature until yellow precipitates appeared, which were filtered, washed with excess water, and dried at 70 °C. Product was recrystallized from chloroform: n-hexane mixture. Off-white powder; yield: 3.77 g (81%) m.p. > 300 °C. FTIR (v-cm^−1^): 1030 (C-N), 1595 (C=O), 1666 (C=O), 3275 (N-H). ^1^H-NMR: (400 MHz, CDCl_3_): δ_H_ 2.25 (2H, sex, *J* = 7.1 Hz, CH_2_), 2.48 (2H, t, *J* = 7.4 Hz, CH_2_), 4.54 (2H, t, *J* = 6.8 Hz, CH_2_), 7.80 (1H, t, *J* = 7.6 Hz, Ar-H), 7.93 (1H, t, *J* = 7.2 Hz, Ar-H), 8.14 (1H, d, *J* = 8.0 Hz, Ar-H), 8.34 (1H, t, *J* = 7.6 Hz, Ar-H). ^13^C-NMR: (100 MHz, CDCl_3_): δ_C_ 23.32, 32.50, 48.75, 114.37, 119.75, 120.19, 125.13, 126.35, 128.35, 130.47, 131.07, 132.51, 134.43, 144.27, 146.16, 155.74, 171.37. EI-MS: *m*/*z* calcd. for C_11_H_11_N_3_O_3_ 233 found [M-CO-H_2_O] 187.

#### 3.1.3. 3-(4-(1*H*-Benzotriazol-1-yl)-4-oxobutyl)benzo[1,2,3]triazin-4(3*H*)-one (**13**)

1,2,3-Benzotriazole (2.4 g, 20 mmol) was taken in dried tetrahydrofuran (20 mL) and thionyl chloride (0.37 mL, 5 mmol) was added dropwise at room temperature. After 30 min stirring, compound **12** was dissolved in THF (50 mL) and poured down to reaction mixture that was stirred for 48 h. Precipitates were filtered and washed with excess tetrahydrofuran. Filtrate was evaporated under reduced pressure and chloroform was added, which was washed with sodium carbonate solution (3 × 10 mL) and dried over sodium sulfate. Solution was further filtered and evaporated to derive a crude product that was recrystallized from THF.

Beige brown powder; yield: 0.93 g (56%); m.p. 129–130 °C. FT-IR (v-cm^−1^): 1030 (C-N), 1681 (C=O), 1727 (C=O). ^1^H-NMR: (400 MHz, CDCl_3_): δ_H_ 2.47–2.57 (m, 2H, CH_2_), 3.57 (t, *J* = 7.0 Hz, 2H, CH_2_), 4.69 (t, *J* = 6.8 Hz, 2H, CH_2_), 7.48 (t, *J* = 7.6 Hz, 1H, ArH), 7.62 (t, *J* = 7.8 Hz, 1H, Ar-H), 7.78 (t, *J* = 7.6 Hz, 1H, Ar-H), 7.93 (t, *J* = 7.3 Hz, 1H, Ar-H), 8.07–8.12 (m, 2H, Ar-H), 8.22 (d, *J* = 8.4 Hz, 1H, Ar-H), 8.31 (d, *J* = 7.6 Hz, 1H, Ar-H). ^13^C-NMR: (100 MHz, CDCl_3_): δ_C_ 23.32, 32.50, 48.75, 114.37, 119.75, 120.19, 125.13, 126.35, 128.35, 130.47, 131.07, 132.51, 134.43, 144.27, 146.16, 155.74, 171.37. GC-MS: *m*/*z* calcd. for C_17_H_14_N_6_O_2_ 334 found 333.

#### 3.1.4. N-alkyl/aryl-4-N-alkyl/aryl-4-(4-Oxobenzo[1,2,3]triazin-3(4*H*)-yl)butanamide(**14a**–**14n**)

Compound **13** (0.5 g, 1.49 mmol), triethylamine (0.2 mL, 1.49 mmol), and alkyl/aryl amine (1.49 mmol) were taken in dichloromethane and stirred for 10 min to 24 h according to the time required for reaction completion. Dichloromethane was evaporated under reduced pressure and residue was washed with sodium carbonate solution to remove benzotriazole. All products were recrystallized from methanol.

*N-propyl-4-(4-Oxobenzo*[1,2,3]*triazin-3(4H)-yl)butanamide* (**14a**). The compound **14a** was obtained from the reaction of **13** (0.5 g, 1.49 mmol) with propylamine (0.11 mL, 1.49 mmol). Off-white powder; yield: 0.41 g (91%); m.p. 127–128 °C. FTIR (v-cm^−1^): 1007 (C-N), 1640 (C=O), 1683 (C=O), 2960 (C-H), 3287 (N-H). ^1^H-NMR: (400 MHz, CDCl_3_): δ_H_ 0.76 (t, *J* = 7.4 Hz, 3H, CH_3_), 1.31 (sex, *J* = 6.5 Hz, 2H, CH_2_), 1.91 (quin, *J* = 7.16 Hz, 2H, CH_2_), 2.13 (t, *J* = 7.25 Hz, 2H, CH_2_), 2.88 (q, *J* = 6.5 Hz, 2H, CH_2_), 4.34 (t, *J* = 7.0 Hz, 2H, CH_2_), 7.76 (t, *J* = 5.0Hz, 1H, NH), 7.89 (dt, *J* = 6.7, 1.5 Hz, 1H, Ar-H), 8.04 (dt, *J* = 6.2, 1.25 Hz, 1H, Ar-H), 8.16 (d, *J* = 8.5 Hz, 1H, Ar-H), 8.22 (dd, *J* = 6.5, 1.5 Hz, 1H, Ar-H). ^13^C-NMR: (100 MHz, CDCl_3_): δ_C_ 11.49, 22.39, 24.48, 32.47, 40.36, 49.05, 119.36, 124.67, 128.02, 132.97, 135.45, 143.81, 154.93, 171.23. GC-MS: *m*/*z* calcd. for C_14_H_18_N_4_O_2_ 274 found [M + 1] 275.

*N-Butyl-4-(4-oxobenzo*[1,2,3]*triazin-3(4H)-yl)butanamide* (**14b**). The compound **14b** was obtained from the reaction of **13** (0.5 g, 1.49 mmol) with butylamine (0.14 mL, 1.49 mmol). Lustrous buff brown powder; yield: 0.42 (85%); m.p. 123–124 °C. FT-IR (v-cm^−1^): 1008 (C-N), 1641 (C=O), 1685 (C=O), 2930 (C-H), 3304 (N-H). ^1^H-NMR: (400 MHz, CDCl_3_): δ_H_ 0.79 (t, *J* = 7.0 Hz, 3H, CH_3_), 1.17–1.26 (m, 6H, CH_2_), 2.00 (quin, *J* = 7.5 Hz, 2H, CH_2_), 2.11 (t, *J* = 7.25 Hz, 2H, CH_2_), 4.33 (t, *J* = 7.0 Hz, 2H, CH_2_), 7.75 (t, *J* = 6.0 Hz, 1H, N-H), 7.90 (t, *J* = 7.5 Hz, 1H, Ar-H), 8.05 (t, *J* = 7.3 Hz, 1H, Ar-H), 8.16 (d, *J* = 8.5 Hz, 1H, Ar-H), 8.22 (d, *J* = 7.5Hz, 1H, Ar-H). ^13^C-NMR: (100 MHz, CDCl_3_): δ_C_ 14.03, 19.96 (2C), 30.95 (2C), 39.11, 48.68, 103.44, 121.87, 122.28, 130.37 (2C), 136.22, 153.63, 155.56. GC-MS: *m*/*z* calcd. for C_15_H_20_N_4_O_2_ 288 found 288.

*N-pentyl-4-(4-Oxobenzo*[1,2,3]*triazin-3(4H)-yl)butanamide* (**14c**). The compound **14c** was obtained from the reaction of **13** (0.5 g, 1.49 mmol) with pentylamine (0.17 mL, 1.49 mmol). Off-white powder; yield: 0.45 g (86%); m.p. 106–107 °C. FT-IR (v-cm^−1^): 1012 (C-N), 1644 (C=O), 1686 (C=O), 2922 (C-H), 3291 (N-H). ^1^H-NMR: (500 MHz, DMSO): δ_H_ 0.80 (t, *J* = 7.0 Hz, 3H, CH_3_), 1.11–1.25 (m, 4H, CH_2_), 1.29 (quin, *J* = 6.9 Hz, 2H, CH_2_), 2.01 (quin, *J* = 7.25 Hz, 2H, CH_2_), 2.12 (t, *J* = 7.3 Hz, 2H, CH_2_), 2.91 (q, *J* = 7.25 Hz, 2H, CH_2_), 4.34 (t, *J* = 6.9 Hz, 2H, CH_2_), 7.74 (t, *J* = 5.5 Hz, 1H, N-H), 7.88 (dt, *J* = 6.5, 1.0 Hz, 1H, Ar-H), 8.06 (dt, *J* = 6.3, 1.5 Hz, 1H, Ar-H), 8.15 (d, *J* = 8.0 Hz, 1H, Ar-H), 8.22 (dd, *J* = 7.0, 1.0 Hz, 1H, Ar-H). ^13^C-NMR: (100 MHz, CDCl_3_): δ_C_ 14.42, 22.33, 24.87, 29.10, 29.21, 32.938, 38.93, 49.484, 119.77, 125.09, 128.44, 133.39, 135.87, 144.22, 155.35, 171.59. GC-MS: *m*/*z* calcd. for C_16_H_22_N_4_O_2_ 302 found 302.

*N-Hexyl-4-(4-oxobenzo*[1,2,3]*triazin-3(4H)-yl)butanamide* (**14d**). The compound **14d** was obtained from the reaction of **13** (0.5 g, 1.49 mmol) with hexyl amine (0.19 mL, 1.49 mmol). Lustrous off-white powder; yield: 0.047 g (85%); m.p. 102–103 °C. FT-IR (v-cm^−1^): 1010 (C-N), 1643 (C=O), 1685 (C=O), 2923 (C-H), 3307 (N-H), ^1^H-NMR: (400 MHz, CDCl_3_): δ_H_ 0.86 (t, *J* = 7.0 Hz, 3H, CH_3_), 1.27–1.34 (m, 6H, CH_2_), 1.48–1.51 (m, 2H, CH_2_), 2.80–2.83 (m, 4H, CH_2_), 3.23 (q, *J* = 5.9 Hz, 2H, CH_2_), 4.52 (t, *J* = 6.0 Hz, 2H, CH_2_), 6.07 (s, 1H, NH), 7.80 (dt, *J* = 6.4, 1.0 Hz, 1H, Ar-H), 7.96 (dt, *J* = 6.4, 1.2 Hz, 1H, Ar-H), 8.15 (d, *J* = 8.0 Hz, 1H, Ar-H), 8.34 (dd, *J* = 7.4, 1.0 Hz, 1H, Ar-H). ^13^C-NMR: (100 MHz, CDCl_3_): δ_C_ 14.04, 22.57, 25.29, 26.62, 29.52, 31.48, 33.34, 39.83, 48.93, 119.71, 125.12, 128.37, 132.53, 134.98, 144.29, 155.95, 171.86. GC-MS: *m*/*z* calcd. for C_17_H_24_N_4_O_2_ 316 found 316.

*N-Heptyl-4-(4-oxobenzo*[1,2,3]*triazin-3(4H)-yl)butanamide* (**14e**). The compound **14e** was obtained from the reaction of **13** (0.5 g, 1.49 mmol) with heptylamine (0.22 mL, 1.49 mmol). Lustrous off powder; yield: 0.49 g (83%); m.p. 100–101 °C. FT-IR (v-cm^−1^): 1011 (C-N), 1643 (C=O), 1685 (C=O), 2925 (C-H), 3300 (N-H). ^1^H-NMR: (400 MHz, CDCl_3_): δ_H_ 0.851 (t, *J* = 6.6 Hz, 3H, CH_3_), 1.24–1.27 (m, 10H, CH_2_), 1.47–1.50 (m, 4H, CH_2_), 3.21 (q, *J* = 6.5 Hz, 2H, CH_2_), 4.52 (t, *J* = 6.0 Hz, 2H, CH_2_), 6.0 (s, 1H, N-H), 7.79 (t, *J* = 7.4 Hz, 1H, Ar-H), 7.94 (t, *J* = 7.8 Hz, 1H, Ar-H), 8.14 (d, *J* = 8.0 Hz, 1H, Ar-H), 8.33 (d, *J* = 7.6 Hz, 1H, Ar-H). ^13^C-NMR: (100 MHz, CDCl_3_): δ_C_ 14.08, 22.60, 25.27, 26.91, 28.97, 29.58, 31.75, 33.41, 39.76, 48.95, 119.72, 125.11, 128.35, 132.50, 134.96, 144.28, 155.90, 171.75. GC-MS: *m*/*z* calcd. for C_18_H_26_N_4_O_2_ 330 found 330.

*N-Octyl-4-(4-oxobenzo*[1,2,3]*triazin-3(4H)-yl)butanamide* (**14f**). The compound **14f** was obtained from the reaction of **13** (0.5 g, 1.49 mmol) with octylamine (0.25 mL, 1.49 mmol). Off-white powder; yield: 0.51 g (84%); m.p. 95°–96 °C. FT-IR (v-cm^−1^): 1010 (C-N), 1643 (C=O), 1684 (C=O), 2921 (C-H), 3304 (N-H). ^1^H-NMR (400 MHz, CDCl_3_): δ_H_ 0.84 (t, *J* = 6.8 Hz, 3H, CH_3_), 1.23–1.26 (m, 10H, CH_2_), 1.47 (quin, *J* = 6.8 Hz, 2H, CH_2_), 2.24–2.27 (m, 4H, CH_2_), 3.20 (q, *J* = 7.0 Hz, 2H, CH_2_), 4.51 (t, *J* = 6.0 Hz, 2H, CH_2_), 5.92 (s, 1H, NH), 7.89 (dt, *J* = 7.0, 1.0Hz, 1H, Ar-H), 7.93 (dt, *J* = 7.0, 1.2 Hz, 1H, Ar-H), 8.13 (d, *J* = 8.4 Hz, 1H, Ar-H), 8.32 (dd, *J* = 7.6, 0.08 Hz, 1H, Ar-H). ^13^C-NMR: (100 MHz, CDCl_3_): δ_C_ 14.10, 22.65, 25.25, 26.96, 29.22, 29.27, 29.61, 31.80, 33.50, 39.69, 48.98, 119.74, 125.11, 128.33, 132.48, 134.93, 144.28, 155.85, 171.61. GC-MS: *m*/*z* calcd. for C_19_H_28_N_4_O_2_ 344 found 344.

*N-(3,5-Dimethylphenyl)-4-(4-oxobenzo*[1,2,3]*triazin-3(4H)-yl)butanamide* (**14g**). The compound **14g** was obtained from the reaction of **13** (0.5 g, 1.49 mmol) with 3,5- dimethylaniline (0.18 g, 1.49 mmol). Beige brown powder; yield: 0.37 g (75%); m.p. 110–111 °C. FT-IR (v-cm^−1^): 1008 (C-N), 1656 (C=O), 1687 (C=O), 2909 (C-H), 3277 (N-H). ^1^H-NMR: (400 MHz, CDCl_3_): δ_H_ 2.25–2.44 (m, 10H, CH_3_, CH_2_), 4.60 (t, *J* = 5.6 Hz, 2H, CH_2_), 6.71 (s, 1H, Ar-H), 7.15 (s, 2H, Ar-H), 7.79 (dt, *J* = 7.2, 0.8 Hz, 1H, Ar-H), 7.94 (dt, *J* = 7.0, 1.4 Hz, 1H, Ar-H), 7.98 (s, 1H, N-H), 8.22 (d, *J* = 8.0 Hz, 1H, Ar-H), 8.35 (d, *J* = 6.8 Hz, 1H, Ar-H). ^13^C-NMR: (100 MHz, CDCl_3_): δ_C_ 21.39 (2CH_3_), 25.54, 34.54, 48.80, 114.42, 117.62 (2CH), 119.64, 125.18, 126.00, 128.36, 132.57, 135.03, 137.82, 138.63 (2C), 156.20, 170.26. GC-MS: *m*/*z* calcd. for C_19_H_20_N_4_O_2_ 336 found 338.

*N-(4-Bromophenyl)-4-(4-oxobenzo*[1,2,3]*triazin-3(4H)-yl)butanamide* (**14h**). The compound **14h** was obtained from the reaction of **13** (0.5 g, 1.49 mmol) with 4-bromoaniline (0.25 g, 1.49 mmol). White powder; yield: 0.4 g (71%); m.p. 115–116 °C. FT-IR (v-cm^−1^): 1008 (C-N), 1656 (C=O), 1684 (C=O), 2946 (C-H), 3259 (N-H). ^1^H-NMR: (400 MHz, CDCl_3_): δ_H_ 2.16–2.54 (m, 4H, CH_2_), 4.60 (t, *J* = 6.0 Hz, 2H, CH_2_), 7.03–7.50 (m, 4H, Ar-H), 7.78–7.83 (m, 2H, Ar-H, N-H), 7.95 (dt, *J* = 7.0, 1.4 Hz, 1H, Ar-H), 8.15 (d, *J* = 8.0 Hz, 1H, Ar-H), 8.35 (d, *J* = 8.0 Hz, 1H, Ar-H). ^13^C-NMR: (100 MHz, CDCl_3_): δ_C_ 25.58, 34.53, 48.78, 117.35, 119.69, 121.19, 125.20, 128.40, 129.92, 131.90, 132.55, 135.12 (2CH), 137.17, 144.30, 156.40, 170.35. GC-MS: *m*/*z* calcd. for C_17_H_15_BrN_4_O_2_ 387 found 388.

*N-(4-Chlorophenyl)-4-(4-oxobenzo*[1,2,3]*triazin-3(4H)-yl)butanamide* (**14i**). The compound **14i** was obtained from the reaction of **13** (0.5 g, 1.49 mmol) with 4- chloroaniline (0.19 g, 1.49 mmol). Off-white powder; yield: 0.34 g (69%); m.p. 144–145 °C. FT-IR (v-cm^−1^): 1009 (C-N), 1656 (C=O), 1687 (C=O), 2927 (C-H), 3281 (N-H). ^1^H-NMR: (400 MHz, CDCl_3_): δ_H_ 2.37–2.42 (m, 4H, CH_2_), 4.59 (t, *J* = 6.0 Hz, 2H, CH_2_), 7.23 (d, *J* = 6.8 Hz, Ar-H, 2H), 7.52 (d, *J* = 6.8 Hz, 2H, Ar-H), 7.81 (t, *J* = 7.2 Hz,1H, Ar-H), 7.94 (t, *J* = 7.2 Hz, 1H, Ar-H), 8.14 (d, *J* = 8.0 Hz, 1H, Ar-H), 8.26 (s, 1H, NH), 8.35 (d, *J* = 7.2Hz, 1H, Ar-H). ^13^C-NMR: (100 MHz, CDCl_3_): δ_C_ 25.62, 34.65, 48.80, 119.55, 121.05 (2CH), 125.18, 128.45, 128.95 (2CH), 129.05, 132.74, 135.18, 136.70, 144.26, 156.39, 170.33. GC-MS: *m*/*z* calcd. for C_17_H_15_ClN_4_O_2_ 342 found 341.

*N-(3-Chlorophenyl)-4-(4-oxobenzo*[1,2,3]*triazin-3(4H)-yl)butanamide* (**14j**). The compound **14j** was obtained from the reaction of **13** (0.5 g, 1.49 mmol) with 3-chloroaniline (0.19 g, 1.49 mmol). Yellow crystals; yield: 0.36 g (71%); m.p. 209–210 °C. FT-IR (v-cm^−1^): 1012 (C-N), 1661 (C=O), 1689 (C=O), 2986 (C-H), 3332 (NH). ^1^H-NMR: (400 MHz, CDCl_3_): δ_H_ 2.37–2.44 (m, 4H, CH_2_), 4.60 (t, *J* = 5.8 Hz, 2H, CH_2_), 7.05 (dd, *J* = 1.0 Hz, *J* = 6.8Hz, 1H, Ar-H), 7.21 (t, *J* = 8.0 Hz, 2H, Ar-H), 7.41–7.43 (m, 1H, Ar-H), 7.67 (s, 1H, N-H,), 7.82 (dt, *J* = 7.0, 1.4 Hz, 1H, Ar-H), 7.96 (dt, *J* = 6.0, 1.4 Hz, 1H, Ar-H), 8.16 (d, *J* = 8.2 Hz, 1H, Ar-H), 8.37 (dd, *J* = 8.0, 1.2 Hz, 1H, Ar-H). ^13^C-NMR: (100 MHz, CDCl_3_): δ_C_ 25.62, 34.52, 48.65, 117.66, 119.53, 119.77, 124.18, 125.19, 128.44, 129.93, 132.74, 134.59, 135.19, 139.24, 144.26, 156.46, 170.34. GC-MS: *m*/*z* calcd. for C_17_H_15_ClN_4_O_2_ 342 found 341.

*N-(4-methylphenyl)-4-(4-Oxobenzo*[1,2,3]*triazin-3(4H)-yl)butanamide* (**14k**). The compound **14k** was obtained from the reaction of **13** (0.5 g, 1.49 mmol) with 4-methylaniline (0.15 g, 1.49 mmol). Tan brown powder; yield: 0.35 g (73%); m.p. 175–176 °C. FT-IR (v-cm^−1^): 1012 (C-N), 1664 (C=O), 1673 (C=O), 2922 (C-H), 3482 (N-H). ^1^H-NMR: (400 MHz, CDCl_3_,): δ_H_ 2.30–2.42 (m, 7H, CH_2_), 4.57 (t, 2H, CH_2_), 7.08 (d, *J* = 6.0 Hz, 2H, Ar-H), 7.20 (d, *J* = 6.0 Hz, 2.0 Hz, 1H, Ar-H), 7.80 (t, *J* = 6.0 Hz, 1H, Ar-H), 7.94 (t, *J* = 6.0 Hz, 1H, Ar-H), 8.10 (s, 1H, N-H), 8.14 (d, *J* = 7.2 Hz, 1H, Ar-H), 8.35 (d, *J* = 5.6 Hz, 1H, Ar-H). ^13^C-NMR: (100 MHz, CDCl_3_): δ_C_ 21.54, 25.53, 34.58, 48.83, 116.91, 119.63, 120.46, 125.00, 125.18, 128.39, 128.77, 132.62, 135.07, 137.96, 138.85, 144.27, 156.22, 170.19. GC-MS: *m*/*z* calcd. for C_18_H_18_N_4_O_2_ 322 found 321.

*N-(3-methylphenyl)-4-(4-Oxobenzo*[1,2,3]*triazin-3(4H)-yl)butanamide* (**14l**). The compound **14l** was obtained from the reaction of **13** (0.5 g, 1.49 mmol) with 3-methylaniline (0.15 g, 1.49 mmol). Light brown powder; yield: 0.34 g (72%); m.p. 105–106 °C. FT-IR (v-cm^−1^): 1008 (C-N), 1656 (C=O), 1687 (C=O), 2923 (C-H), 3281 (N-H). ^1^H-NMR: (400 MHz, CDCl_3_): δ_H_ 2.30–2.42 (m, 7H, CH_2_), 4.57–4.60 (m, CH_2_, 2H), 6.89 (s, 1H, Ar-H), 7.16–7.39 (m, Ar-H, 3H), 7.79- 8.03 (m, Ar-H, N-H, 3H), 8.13 (d, *J* = 6.8 Hz, Ar-H, 1H), 8.34 (d, *J* = 6.0 Hz, 1H, Ar-H) ^13^C-NMR: (100 MHz, CDCl_3_): δ_C_ 20.92, 25.59, 34.53, 48.89, 119.63, 120.03, 125.20, 128.41, 129.47, 132.50, 132.67, 133.89, 134.93, 135.10, 135.40, 144.27, 156.23, 170.26. GC-MS: *m*/*z* calcd. for C_18_H_18_N_4_O_2_ 322 found 323.

*N-(2-Methoxyphenyl)-4-(4-oxobenzo*[1,2,3]*triazin-3(4H)-yl)butanamide* (**14m**). The compound **14m** was obtained from the reaction of **13** (0.5 g, 1.49 mmol) with 2-methoxyaniline (0.18 g, 1.49 mmol). White powder; yield: 0.38 g (76%); m.p. 139–140 °C. FT-IR (v-cm^−1^): 1008 (C-N), 1656 (C=O), 1678 (C=O), 2835 (C-H), 3004 (N-H). ^1^H-NMR: (400 MHz, CDCl_3_): δ_H_ 2.36 (quin, *J* = 6.8 Hz, 2H, CH_2_), 2.51 (t, *J* = 7.4 Hz, 2H, CH_2_), 3.86 (s, 3H, CH_3_), 4.59 (t, *J* = 6.4 Hz, Ar-H, 2H), 6.83 (d, *J* = 8.0 Hz, 1H, Ar-H), 6.88 (t, *J* = 7.2 Hz, 1H, Ar-H), 6.99 (t, *J* = 7.4 Hz, 1H, Ar-H), 7.78 (t, *J* = 7.2 Hz, 1H, Ar-H), 7.89- 7.93 (m, 2H, NH, Ar-H), 8.11 (d, *J* = 8.0 Hz, 1H, Ar-H), 8.26 (d, *J* = 8.0 Hz, 1H, Ar-H), 8.32 (d, *J* = 8.0 Hz, 1H, Ar-H). ^13^C-NMR: (100 MHz, CDCl_3_): δ_C_ 25.56, 34.32, 48.81, 55.52, 114.12 (2C), 119.63, 121.66 (2C), 125.17, 128.40, 131.18, 132.63, 135.08, 144.28, 156.23, 156.36, 170.00. GC-MS: *m*/*z* calcd. for C_18_H_18_N_4_O_3_ 338 found 322.

*N-(4-Methoxyphenyl)-4-(4-oxobenzo*[1,2,3]*triazin-3(4H)-yl)butanamide* (**14n**). The compound **14n** was obtained from the reaction of **13** (0.5 g, 1.49 mmol) with 4- methoxyaniline (0.18 g, 1.49 mmol). White powder; yield: 0.40 g (80%); m.p. 147–148 °C. FT-IR (v-cm^−1^): 1006 (C-N), 1656 (C=O), 1678 (C=O), 2938 (C-H), 3250 (N-H). 1H-NMR: (400 MHz, CDCl3): δH 2.36–2.42 (m, 4H, CH2), 3.77 (s, 3H, CH3), 4.60 (t, J = 5.4 Hz, 2H, CH2), 6.82 (d, J = 8.0 Hz, 2H, Ar-H), 7.45 (d, J = 8.0 Hz, 2H, Ar-H), 7.80 (t, J = 7.6 Hz, 1H, Ar-H), 7.95 (t, J = 8.0Hz, Ar-H, 1H), 7.99 (s, 1H, N-H), 8.14 (d, J = 8.0 Hz, 1H, Ar-H), 8.35 (d, J = 8.0 Hz, 1H, Ar-H). 13C-NMR: (100 MHz, CDCl3): δC 24.87, 34.75, 49.04, 55.70, 119.92, 119.80, 119.87, 121.05, 123.68, 125.10, 127.59, 128.30, 132.42, 134.86, 144.29, 147.83, 155.74, 169.81. GC-MS: *m*/*z* calcd. for C18H18N4O3 338 found 337.

### 3.2. Procedure for In Vitro Study

The alpha-glucosidase inhibitory assay was performed by adopting the method of Pierre et al., with minor modifications. Alpha-glucosidase (Cat No. 5003-1KU Type I) belonging to Saccharomyces cerevisiae was selected for this protocol because its structure and function were similar to yeast and mammalian enzymes. In test tubes, 10 μL of test compound (0.5 mM), 70 μL of saline phosphate buffer (50 mM) to adjust pH at 6.8, and 10 μL of α-glucosidase enzyme (0.0234 units) were added to prepare 100 μL assay mixture. A quantity of 10 μL p-nitrophenyl-α-D-glucopyranoside (0.5 mM, N1377 from Sigma, Burlington, MA, USA) was added to initiate the reaction and tubes were incubated for 30 min. Acarbose was used as a positive control. Free substrate change in absorbance was monitored at 400 nm. The alpha-glucosidase inhibitory activity was calculated as percentage inhibition by using formula:

% Inhibition = [(Abs. of control − Abs. of test)/Abs. of control] × 100

IC_50_ values were determined using EZ-Fit enzyme kinetics software version 5.03 (Perrella Scientific Inc. Amherst, Amherst, MA, USA).

### 3.3. Molecular Docking Protocol

In order to explore the most probable way of binding of synthesized hybrids inside active sites of *α*-glucosidase, Autodock Vina software (v.1.1.2.) along with its tools (Mgltools V.1.5.6) were employed to carry out molecular docking studies. The crystal structure of eukaryotic yeast (Saccharomyces cerevisiae) was not found in the Protein Data Bank; only some bacterial glucosidase structures were available. The sequence of Saccharomyces cerevisiae’s α-glucosidase is based on sequence of 584 amino acid residues (uniport ID: P53341). NCBI’s BLAST algorithm was used as a suitable template for homology modelling of target protein. For homology modeling, highest sequence similarity was observed in oligo-1,6-glucosidase (P53051) and selected to be used as a basic pattern. Sequence alignment was conducted by using Needleman–Wunsch Global Alignment Algorithm via Chimera (v.1.17.1). Structure modelling was processed using Modeller. Quality of the new structure was checked by using Ramachandran plot, which showed 97.3% of residues were in the favored region, and 99.7% of residues were in the allowed region. To review the quality of created homology model, molecular dynamics simulation was carried out using NAMD. Visualization of molecular dynamics trajectories was carried out using VMD. Protein molecule was solvated and equilibrated in a water box and modeled at physiological temperature of 310 K for 10 ps. Finally, optimized structure was applied for further molecular docking studies using Autodock Vina software pack. The optimized structure of all newly synthesized compounds at B3LYP/6-311 + G* level of theory were employed for docking studies using our previously reported molecular docking parameters [41]. The compounds having highest binding affinity were chosen to explore their binding interactions with amino acid residues of protein structure. Discovery Studio visualizer (v21.1.0.20298) was used for presentation of docked conformations and visualization of binding interactions inside the active pocket of receptor protein.

### 3.4. Computational Method

Becke’s three-parameter hybrid exchange functionals [42] and Lee–Yang–Parr correlation functionals (B3LYP) method along with 6-311 + G* basis set was employed for computational study [43,44]. Gaussian 09 software pack was used to perform all the computational calculations. To ensure that the optimized geometry corresponds to the equilibrium (minimum energy) structure, harmonic vibrational frequency analysis was applied at the same basic set level to detect any imaginary frequencies. All docked structures were utilized to explain QM descriptors. The Gauss view software package (v.5.0) was used to visualize the computed structures including HOMO, LUMO, and molecular electrostatic potential (MEP) representations. In addition, different electronic parameters were calculated by using Equations (1)–(7).

Ionization potential (I) = −E_HOMO_ (1)

Electron affinity (A) = −E_LUMO_ (2)

Chemical hardness (η) = (E_LUMO_ − E_HOMO_)/2 (3)

Chemical softness (S) = 1/2η(4)

Chemical potential (μ) = −[(E_HOMO_ + E_LUMO_)/2] (5)

Electronegativity (χ) = (E_HOMO_ + E_LUMO_)/2 (6)

Electrophilicity (ω) = (E_HOMO_ + E_LUMO_/2)^2^/2η (7)

## 4. Conclusions

A series of benzotriazinone carboxamides was prepared at room temperature and characterized using different spectroscopic techniques including FTIR, ^1^H-NMR, ^13^C-NMR, and EI-MS. The alpha-glucosidase inhibition activity of all analogues was examined using an *in vitro* assay. Compounds **14k** and **14l** showed remarkable alpha-glucosidase inhibitory potential compared to the market drug acarbose. Molecular studies presented good docking scores with significant interactions between ligands and targeted receptor pockets. Frontier molecular orbital analysis of all hybrids predicted the reactive nature of **14k** and **14l** with lowest energy gap values between HOMO and LUMO. In electronic properties, lower values of chemical hardness and higher values of electron affinity and electrophilicity demonstrated the tendency of these compounds to develop interactions with biomolecules. An electrostatic potential map of all analogues was investigated to understand the electrophilic and nucleophilic regions of molecules. All *in silico* findings were found to be in good agreement with the *in vitro* results. Hopefully, this work may be useful for the optimization and structural modification of new antidiabetic drugs.

## Figures and Tables

**Table 2:** Results of alpha-glucosidase inhibition studies.

Sr. No.	Codes	Inhibition (%) at 0.5 mM	IC_50_ (μM)
1.	**14a**	39.13 ± 0.19	-
2.	**14b**	67.53 ± 0.14	243.41 ± 0.18
3.	**14c**	42.34 ± 0.25	-
4.	**14d**	66.20 ± 0.11	335.04 ± 0.07
5.	**14e**	40.23 ± 0.21	-
6.	**14f**	30.57 ± 0.16	-
7.	**14g**	78.32 ± 0.18	196.52 ± 0.08
8.	**14h**	74.25 ± 0.27	238.15 ± 0.15
9.	**14i**	82.77 ± 0.20	163.29 ± 0.13
10.	**14j**	89.35 ± 0.21	52.01 ± 0.07
11.	**14k**	94.18 ± 0.23	27.13 ± 0.12
12.	**14l**	92.63 ± 0.12	32.14 ± 0.11
13.	**14m**	84.31 ± 0.18	121.26 ± 0.15
14.	**14n**	87.46 ± 0.15	75.37 ± 0.14
15.	Acarbose	92.23 ± 0.16	37.38 ± 0.12

**Table 3:** Types of forces between ligand and enzyme pockets.

Ligand	Amino Acid	Distance (Å)	Attractive Forces
**14k**	Asn241	2.38	Hydrogen bond
**14k**	Arg312	2.57	Hydrogen bond
**14k**	His239	2.94	Hydrogen bond
**14k**	Asp349	4.75	Electrostatic
**14k**	His279	4.14	Hydrophobic
**14k**	His279	5.15	Hydrophobic
**14k**	Phe177	5.37	Hydrophobic
**14l**	Arg312	2.75	Hydrogen bond
**14l**	Arg439	2.53	Hydrogen bond
**14l**	Asp349	4.14	Electrostatic
**14l**	Phe300	4.44	Hydrophobic
**14l**	Phe300	4.98	Hydrophobic
**14l**	Phe177	4.99	Hydrophobic
**14l**	Tyr71	4.19	Hydrophobic
**14l**	Val303	5.04	Hydrophobic
**14l**	Arg313	5.37	Hydrophobic
**14l**	Tyr71	5.11	Hydrophobic
**14l**	Phe177	4.61	Hydrophobic

**Table 4:** Electronic properties of all synthesized carboxamides (**14a**–**14n**).

Compounds	E_HOMO_	E_LUMO_	E_gap_	I	A	η	S	μ	χ	ω	D
**14a**	−6.24	−1.92	4.33	6.24	1.92	2.16	0.23	−4.08	4.08	3.85	1.58
**14b**	−6.24	−1.91	4.33	6.24	1.91	2.16	0.23	−4.08	4.08	3.84	1.52
**14c**	−6.24	−1.91	4.33	6.24	1.91	2.16	0.23	−4.08	4.08	3.84	1.58
**14d**	−6.24	−1.91	4.33	6.24	1.91	2.16	0.23	−4.07	4.07	3.84	1.54
**14e**	−6.24	−1.91	4.33	6.24	1.91	2.16	0.23	−4.07	4.07	3.84	1.57
**14f**	−6.24	−1.91	4.33	6.24	1.91	2.16	0.23	−4.07	4.07	3.84	1.51
**14g**	−6.25	−1.92	4.33	6.25	1.92	2.16	0.23	−4.08	4.08	3.85	1.02
**14h**	−6.25	−1.92	4.34	6.25	1.92	2.17	0.23	−4.08	4.08	3.85	2.99
**14i**	−6.26	−1.92	4.34	6.26	1.92	2.17	0.23	−4.09	4.09	3.85	3.16
**14j**	−6.25	−1.92	4.33	6.25	1.92	2.16	0.23	−4.09	4.09	3.86	2.80
**14k**	−6.22	−1.93	4.29	6.22	1.93	2.14	0.23	−4.08	4.08	3.88	1.10
**14l**	−6.25	−1.93	4.32	6.25	1.93	2.16	0.23	−4.09	4.09	3.87	1.20
**14m**	−6.25	−1.92	4.33	6.25	1.92	2.17	0.23	−4.08	4.08	3.85	2.06
**14n**	−6.24	−1.91	4.33	6.24	1.91	2.17	0.23	−4.07	4.07	3.83	2.77

All electronic parameters in eV units except dipole moment, measured in Debye.

## Data Availability

The data presented in this study are available in the Supplementary Materials. Samples of the compounds are available from the authors.

## Supplementary Materials

The following supporting information are available online: FT-IR, ^1^H-NMR, ^13^CNMR, and MS spectra (Figures S6–S68) of all alpha-glucosidase inhibitors can be downloaded at https://www.mdpi.com/article/10.3390/molecules28186623/s1. Molecular docking 2D/3D maps (Figures S1–S3, Table S1), charge density distribution in molecular orbitals (Figure S4), and MEP mapping (Figure S5) of all synthesized compounds can be found in the Supplementary File.

## Sample Availability

Samples of the compounds are available from the authors.

## References

[1] Raut S.K., Khullar M. (2023). Oxidative stress in metabolic diseases: Current scenario and therapeutic relevance. Mol. Cell. Biochem..

[2] Borges L.D., Dias H.H., de Souza Ferreira E., Alves P.M., Silva B.O., de Paiva Santos K., da Costa G.D., Moreira T.R., Santos D.S., Cotta R.M.M. (2023). Prevalence of Diabetes Mellitus among individuals with chronic kidney disease: Systematic review and meta-analysis. J. Evid. Based Healthc..

[3] Hussain F., Hafeez J., Khalifa A.S., Naeem M., Ali T., Eed E.M. (2022). In vitro and in vivo study of inhibitory potentials of α-glucosidase and acetylcholinesterase and biochemical profiling of *M. charantia* in alloxan-induced diabetic rat models. Am. J. Transl. Res..

[4] Farwa U., Raza M.A. (2022). Heterocyclic compounds as a magic bullet for diabetes mellitus: A review. RSC Adv..

[5] Khalid Z., Alnuwaiser M.A., Ahmad H.A., Shafqat S.S., Munawar M.A., Kamran K., Abbas M.M., Kalam M.A., Ewida M.A. (2022). Experimental and Computational Analysis of Newly Synthesized Benzotriazinone Sulfonamides as Alpha-Glucosidase Inhibitors. Molecules.

[6] Gondaliya P., Sayyed A.A., Bhat P., Mali M., Arya N., Khairnar A., Kalia K. (2022). Mesenchymal stem cell-derived exosomes loaded with miR-155 inhibitor ameliorate diabetic wound healing. Mol. Pharm..

[7] Elahabaadi E., Salarian A.A., Nassireslami E. (2022). Design, synthesis, and molecular docking of novel hybrids of coumarin-dithiocarbamate alpha-glucosidase inhibitors targeting type 2 diabetes mellitus. Polycycl. Aromat. Compd..

[8] Pałasz A., Cież D., Trzewik B., Miszczak K., Tynor G., Bazan B. (2019). In the search of glycoside-based molecules as antidiabetic agents. Top. Curr. Chem..

[9] Sepehri N., Asemanipoor N., Mousavianfard S.A., Hoseini S., Faramarzi M.A., Adib M., Biglar M., Larijani B., Hamedifar H. (2020). New acridine-9-carboxamide linked to 1,2,3-triazole-N-phenylacetamide derivatives as potent α-glucosidase inhibitors: Design, synthesis, in vitro, and in silico biological evaluations. Med. Chem. Res..

[10] Popović-Djordjević J.B., Jevtić I.I., Grozdanić N.D., Šegan S.B., Zlatović M.V., Ivanović M.D., Stanojković T.P. (2017). α-Glucosidase inhibitory activity and cytotoxic effects of some cyclic urea and carbamate derivatives. J. Enzyme Inhib. Med. Chem..

[11] Zhang J.-P., Li Q., Zhang C., Li P., Chen L.-J., Wang Y.-H., Ruan X.H., Xiao W., Xue W. (2018). Synthesis, antibacterial, and antiviral activities of novel penta-1,4-dien-3-one derivatives containing a benzotriazin-4(3H)-one moiety. Chem. Pap..

[12] Komet M. (1997). Microwave synthesis and anticonvulsant activity of new 3-benzyl-1,2,3-benzotriazin-4(3H)-ones. J. Heterocycl. Chem..

[13] Selim Y.A., Abd El-Azim M.H., El-Farargy A.F. (2018). Synthesis and Anti-inflammatory Activity of Some New 1,2,3-Benzotriazine Derivatives Using 2-(4-Oxo-6-phenylbenzo[d][1,2,3]triazin-3(4H)-yl)acetohydrazide as a Starting Material. J. Heterocycl. Chem..

[14] El Rayes S., Ali I., Fathalla W., Mahmoud M. (2020). Synthesis and Biological Activities of Some New Benzotriazinone Derivatives Based on Molecular Docking; Promising HepG2 Liver Carcinoma Inhibitors. ACS Omega.

[15] Sorensen E.S., Macedo A.B., Resop R.S., Howard J.N., Nell R., Sarabia I., Newman D., Ren Y., Jones R.B., Planelles V. (2020). Structure-activity relationship analysis of benzotriazine analogues as HIV-1 latency-reversing agents. Antimicrob. Agents Chemother..

[16] Zhang F., Wu D., Wang G.-L., Hou S., Ou-Yang P., Huang J., Xu X.Y. (2017). Synthesis and biological evaluation of novel 1,2,3-benzotriazin-4-one derivatives as leukotriene A4 hydrolase aminopeptidase inhibitors. Chin. Chem. Lett..

[17] Moghimi S., Goli-Garmroodi F., Pilali H., Mahdavi M., Firoozpour L., Nadri H., Moradi A., Asadipour A., Shafiee A., Foroumadi A. (2016). Synthesis and anti-acetylcholinesterase activity of benzotriazinone-triazole systems. J. Chem. Sci..

[18] Reddy G.S., Snehalatha A.V., Edwin R.K., Hossain K.A., Giliyaru V.B., Hariharapura R.C., Shenoy G.G., Misra P., Pal M. (2019). Synthesis of 3-indolylmethyl substituted (pyrazolo/benzo) triazinone derivatives under Pd/Cu-catalysis: Identification of potent inhibitors of chorismate mutase (CM). Bioorg. Chem..

[19] Yan Y.-C., Wu W., Huang G.-Y., Yang W.-C., Chen Q., Qu R.-Y., Lin H.Y., Yang G.F. (2022). Pharmacophore-Oriented Discovery of Novel 1,2,3-Benzotriazine-4-one Derivatives as Potent 4-Hydroxyphenylpyruvate Dioxygenase Inhibitors. J. Agric. Food Chem..

[20] Roca-Paixão L., Correia N.T., Danède F., Guerain M., Affouard F. (2023). Carbamazepine/tartaric acid cocrystalline forms: When stoichiometry and synthesis method matter. Cryst. Growth Des..

[21] Beydoun A., Kutluay E. (2002). Oxcarbazepine. Expert Opin. Pharmacother..

[22] Vendrame M., Loddenkemper T., Gooty V.D., Takeoka M., Rotenberg A., Bergin A.M., Eksioglu Y.Z., Poduri A., Duffy F.H., Libenson M. (2010). Experience with rufinamide in a pediatric population: A single center’s experience. Pediatr. Neurol..

[23] Fleischmann R., Iqbal I., Slobodin G. (2002). Meloxicam. Expert Opin. Pharmacother..

[24] Tanimoto M., Kanazawa A., Hirose T., Yoshihara T., Kobayashi-Kimura S., Nakanishi R., Tosaka Y., Sasaki-Omote R., Kudo-Fujimaki K., Komiya K. (2015). Comparison of sitagliptin with nateglinide on postprandial glucose and related hormones in drug-naïve Japanese patients with type 2 diabetes mellitus: A pilot study. J. Diabetes Investig..

[25] Nishio S., Abe M., Ito H. (2015). Anagliptin in the treatment of type 2 diabetes: Safety, efficacy, and patient acceptability. Diabetes Metab. Syndr. Obes..

[26] Peytam F., Adib M., Shourgeshty R., Firoozpour L., Rahmanian-Jazi M., Jahani M., Moghimi S., Divsalar K., Faramarzi M.A., Mojtabavi S. (2020). An efficient and targeted synthetic approach towards new highly substituted 6-amino-pyrazolo [1,5-a]pyrimidines with α-glucosidase inhibitory activity. Sci. Rep..

[27] Fershtat L.L., Makhova N.N. (2017). Molecular hybridization tools in the development of furoxan-based NO-donor prodrugs. Chem. Eur. J..

[28] Harrison J.R., Brand S., Smith V., Robinson D.A., Thompson S., Smith A., Davies K., Mok N., Torrie L.S., Collie I. (2018). A molecular hybridization approach for the design of potent, highly selective, and brain-penetrant N-myristoyltransferase inhibitors. J. Med. Chem..

[29] Khalid Z., Shafqat S.S., Ahmad H.A., Rehman H.M., Munawar M.A., Ahmad M., Asiri A.M., Ashraf M. (2022). Synthesis of 1,2,3-Benzotriazin-4(3H)-one derivatives as α-glucosidase inhibitor and their in-silico study. Med. Chem. Res..

[30] Ekins S., Mestres J., Testa B. (2007). In silico pharmacology for drug discovery: Methods for virtual ligand screening and profiling. Br. J. Pharmacol..

[31] Batool M., Tajammal A., Farhat F., Verpoort F., Khattak Z.A., Shahid M., Ahmad H.A., Munawar M.A., Zia-ur-Rehman M., Asim Raza Basra M. (2018). Molecular docking, computational, and antithrombotic studies of novel 1,3,4-oxadiazole derivatives. Int. J. Mol. Sci..

[32] Kazachenko A.S., Tomilin F.N., Pozdnyakova A.A., Vasilyeva N.Y., Malyar Y.N., Kuznetsova S.A., Avramov P.V. (2020). Theoretical DFT interpretation of infrared spectra of biologically active arabinogalactan sulphated derivatives. Chem. Pap..

[33] Reyes-Chaparro A., Flores-Lopez N., Quintanilla-Guerrero F., Nicolás-Álvarez D.E., Hernandez-Martinez A. (2023). Design of new reversible and selective inhibitors of monoamine oxidase A and a comparison with drugs already approved. Bull. Natl. Res. Cent..

[34] Sayed D.S.E., Abdelrehim E.-S.M. (2022). Spectroscopic details on the molecular structure of pyrimidine-2-thiones heterocyclic compounds: Computational and antiviral activity against the main protease enzyme of SARS-CoV-2. BMC Chem..

[35] Channar P.A., Arshad N., Larik F.A., Farooqi S.I., Saeed A., Hökelek T., Batool B., Ujan R., Ali H.S., Flörke U. (2019). 4-(4-Bromophenyl)thiazol-2-amine: Crystal structure determination, DFT calculations, visualizing intermolecular interactions using Hirshfeld surface analysis, and DNA binding studies. J. Phys. Org. Chem..

[36] Khan S., Ullah H., Taha M., Rahim F., Sarfraz M., Iqbal R., Iqbal N., Hussain R., Ali Shah S.A., Ayub K. (2023). Synthesis, DFT Studies, Molecular Docking and Biological Activity Evaluation of Thiazole-Sulfonamide Derivatives as Potent Alzheimer’s Inhibitors. Molecules.

[37] Elkolli M., Chafai N., Chafaa S., Kadi I., Bensouici C., Hellal A.J. (2022). New phosphinic and phosphonic acids: Synthesis, antidiabetic, anti-Alzheimer, antioxidant activity, DFT study and SARS-CoV-2 inhibition. J. Mol. Struct..

[38] Kanaani A., Ajloo D., Kiyani H., Ghasemian H., Vakili M., Feizabadi M. (2016). Molecular structure, spectroscopic investigations and computational study on the potential molecular switch of (E)-1-(4-(2-hydroxybenzylideneamino)phenyl)ethanone. Mol. Phys..

[39] Kosar B., Albayrak C., Spectroscopy B. (2011). Spectroscopic investigations and quantum chemical computational study of (E)-4-methoxy-2-[(p-tolylimino)methyl]phenol. Spectrochim. Acta A Mol. Biomol..

[40] Akram N., Mansha A., Premkumar R., Franklin Benial A.M., Asim S., Iqbal S.Z., Ali H.S. (2020). Spectroscopic, quantum chemical and molecular docking studies on 2,4-dimethoxy-1,3,5-triazine: A potent inhibitor of protein kinase CK2 for the development of breast cancer drug. Mol. Simul..

[41] Chaudhry F., Naureen S., Ashraf M., Al-Rashida M., Jahan B., Munawar M.A., Khan M.A. (2019). Imidazole-pyrazole hybrids: Synthesis, characterization and in-vitro bioevaluation against α-glucosidase enzyme with molecular docking studies. Bioorg. Chem..

[42] Becke A.D. (1993). Density-functional thermochemistry. III. The role of exact exchange. J. Chem. Phys..

[43] Lee C., Yang W., Parr R.G. (1988). Development of the Colle-Salvetti correlation-energy formula into a functional of the electron density. Phys. Rev. B.

[44] Miehlich B., Savin A., Stoll H., Preuss H. (1989). Results obtained with the correlation energy density functionals of becke and Lee, Yang and Parr. Chem. Phys. Lett..

