# Supplementary material for: A Combined Experimental and Computational Study of Novel Benzotriazinone Carboxamides as Alpha-Glucosidase Inhibitors
Source: Molecules. 2023 Sep 14;28(18):6623. doi: 10.3390/molecules28186623 (PMC10535199; doi:10.3390/molecules28186623)
Supplement: Supplementary file 1 [file molecules-28-06623-s001.zip › molecules-2448831-supplementary.pdf]

A Combined Experimental and Computational Study of Novel Benzotriazinone Carboxamides as Alpha-Glucosidase Inhibitors

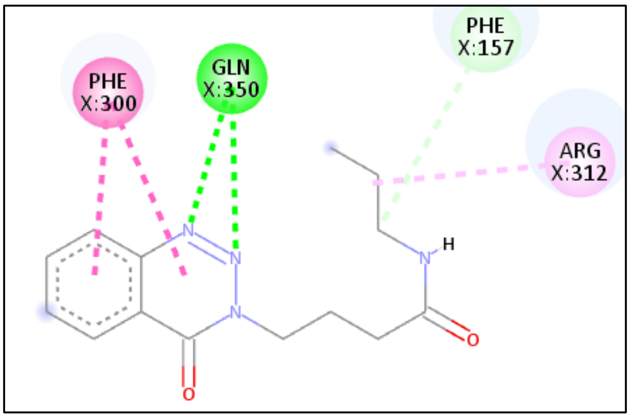

14a

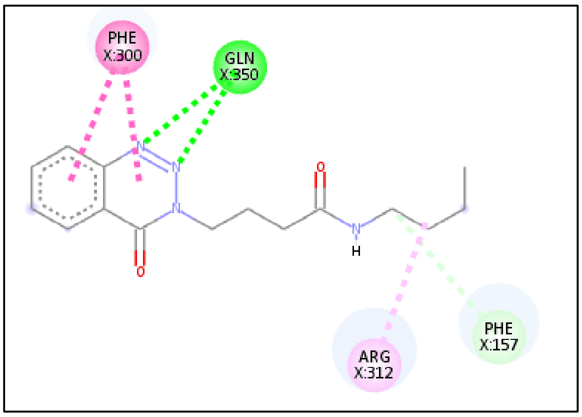

14b

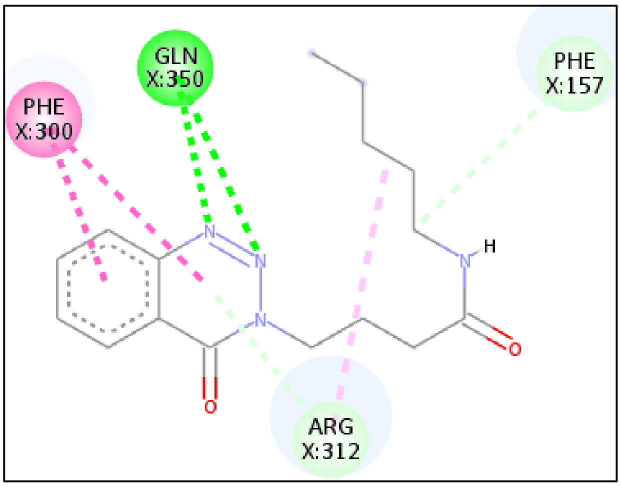

14c

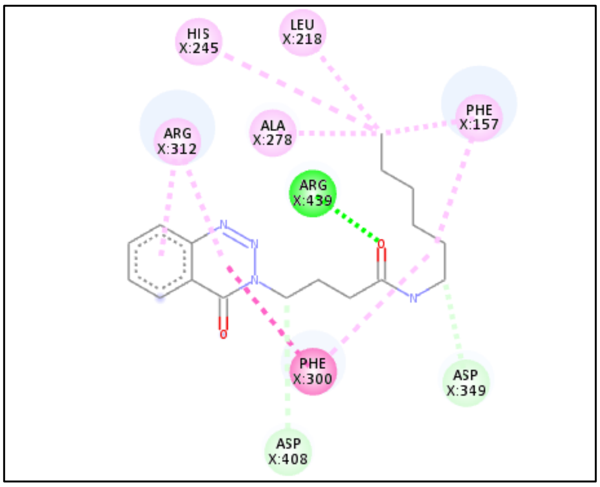

14d

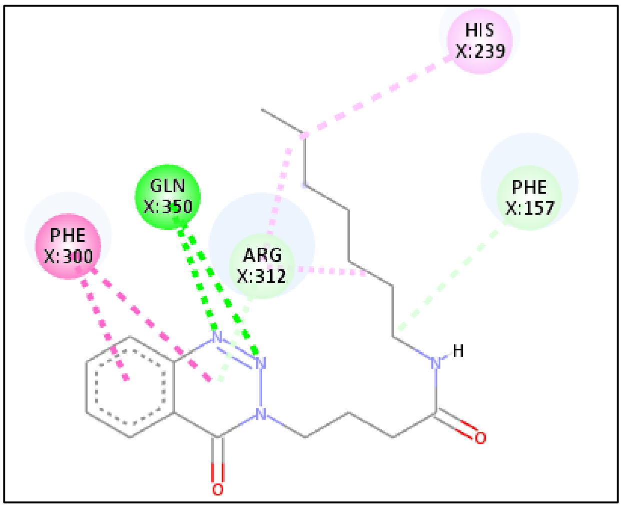

14e

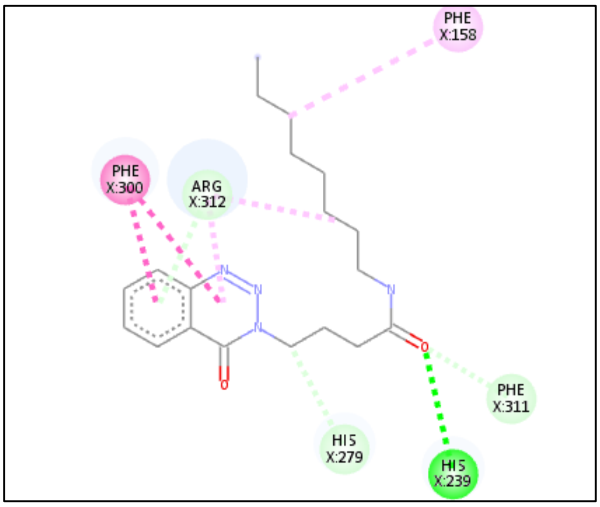

14f

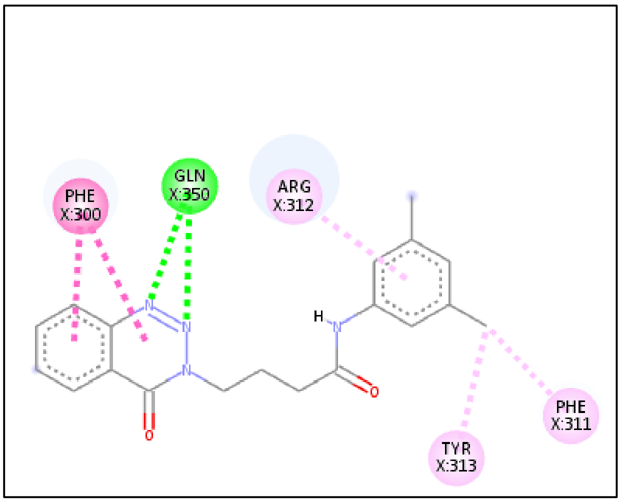

14g

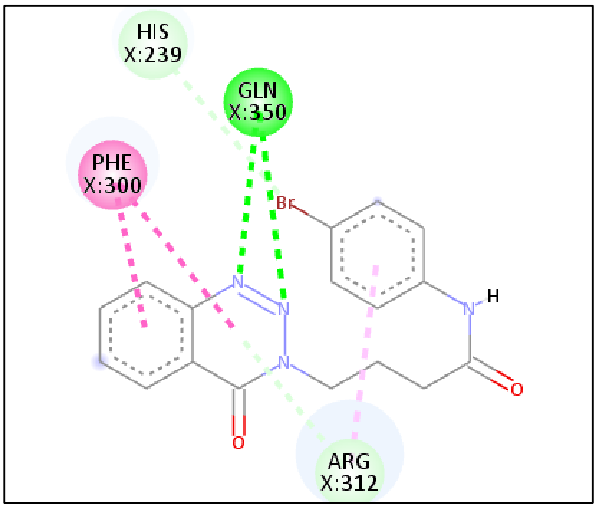

14h

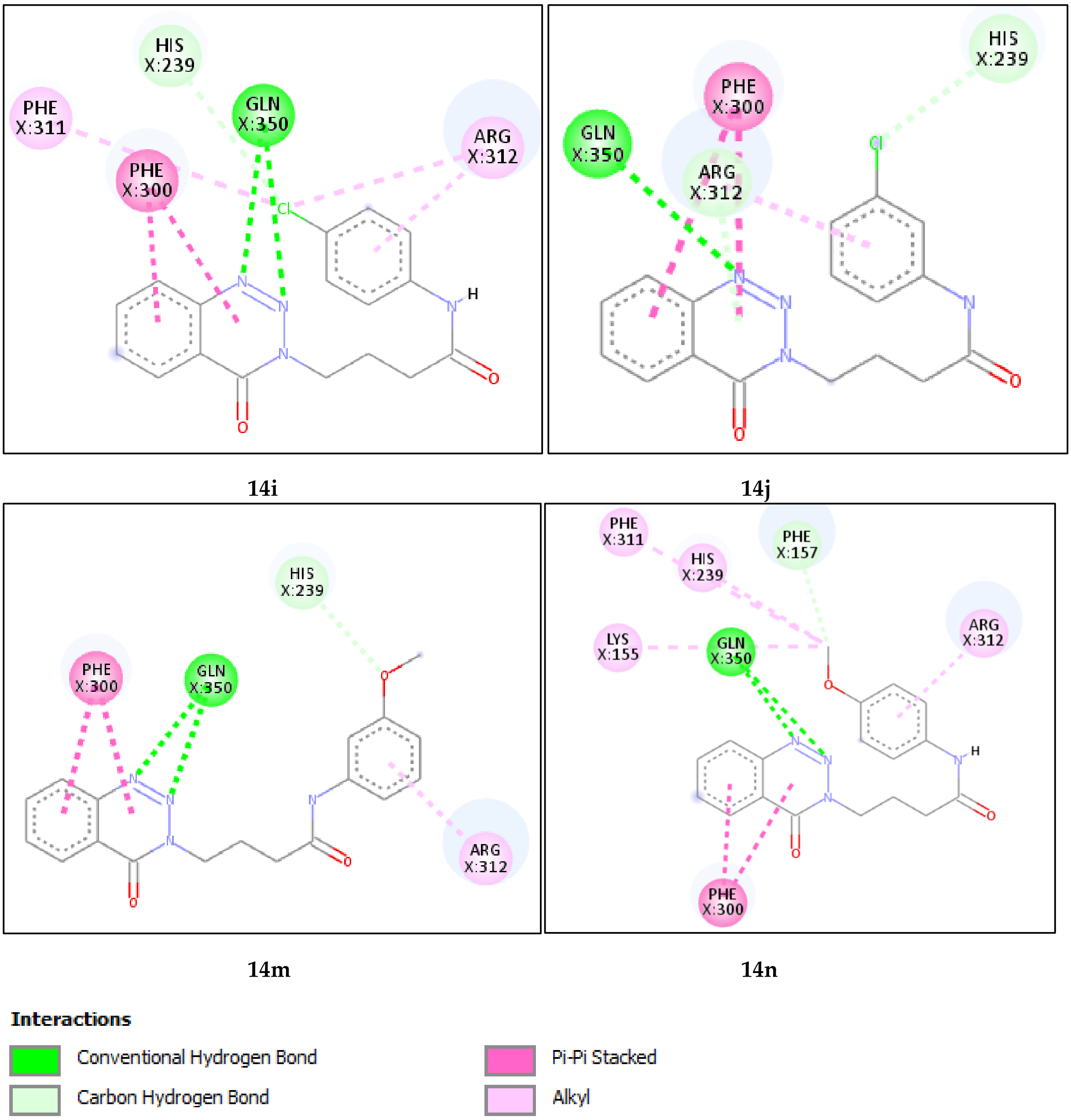

Figure S1. 2D Binding-interaction diagrams of all synthesized compounds with alpha-glucosidase

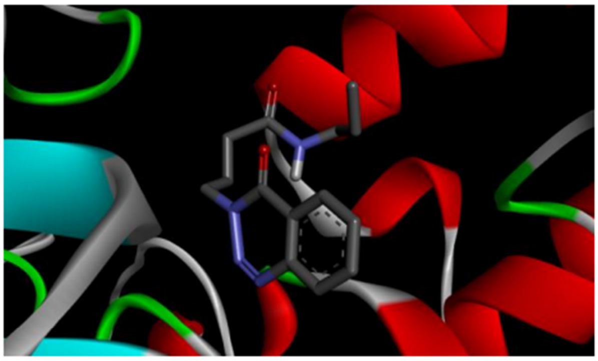

14a

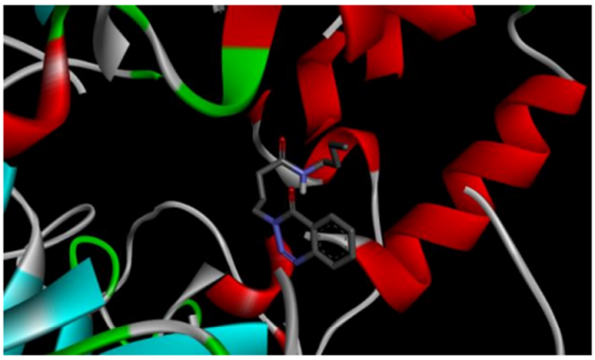

14b

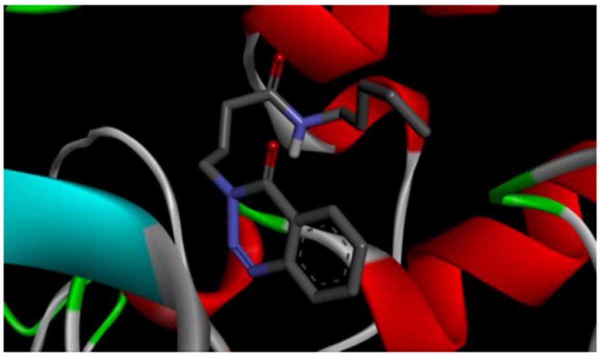

14c

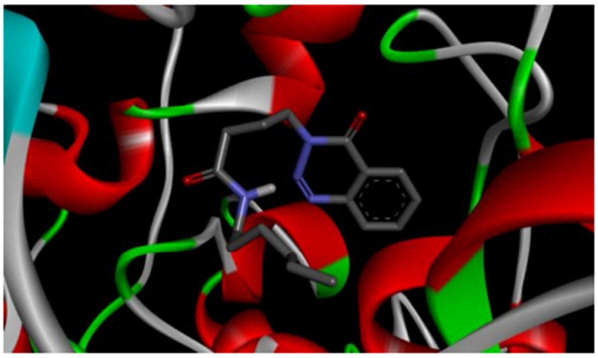

14d

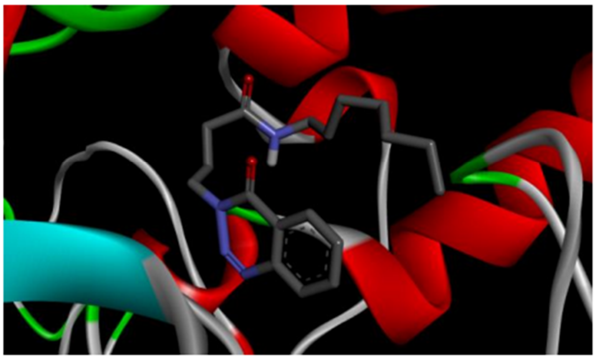

14e

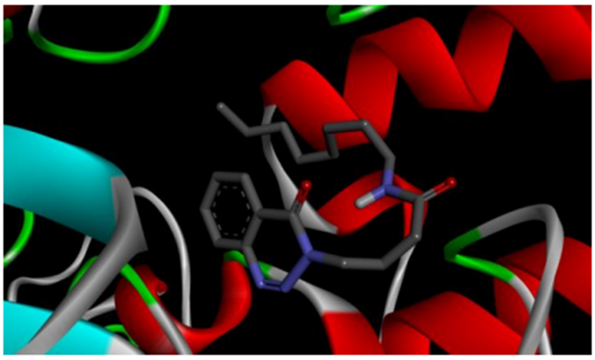

14f

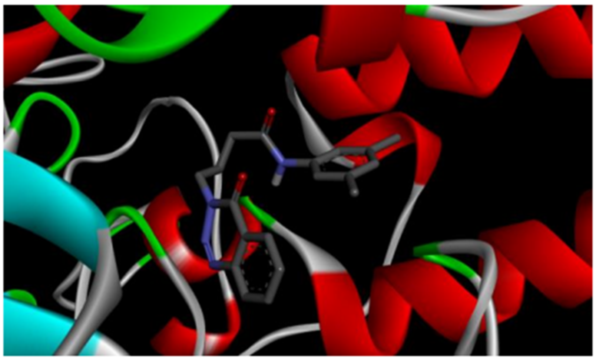

14g

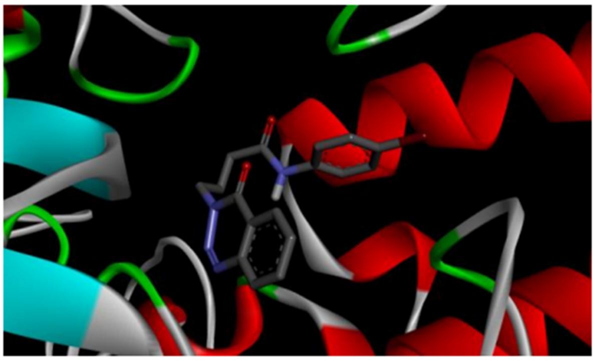

14h

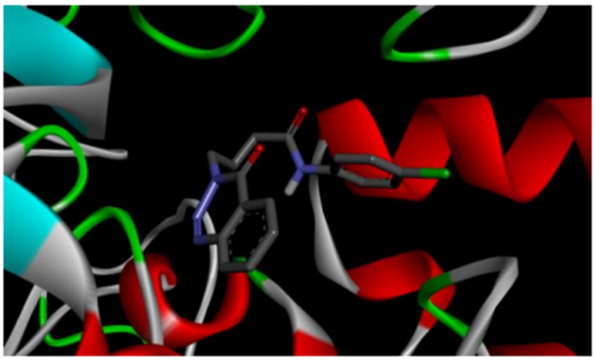

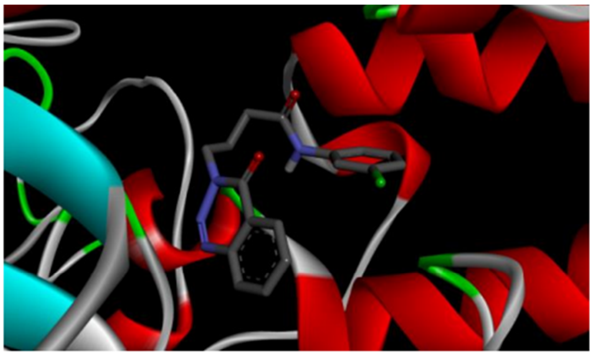

14i **14j**

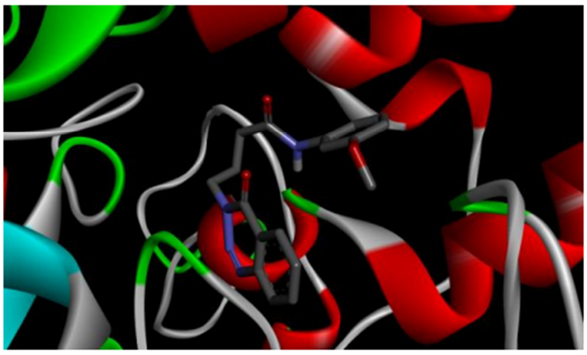

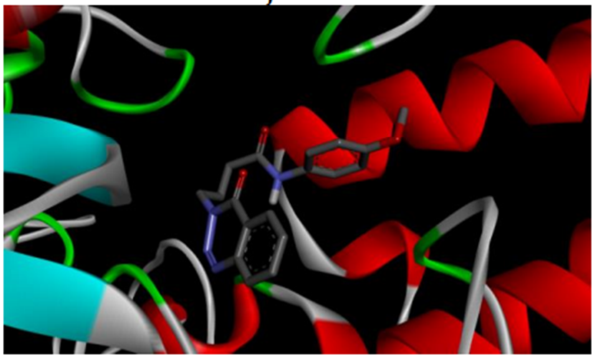

14m 14n

Figure S2. 3D Binding-interaction diagrams of all synthesized compounds within alpha-glucosidase

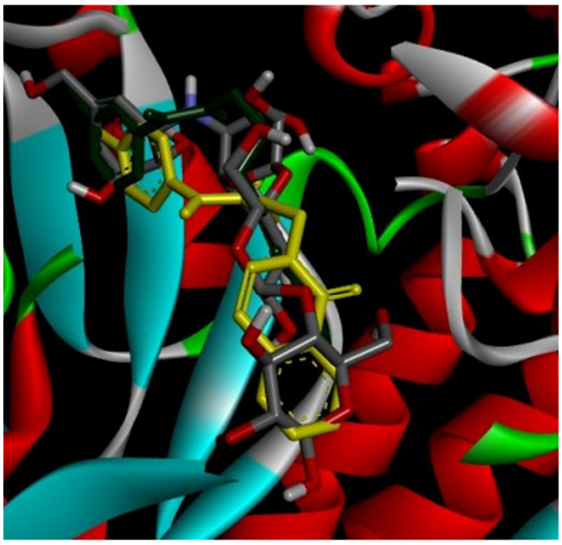

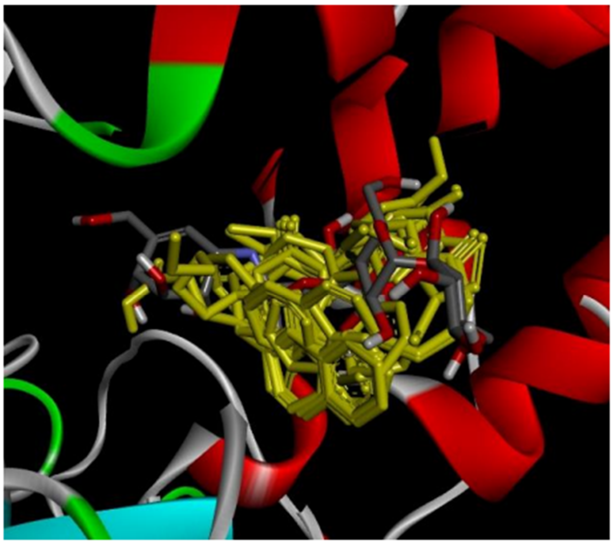

A **B**

Figure S3. Molecular docking result of reference drug acarbose (A) and all synthesized derivatives (B)

Table S1. Docking scores of all synthesized compounds

Compound	Free binding energy (kcal mol^{-1})
14a	-7.5
14b	-7.8
14c	-7.5
14d	-8.0
14e	-7.6
14f	-7.4
14g	-8.8
14h	-8.7
14i	-9.1
14j	-9.0
14k	-9.2
14l	-9.2
14m	-9.0
14n	-9.1
acarbose	-9.0

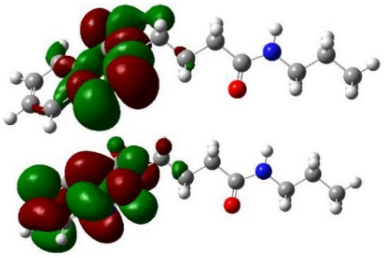

14a

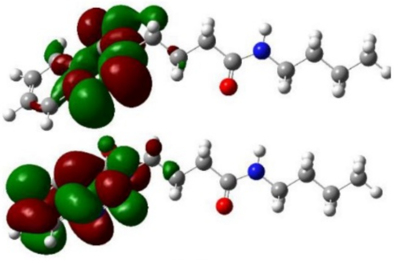

14b

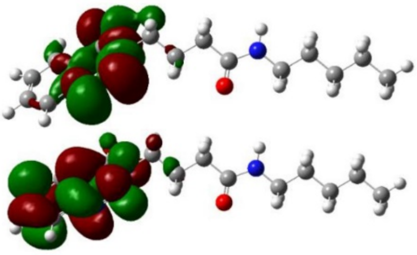

14c

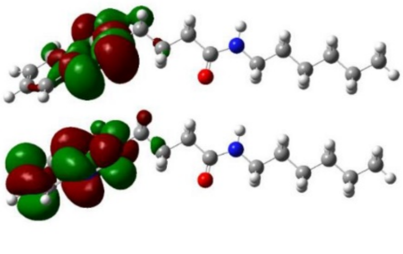

14d

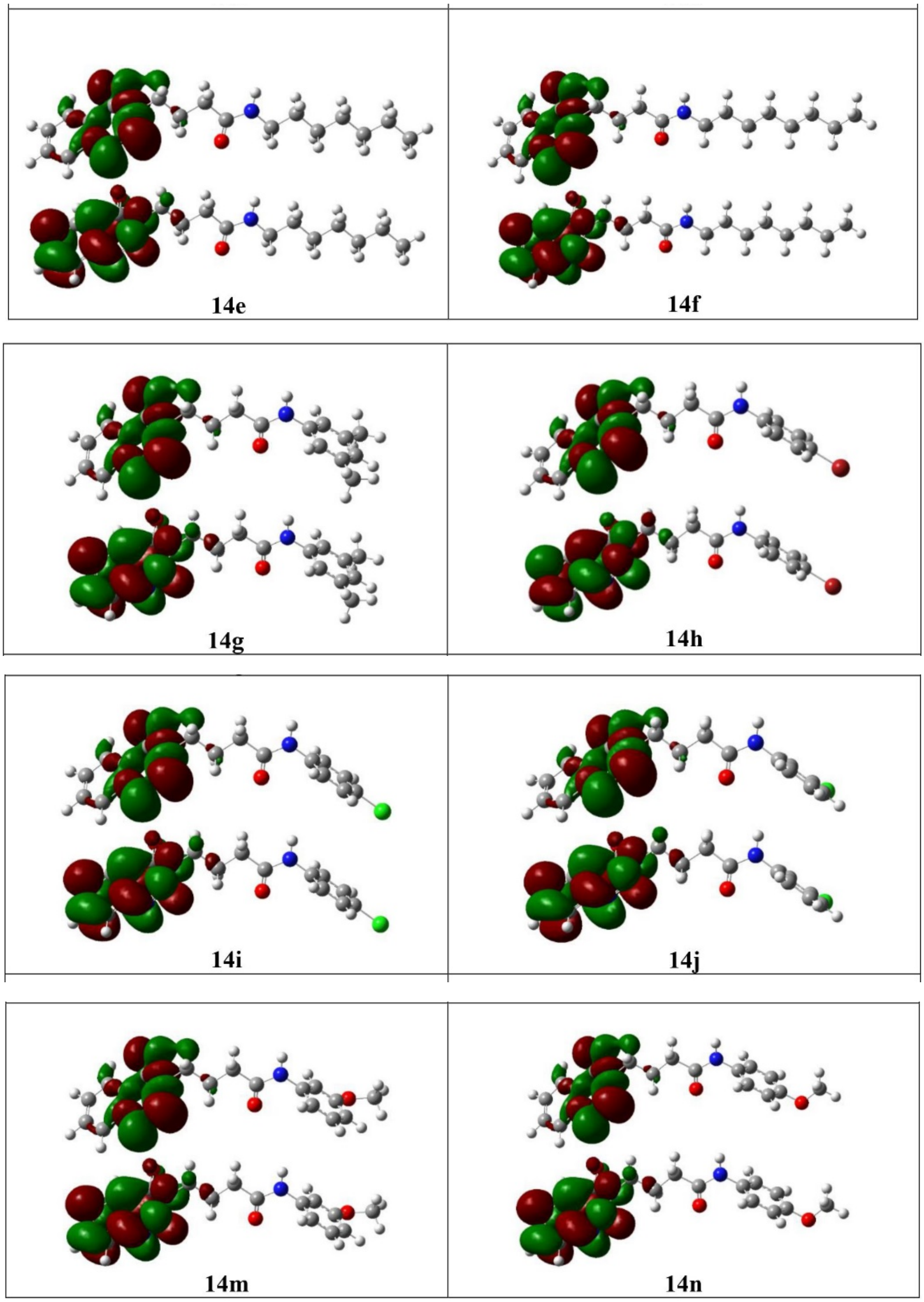

Figure S4. Pictorial illustration of charge density distribution in molecular orbitals of **14a-14n**. HOMO is shown at the bottom and LUMO is at the top.

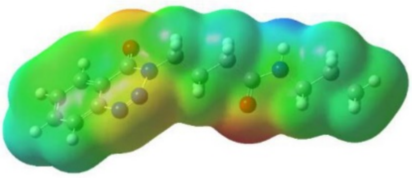 14a	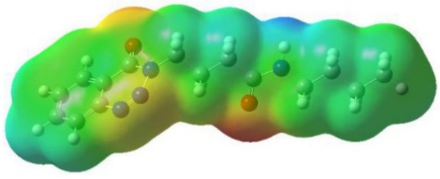 14b
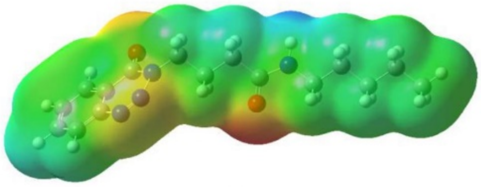 14c	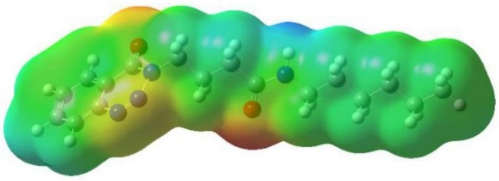 14d
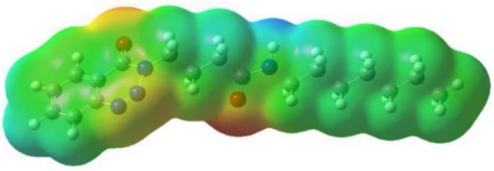 14e	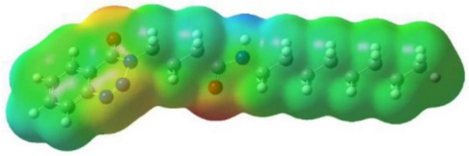 14f
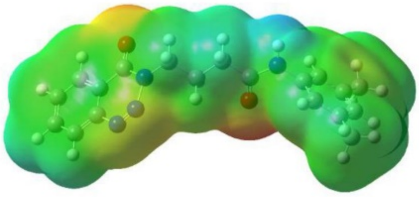 14g	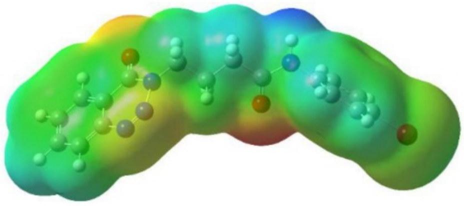 14h
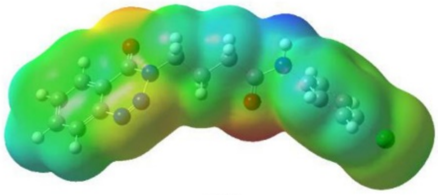 14i	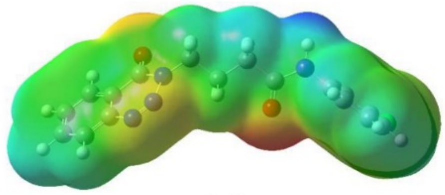 14j

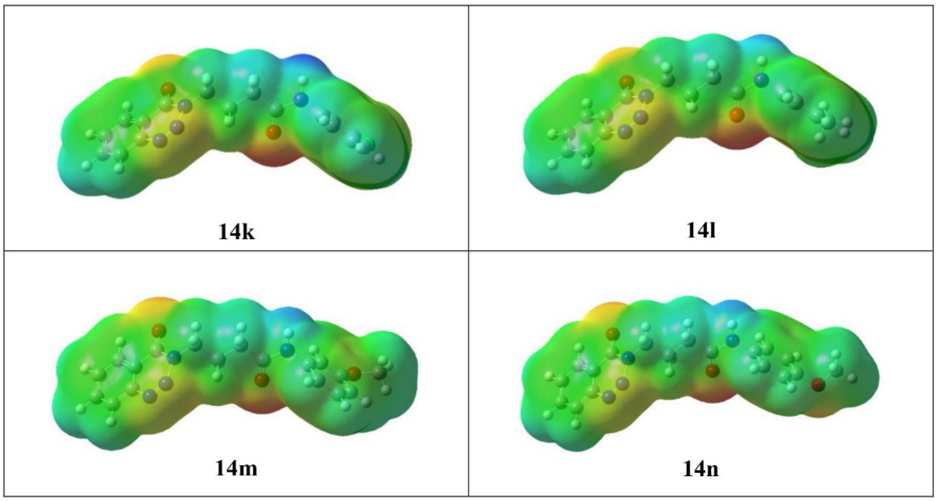

Figure S5. Molecular electrostatic potential mapping on the surface of **14a-14n**

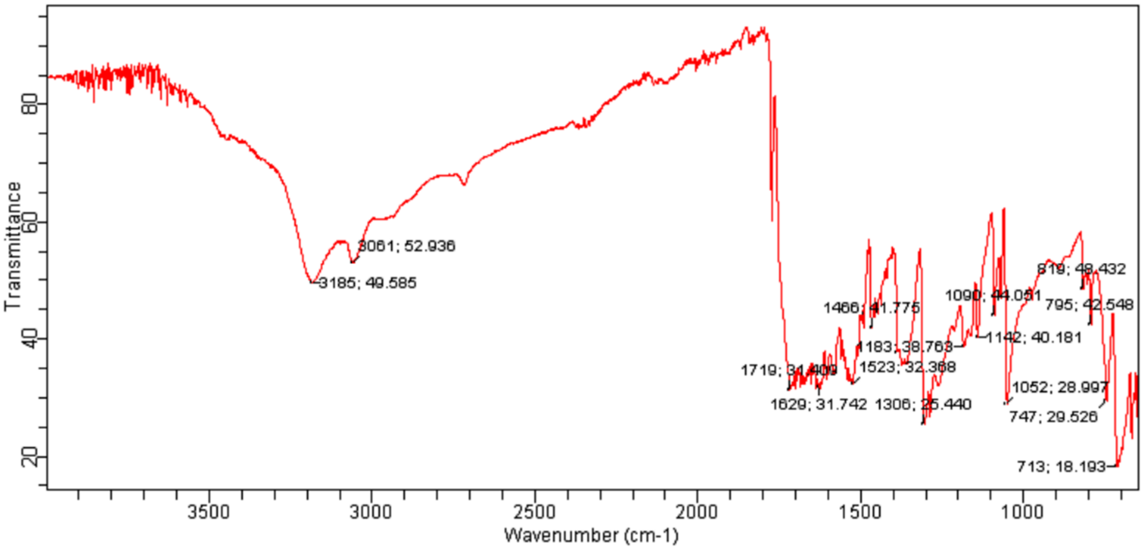

Figure S6. [1]**H-NMR of 12**

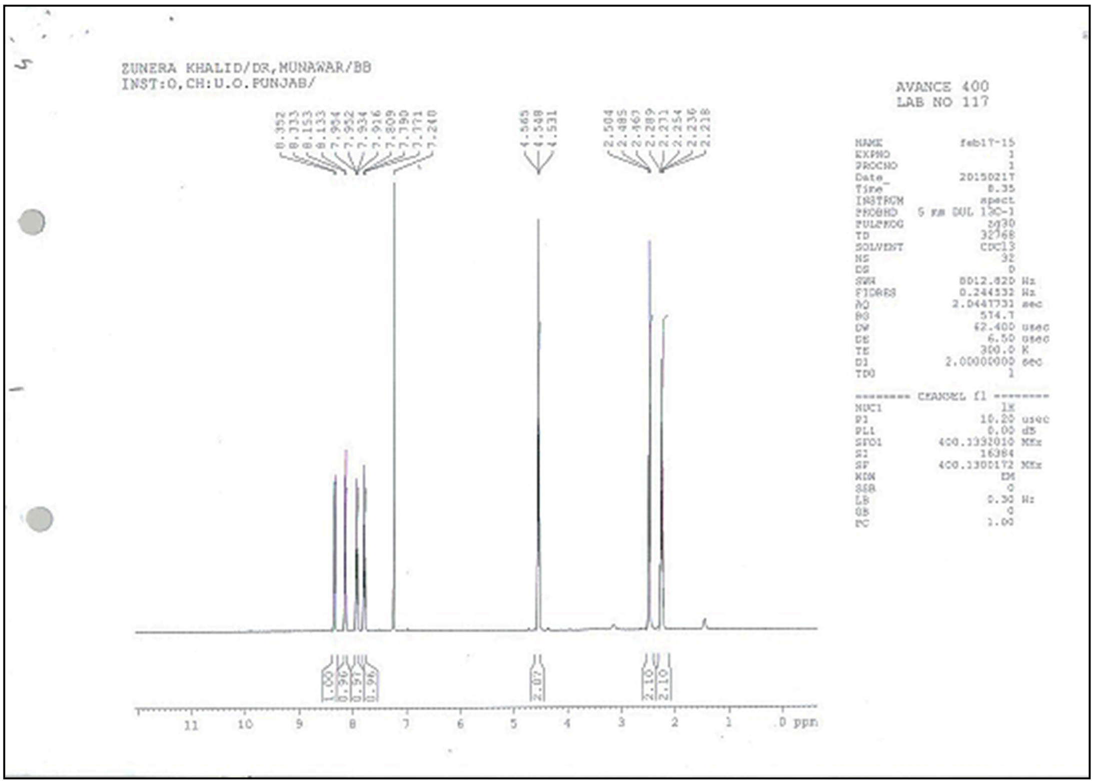

Figure S7. ^{1}H-NMR of 12

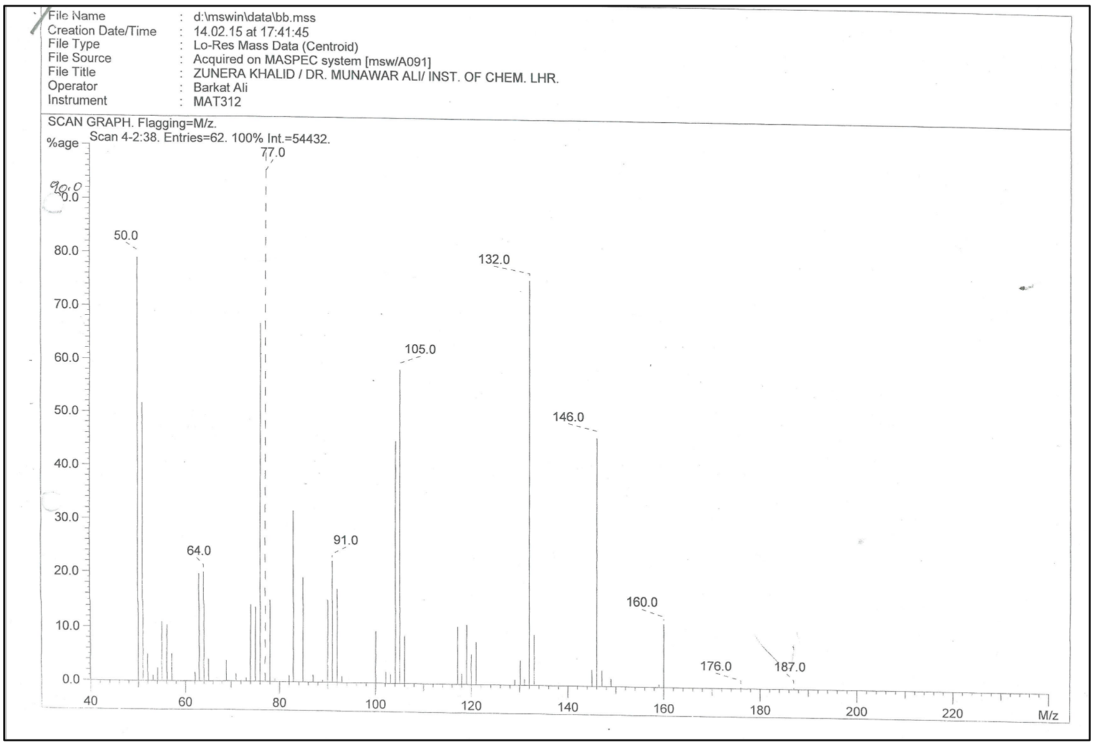

Figure S8. EIMS of 12

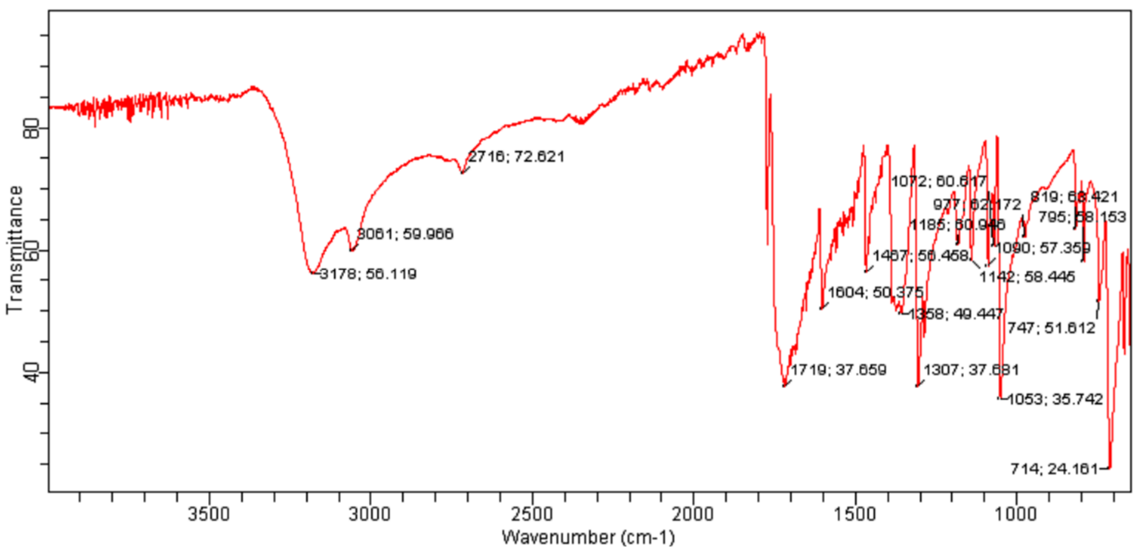

Figure S9. FT-IR of 13

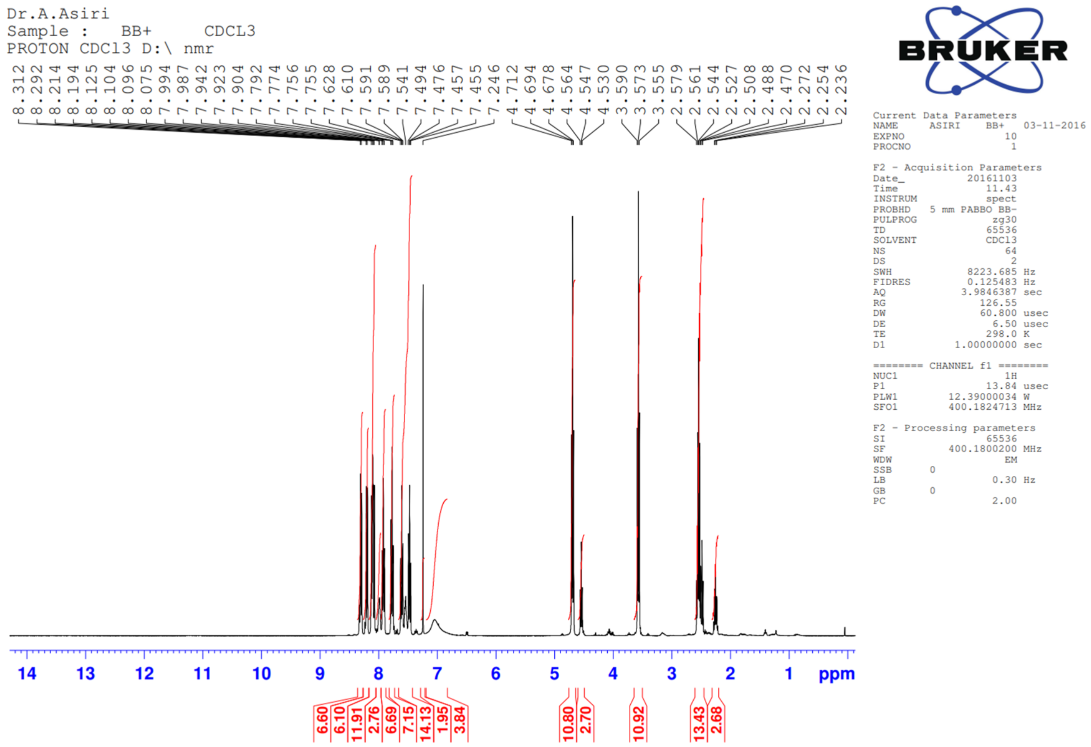

Figure S10. ^{1}H-NMR of 13

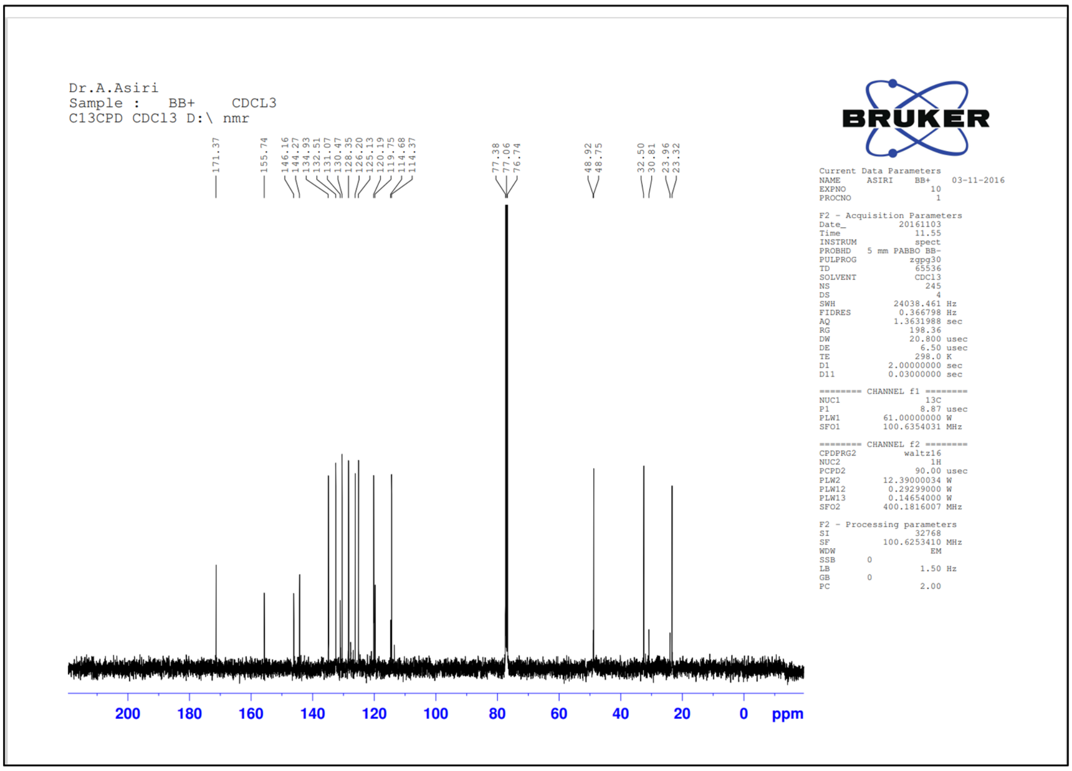

Figure S11. ^{13}**C-NMR of 13**

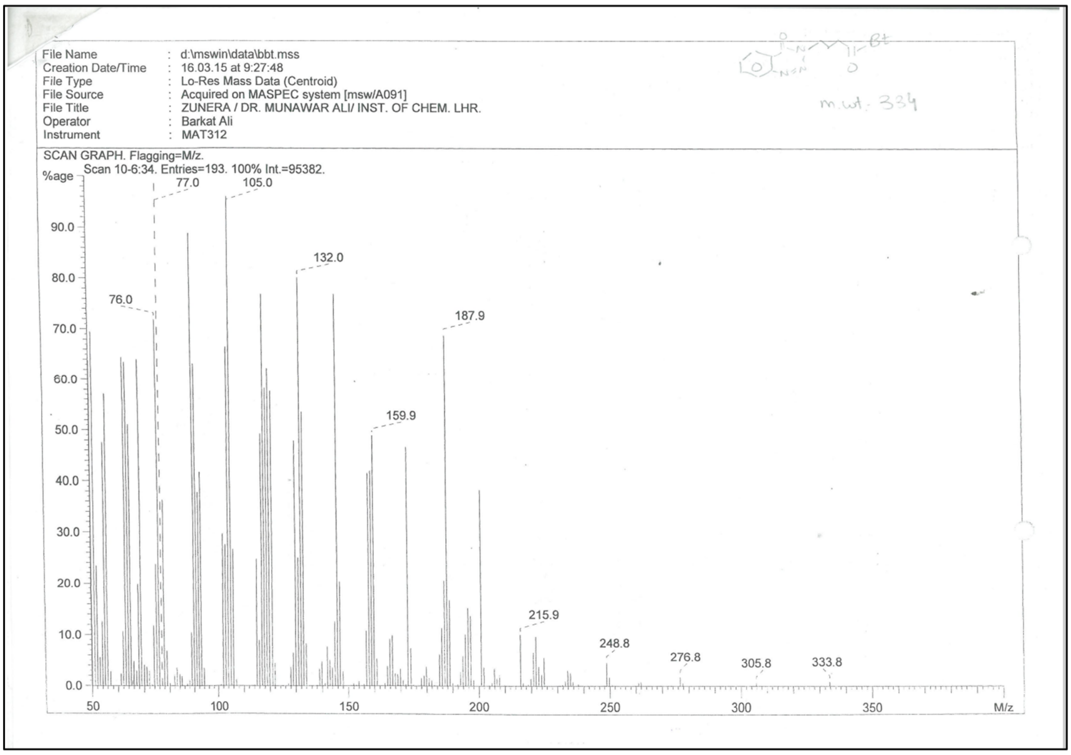

Figure S12. EIMS of 13

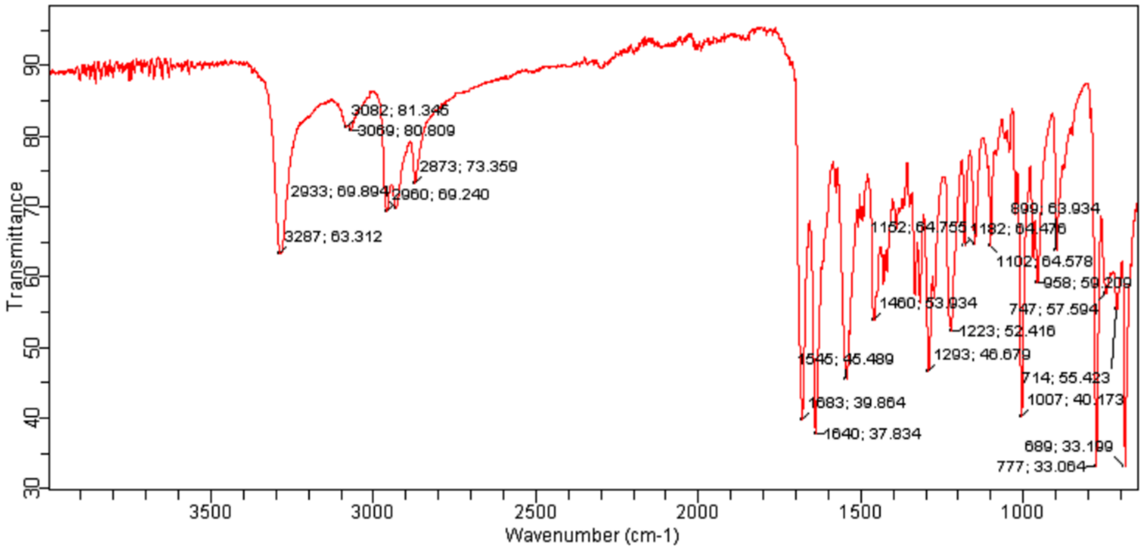

Figure S13. FTIR of 14a

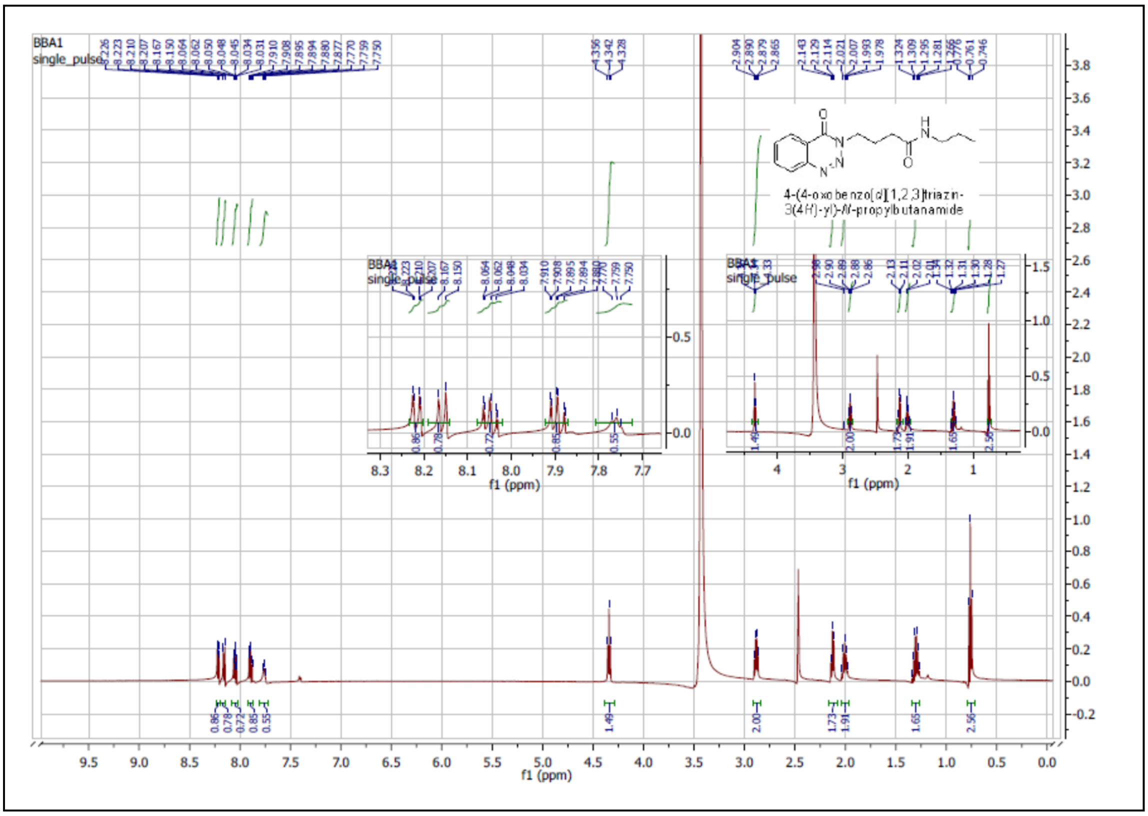

Figure S14. ^{1}H-NMR of 14a

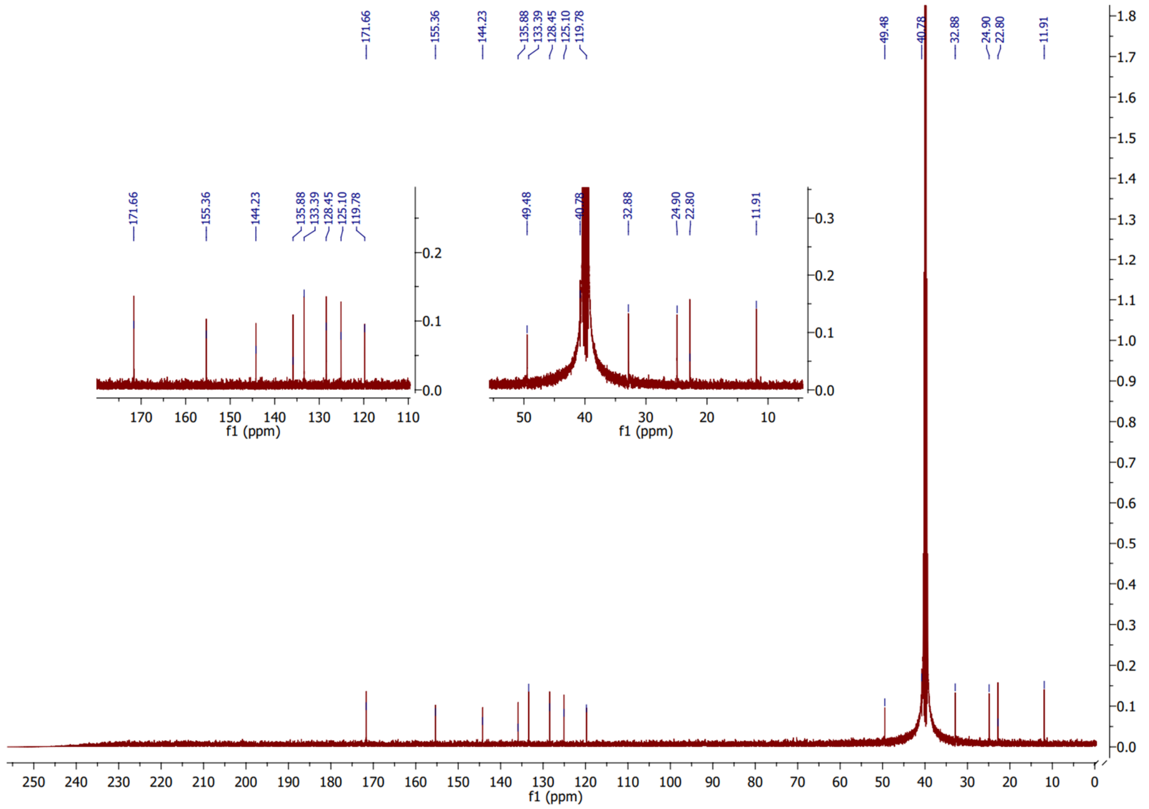

Figure S15. ^{13}**C-NMR of 14a**

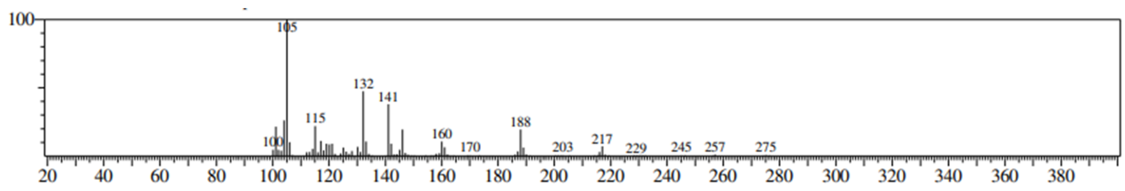

Figure S16. GC-MS of 14a

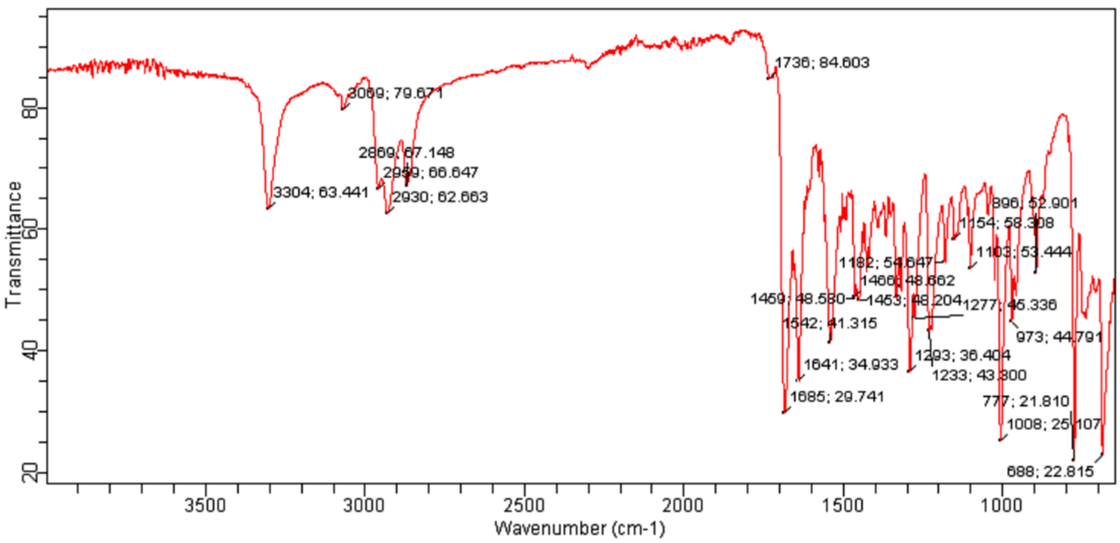

Figure S17. FT-IR of 14b

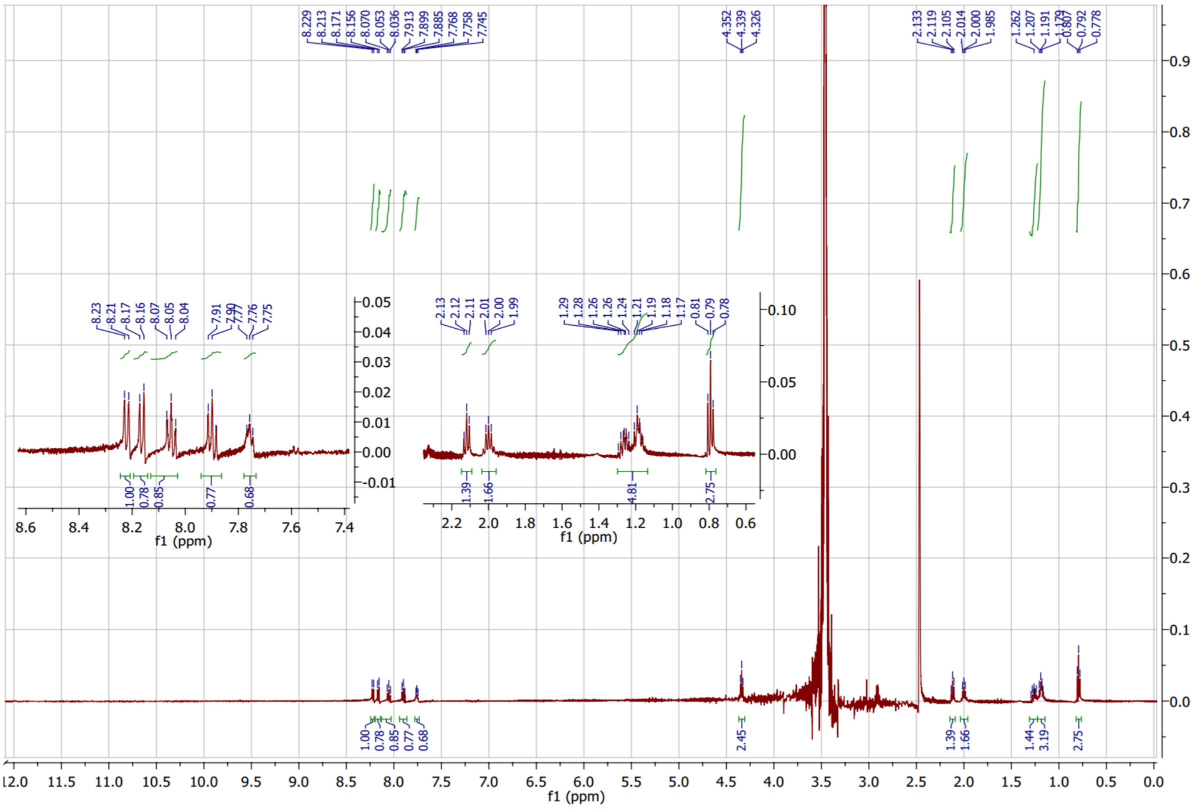

Figure S18. ^{1}H-NMR of 14b

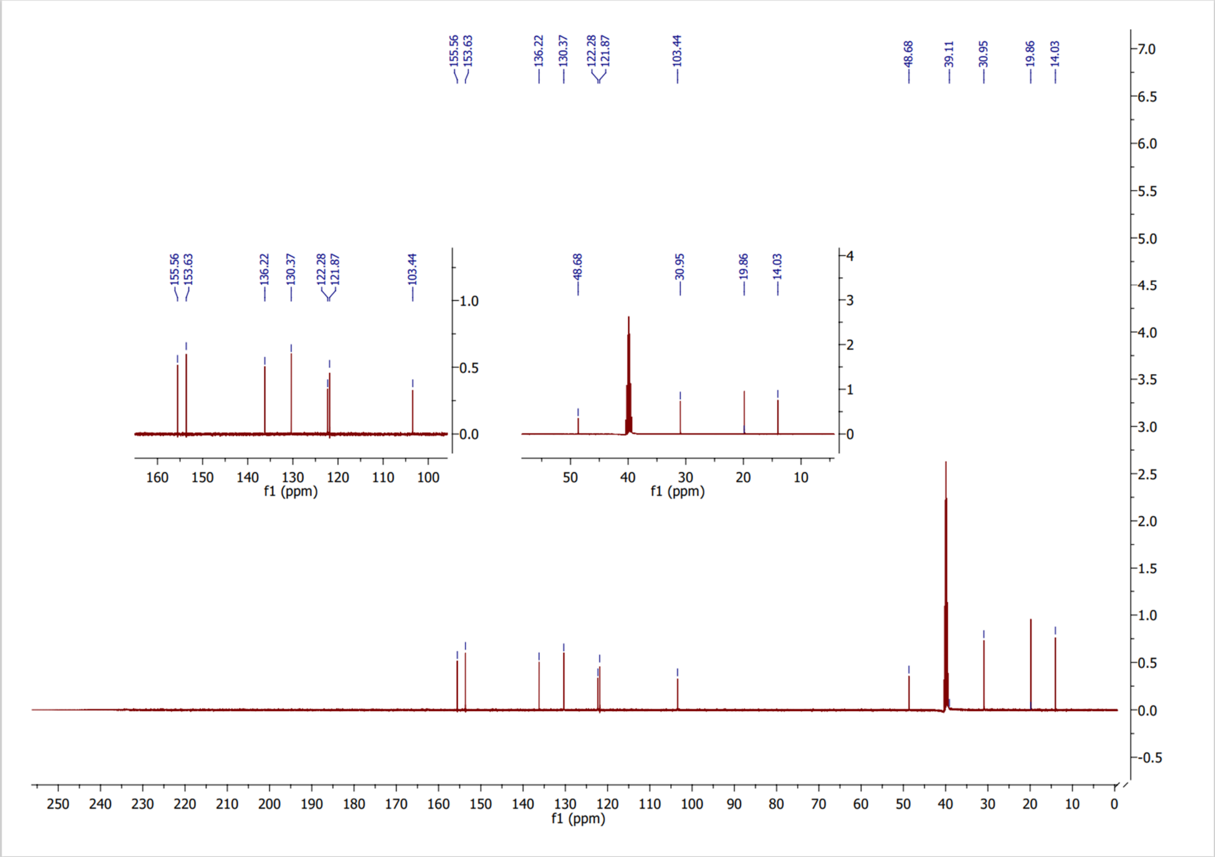

Figure S19. ^{13}C-NMR of 14b

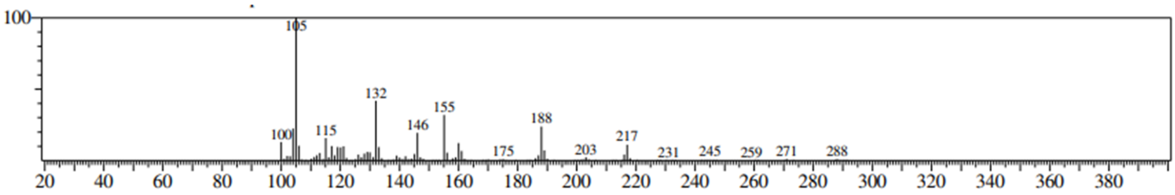

Figure S20. GC-MS of 14b

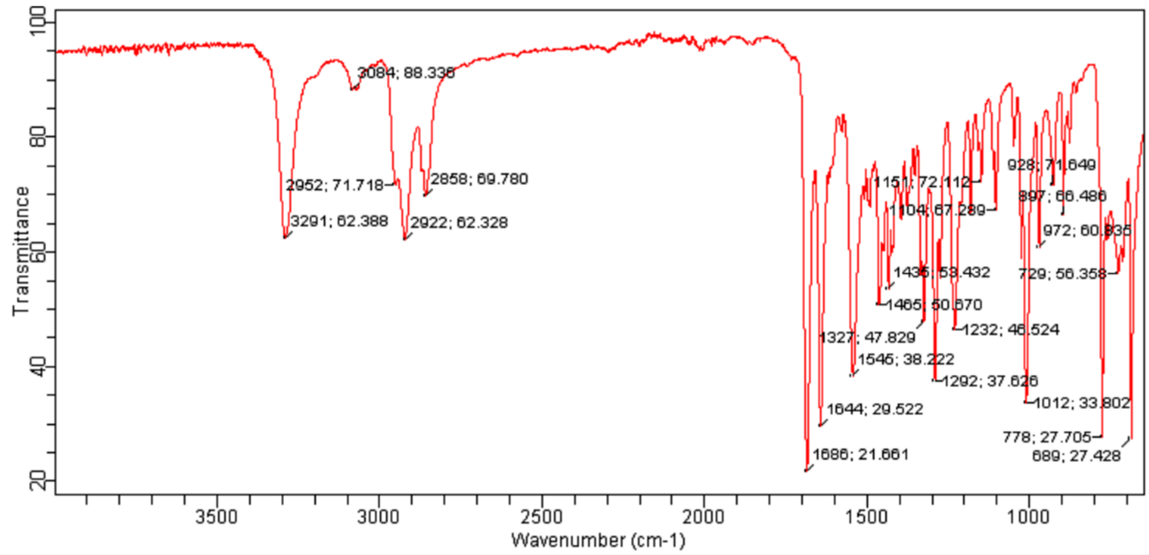

Figure S21. FT-IR of 14c

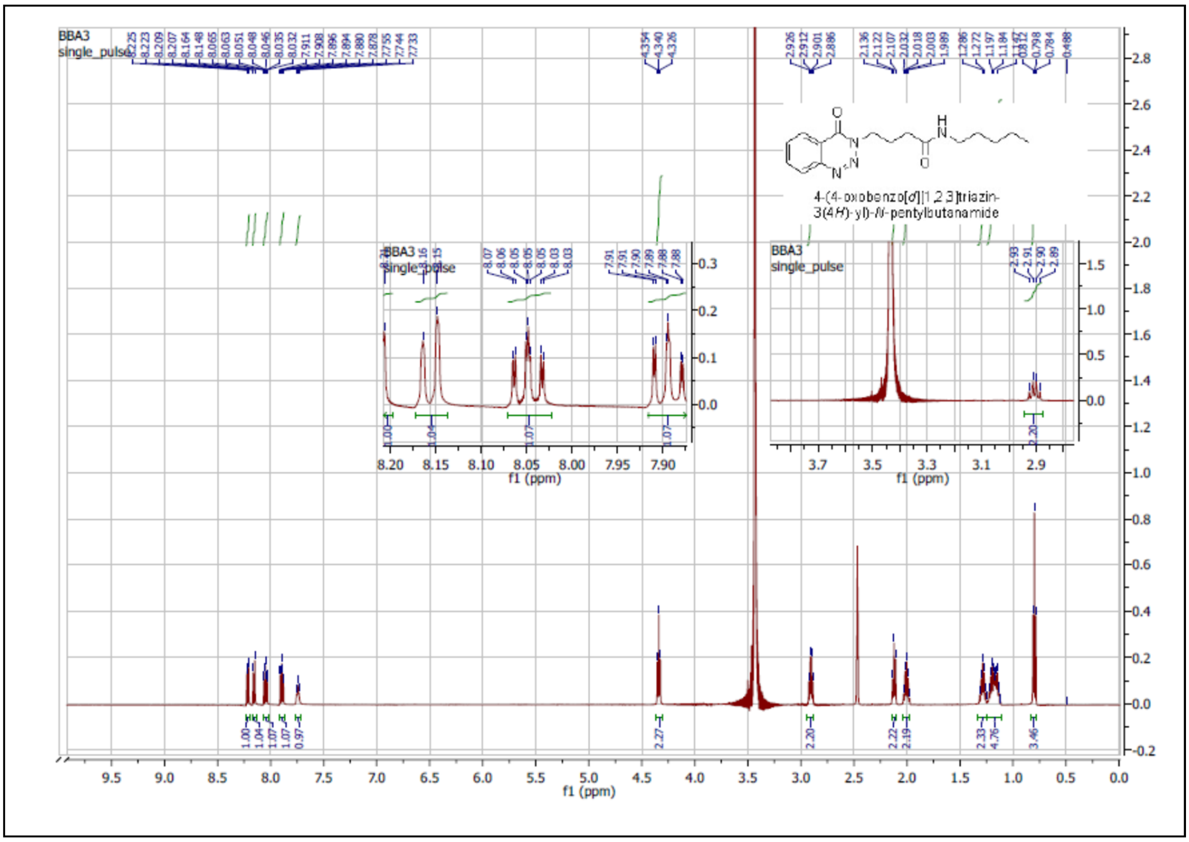

Figure S22. ^{1}H-NMR of 14c

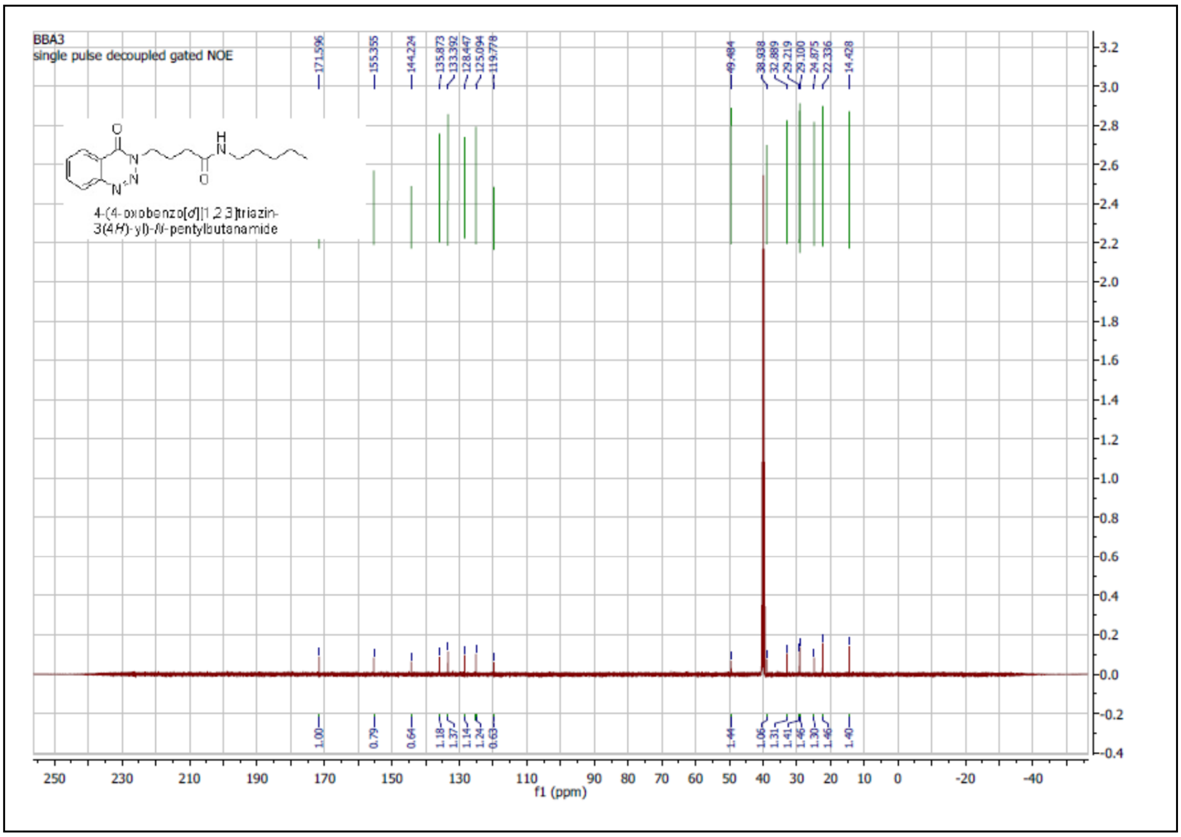

Figure S23. ^{13}C-NMR of 14c

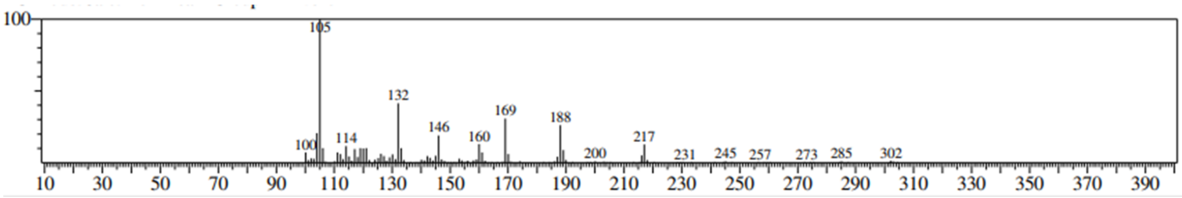

Figure S24. EIMS of 14c

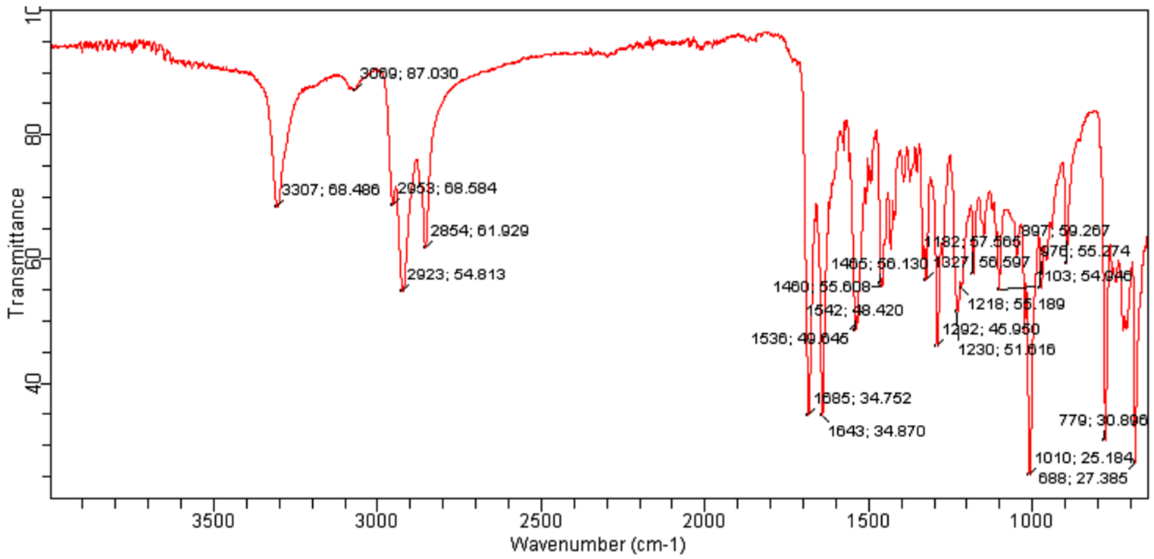

Figure S25. FT-IR of 14d

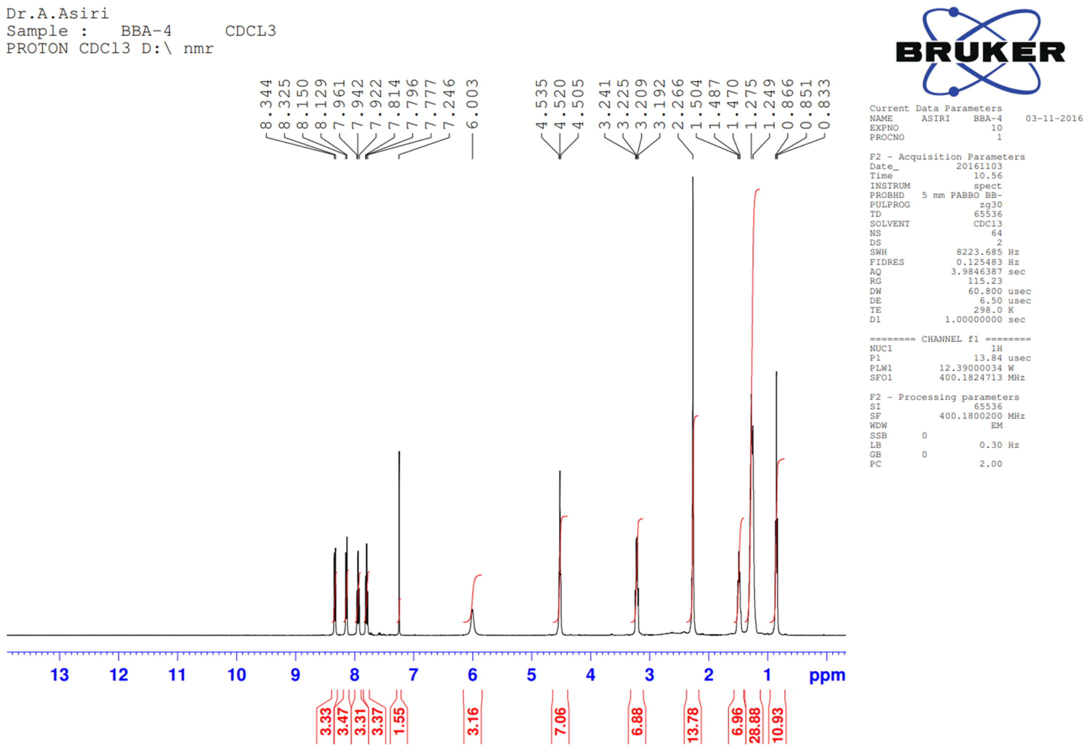

Figure S26. ^{1}H-NMR of 14d

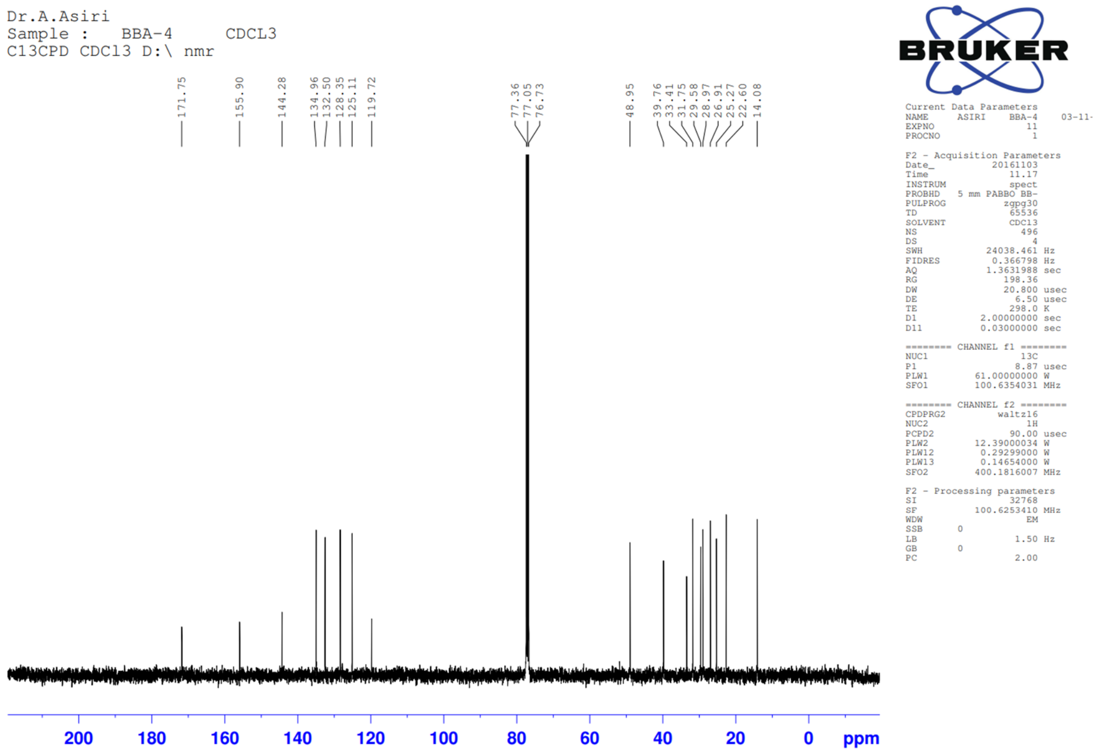

Figure S27. ^{13}C-NMR of 14d

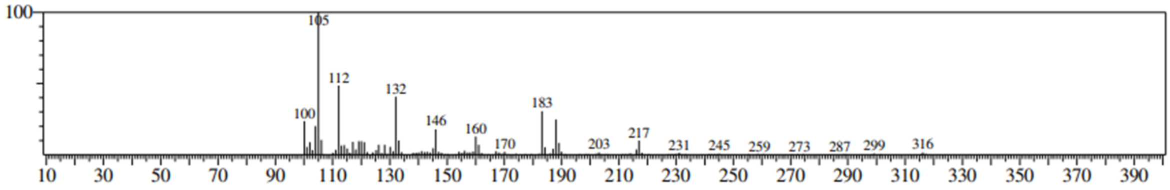

Figure S28. EIMS of 14d

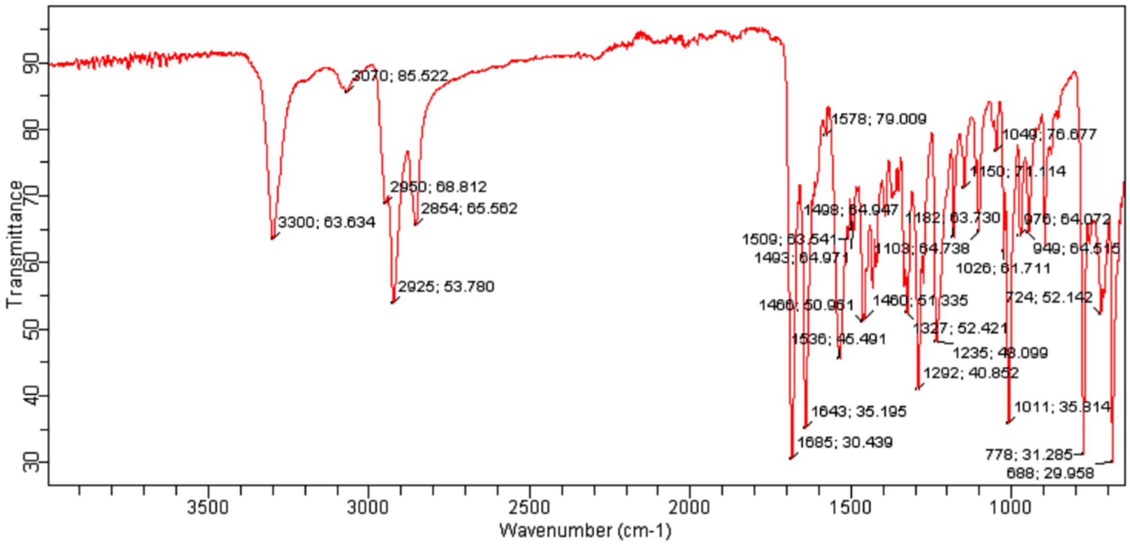

Figure S29. FT-IR of 14e

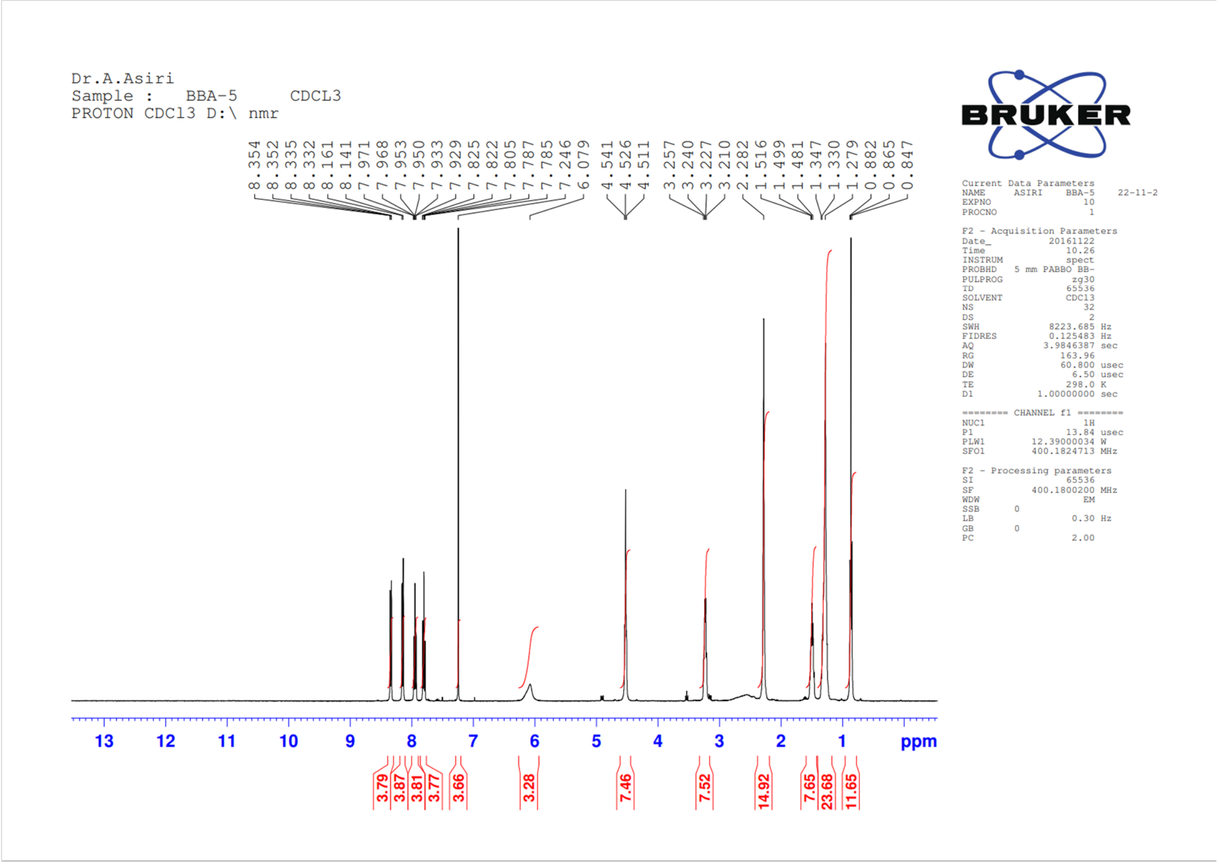

Figure S30. ^{1}H-NMR of 14e

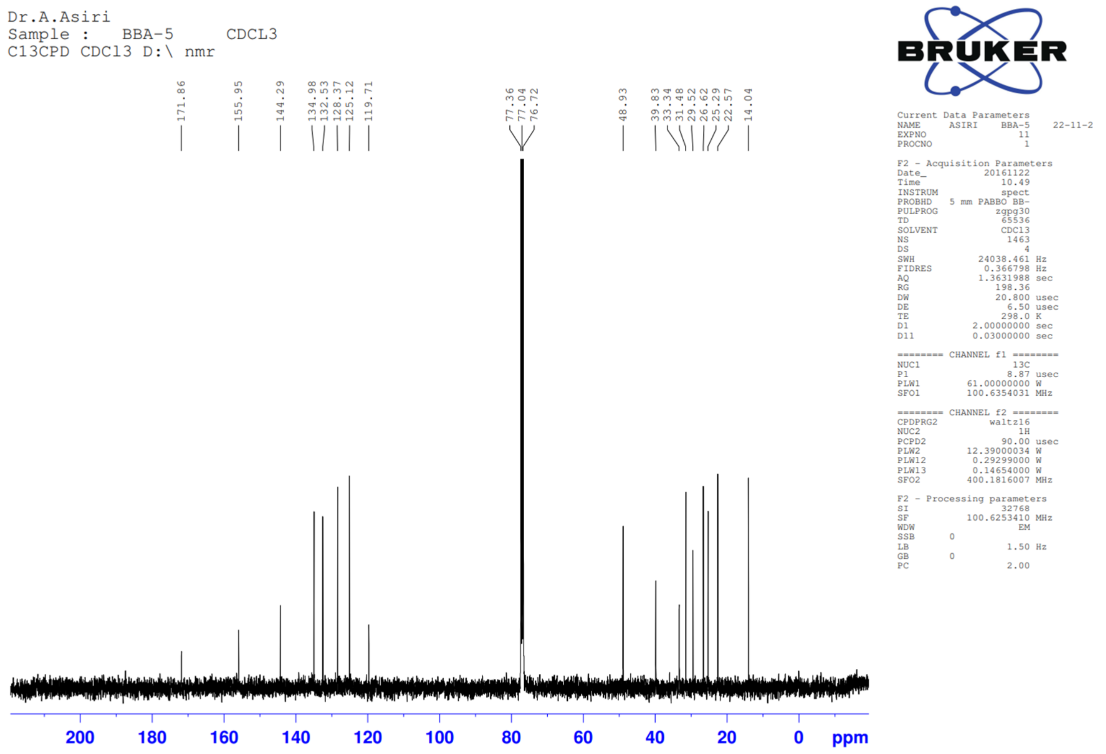

Figure S31. ^{13}**C-NMR of 14e**

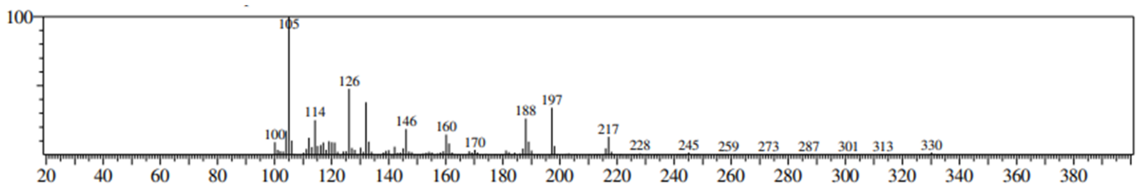

Figure S32. EIMS of 14e

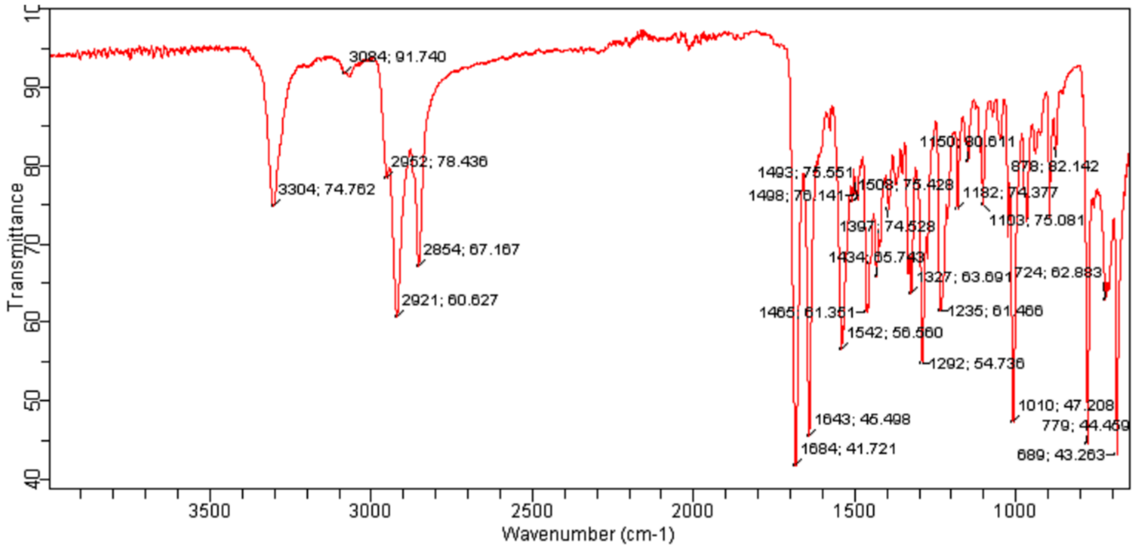

Figure S33. FT-IR of 14f

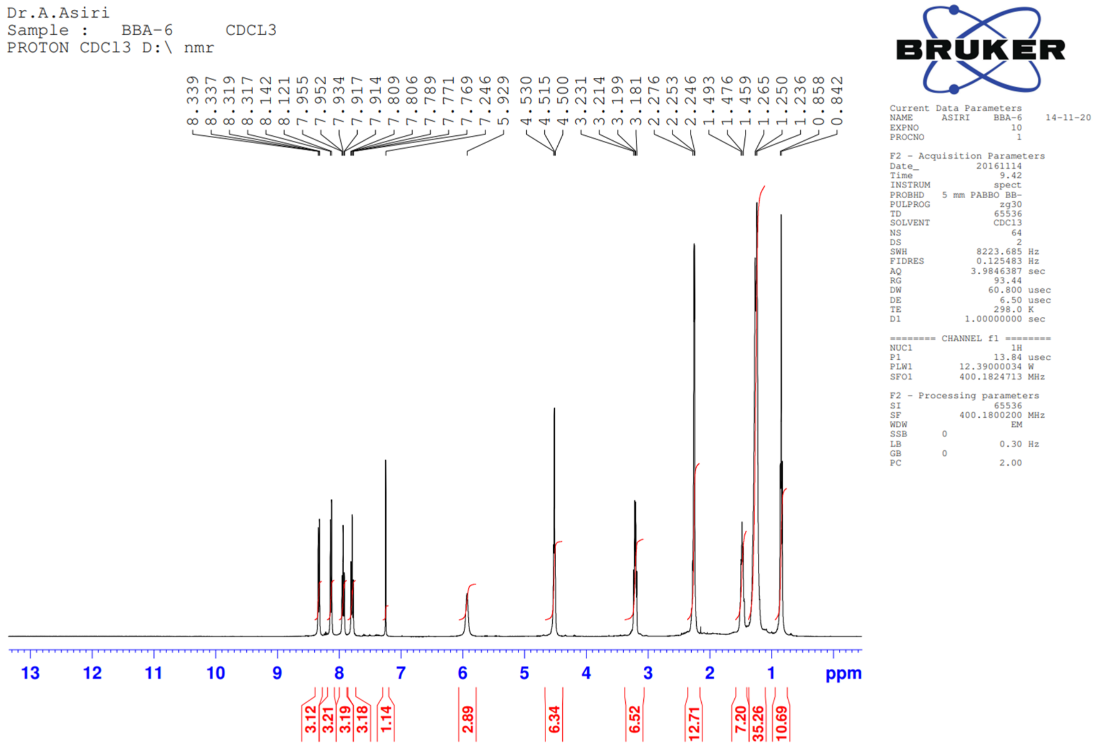

Figure S34. ^{1}H-NMR of 14f

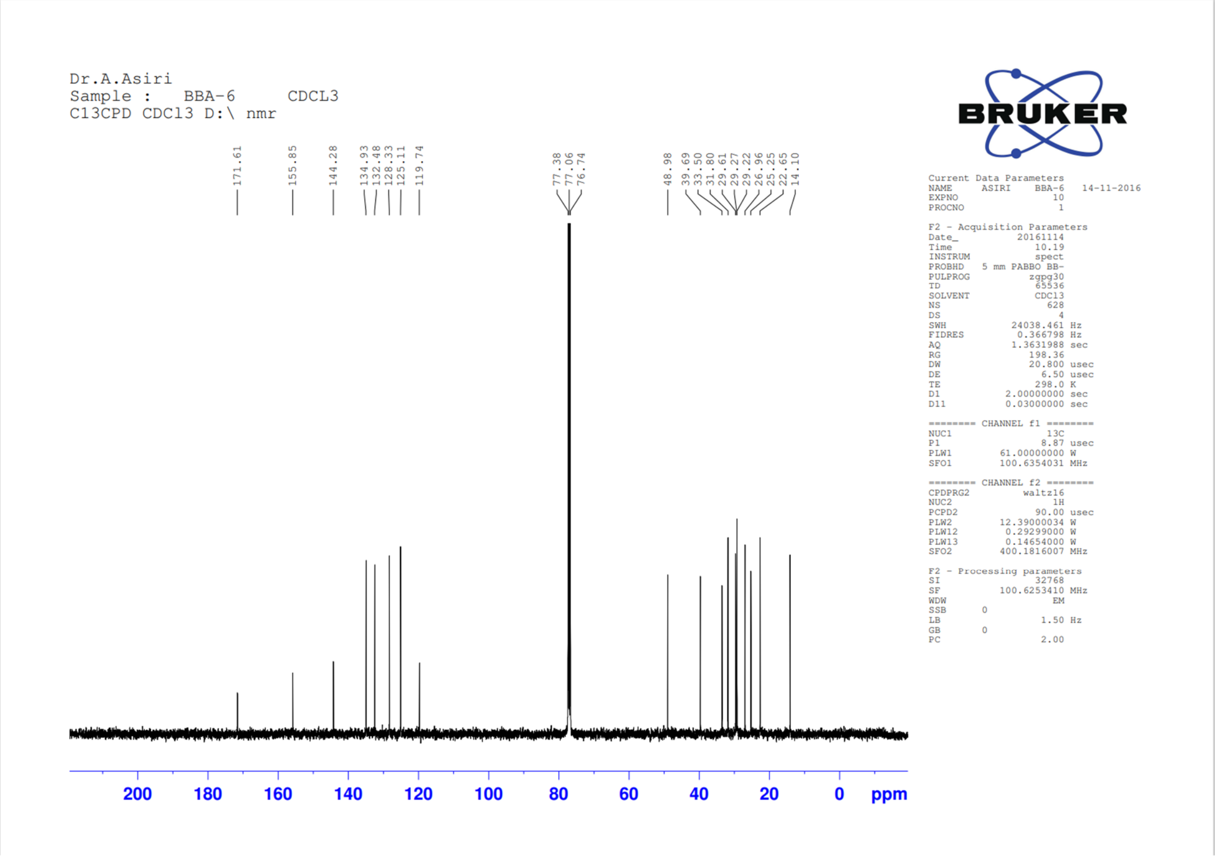

Figure S35. ^{13}C-NMR of 14f

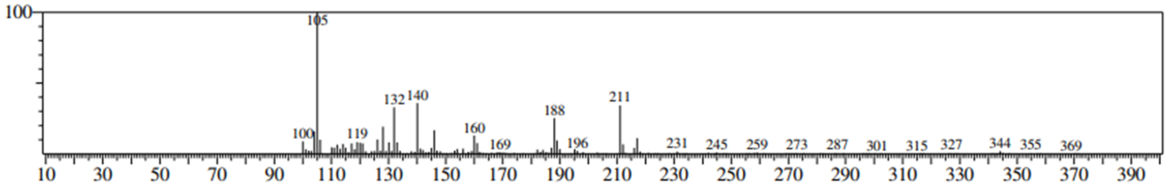

Figure S36. EIMS of 14f

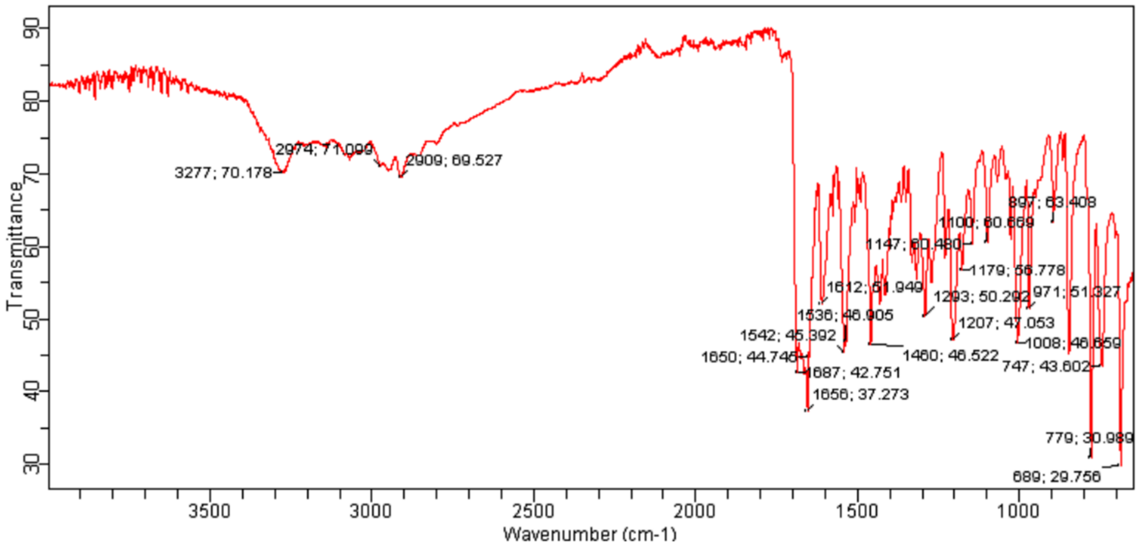

Figure S37. FT-IR of 14g

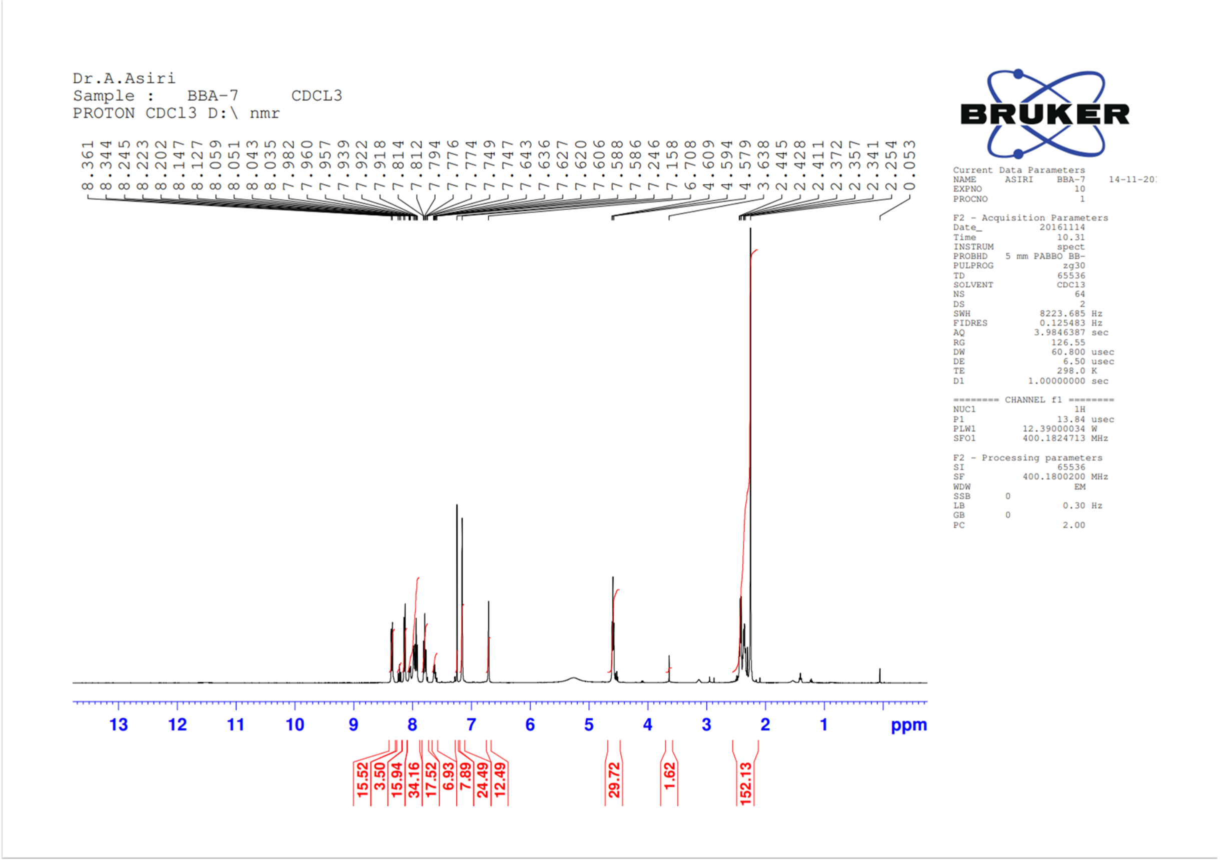

Figure S38. ^{1}H-NMR of 14g

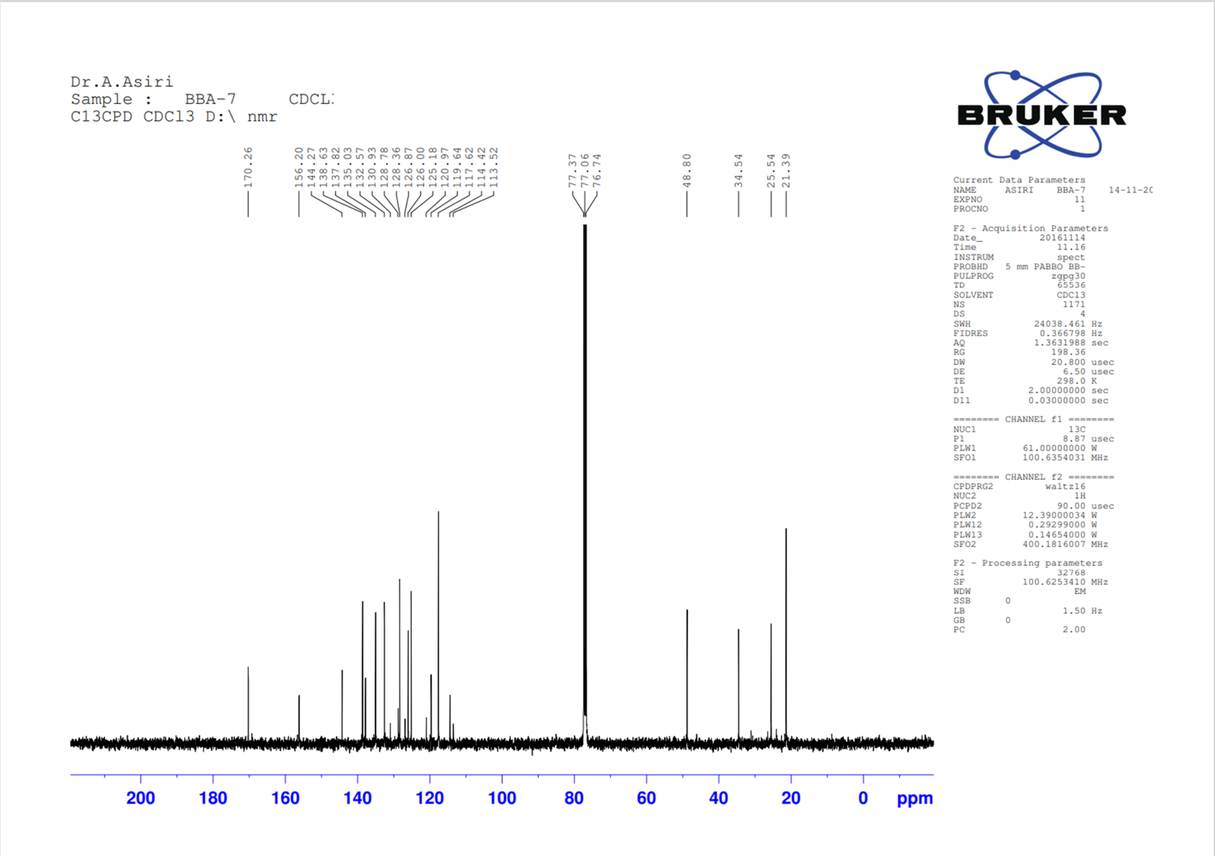

Figure S39. ^{13}C-NMR of 14g

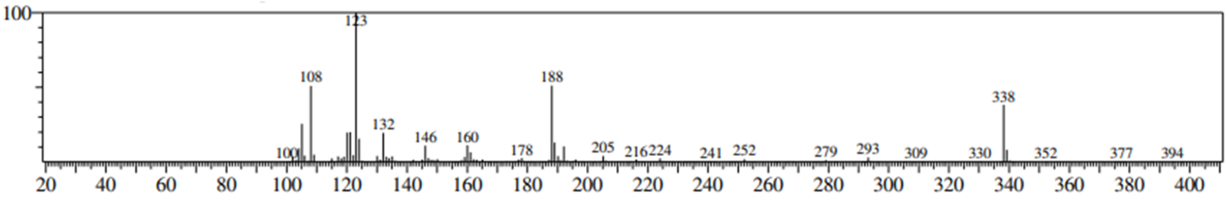

Figure S40. EIMS of 14g

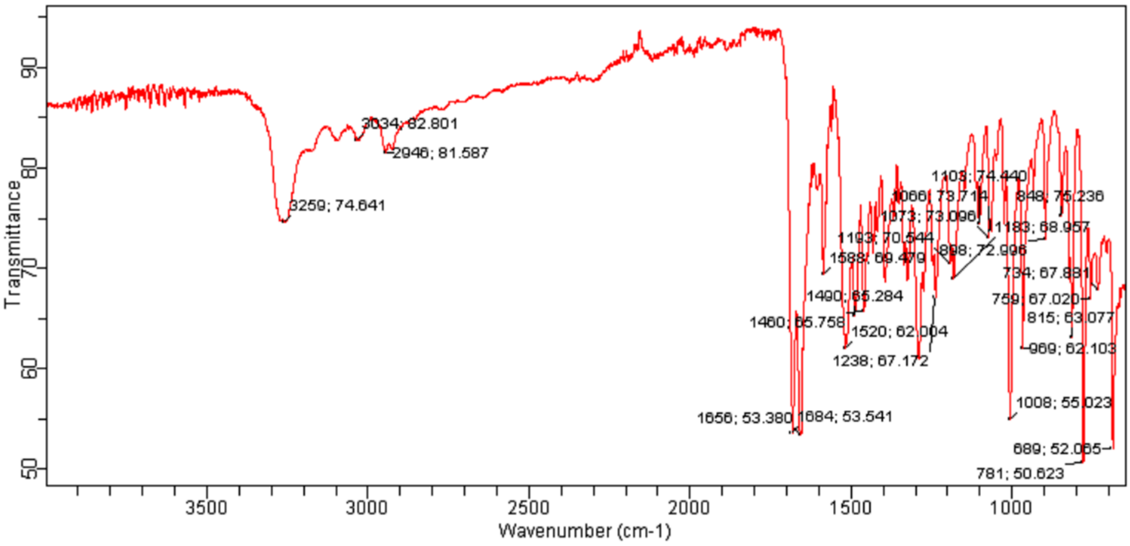

Figure S41. FT-IR of 14h

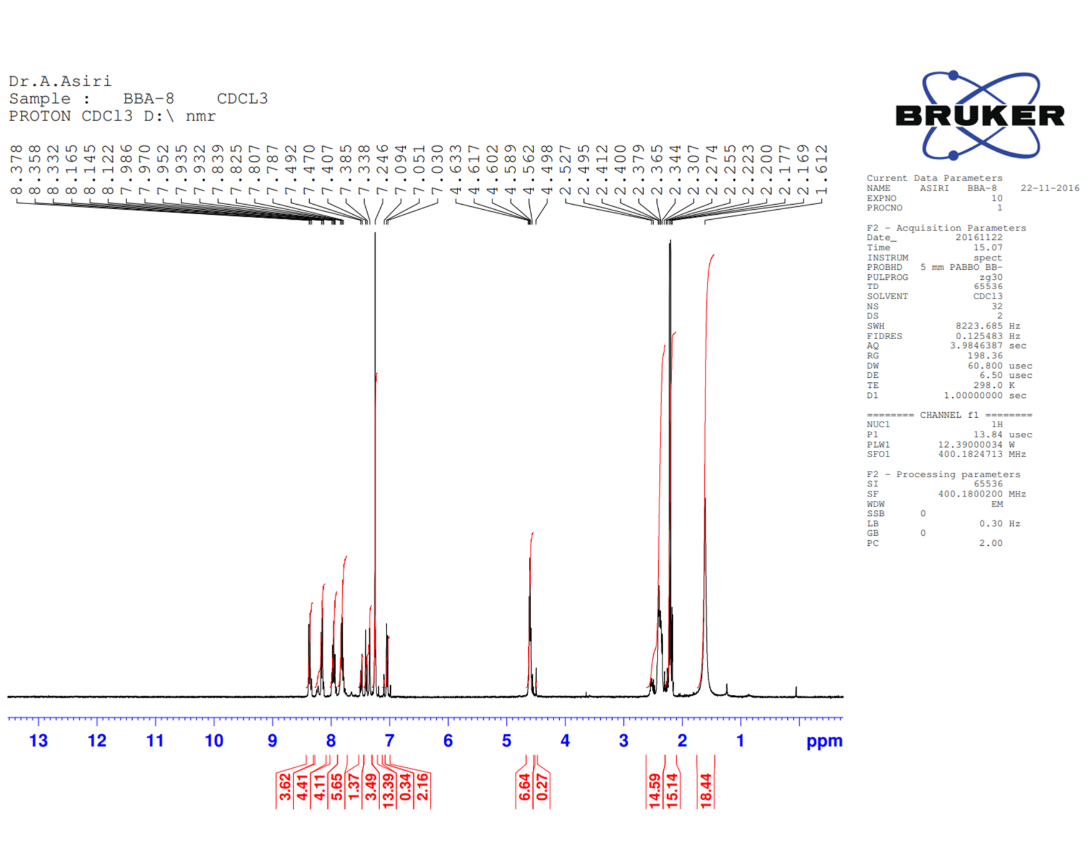

Figure S42. ^{1}H-NMR of 14h

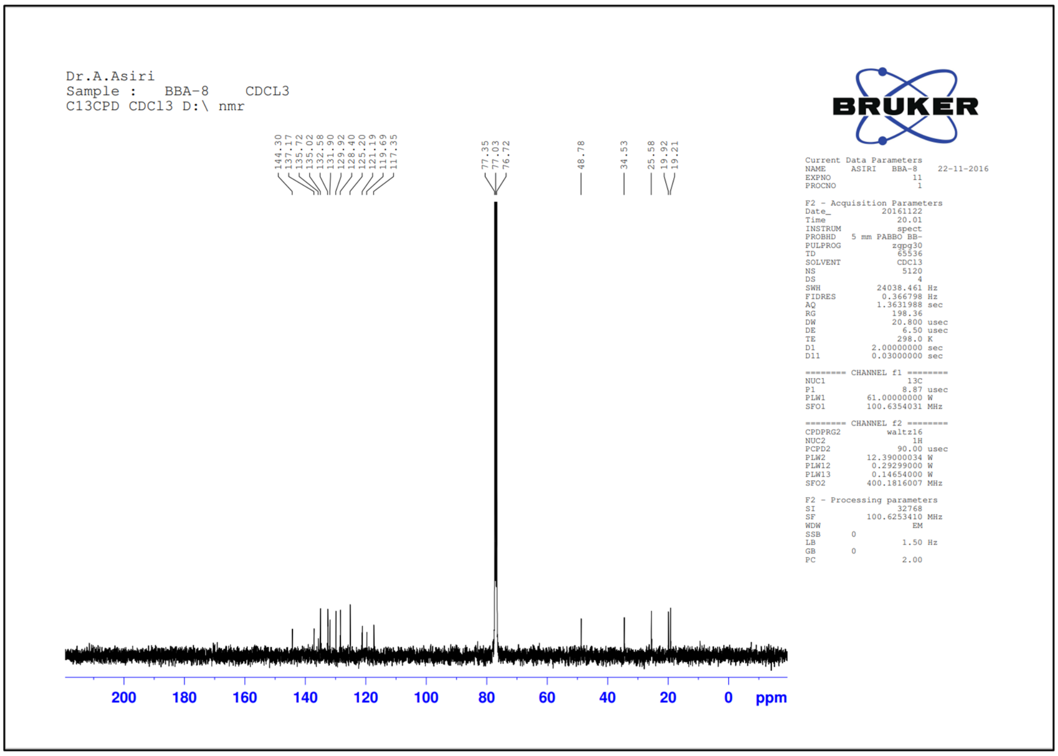

Figure S43. ^{13}**C-NMR of 14h**

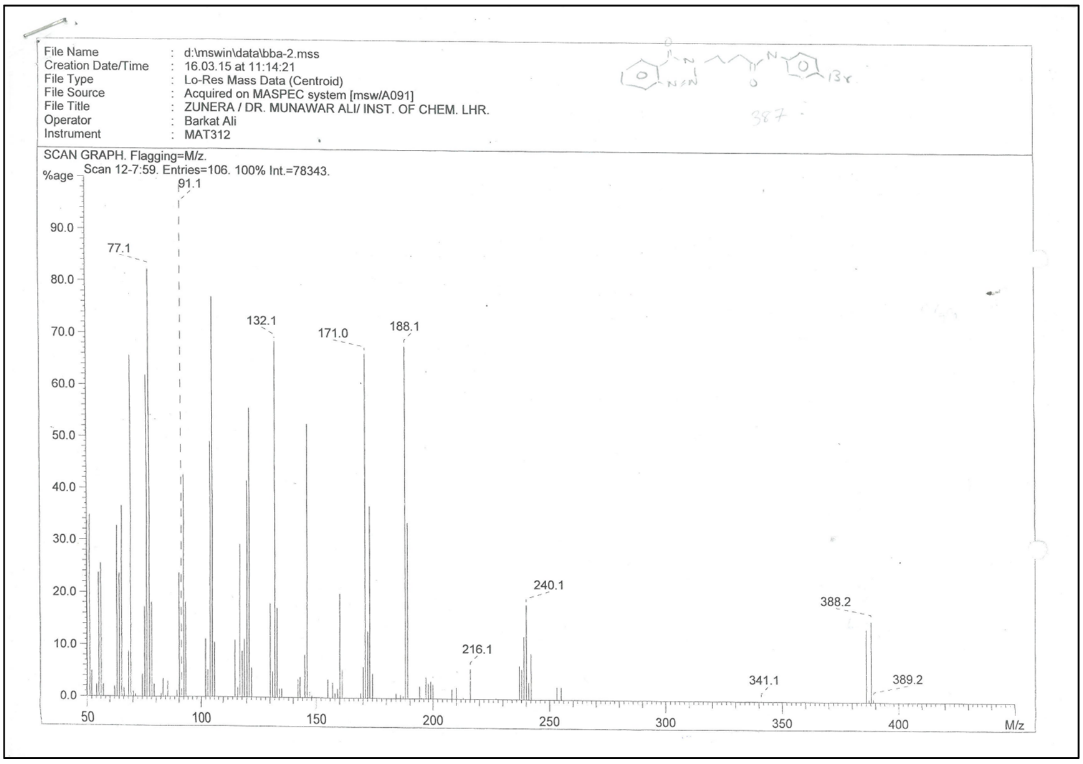

Figure S44. EIMS of 14h

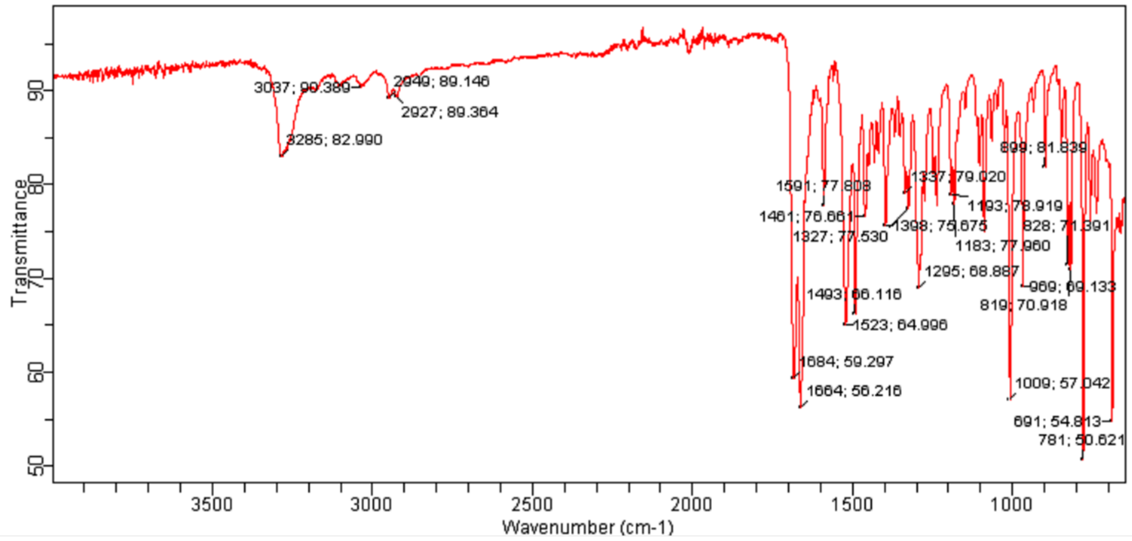

Figure S45. FT-IR of 14i

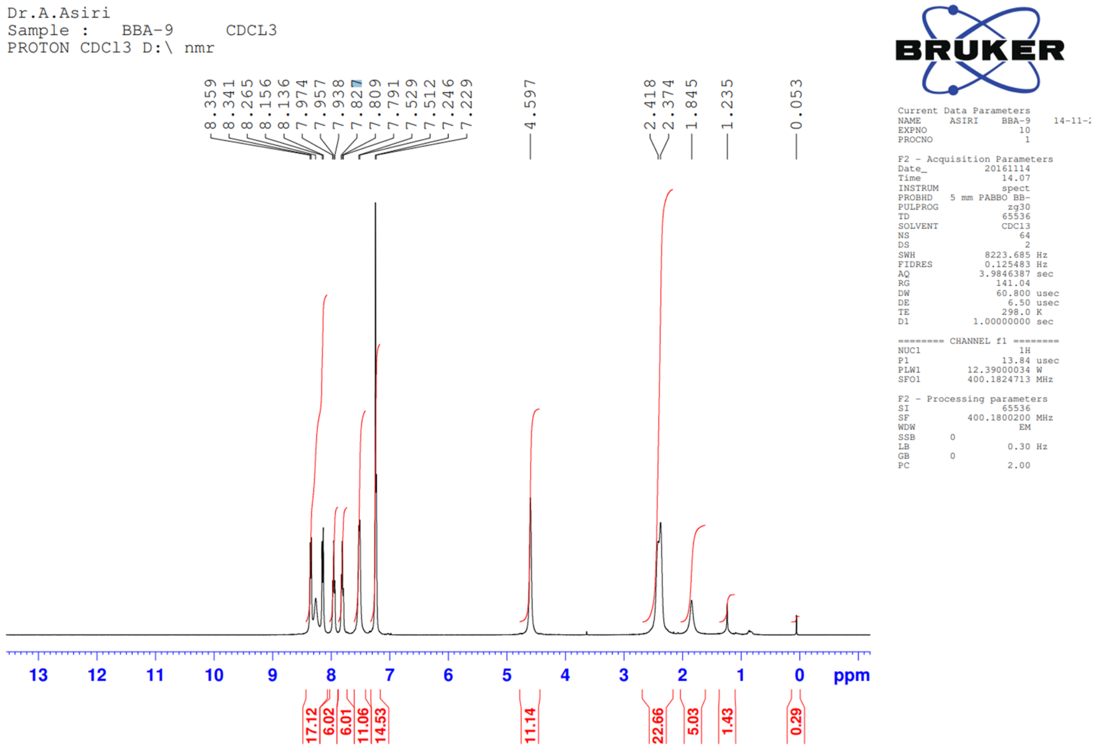

Figure S46. ^{1}H-NMR of 14i

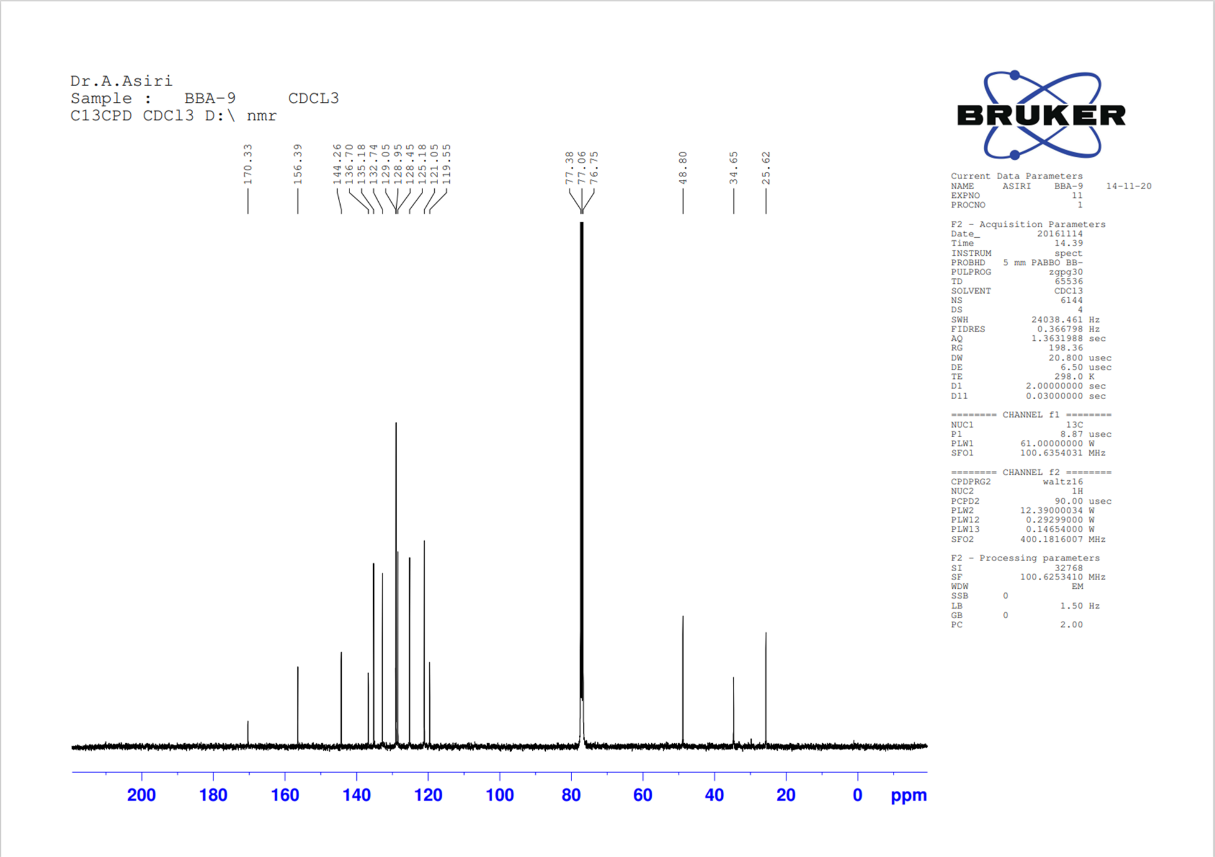

Figure S47. ^{13}**C-NMR of 14i**

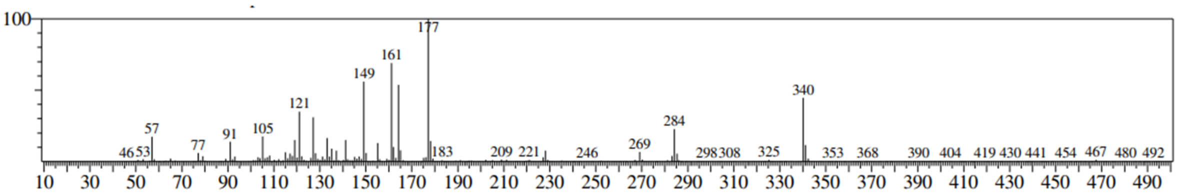

Figure S48. GC-MS of 14i

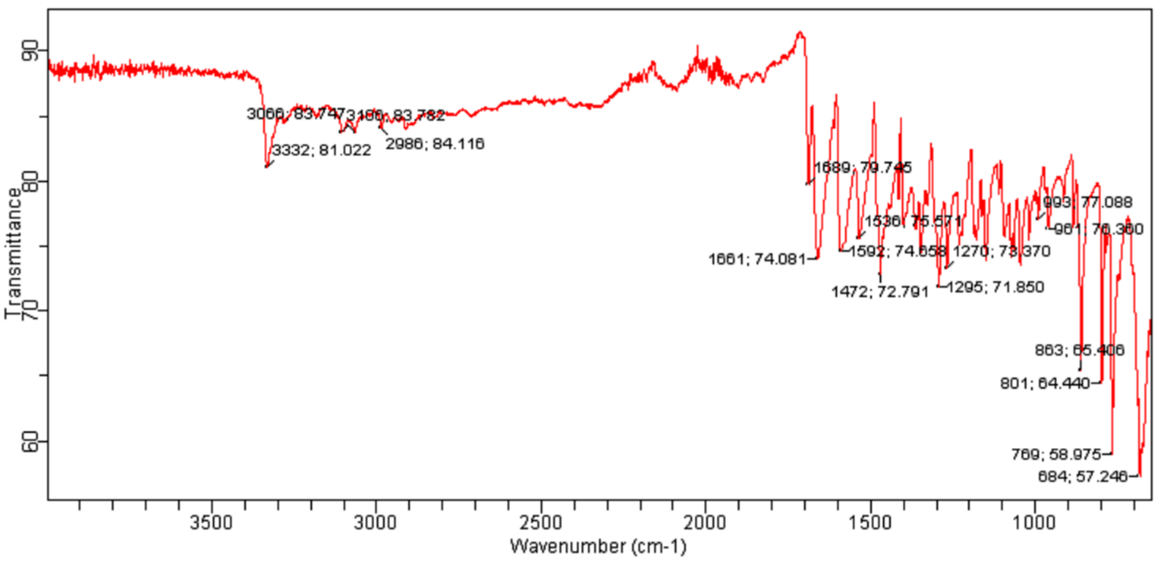

Figure S49. FT-IR of 14j

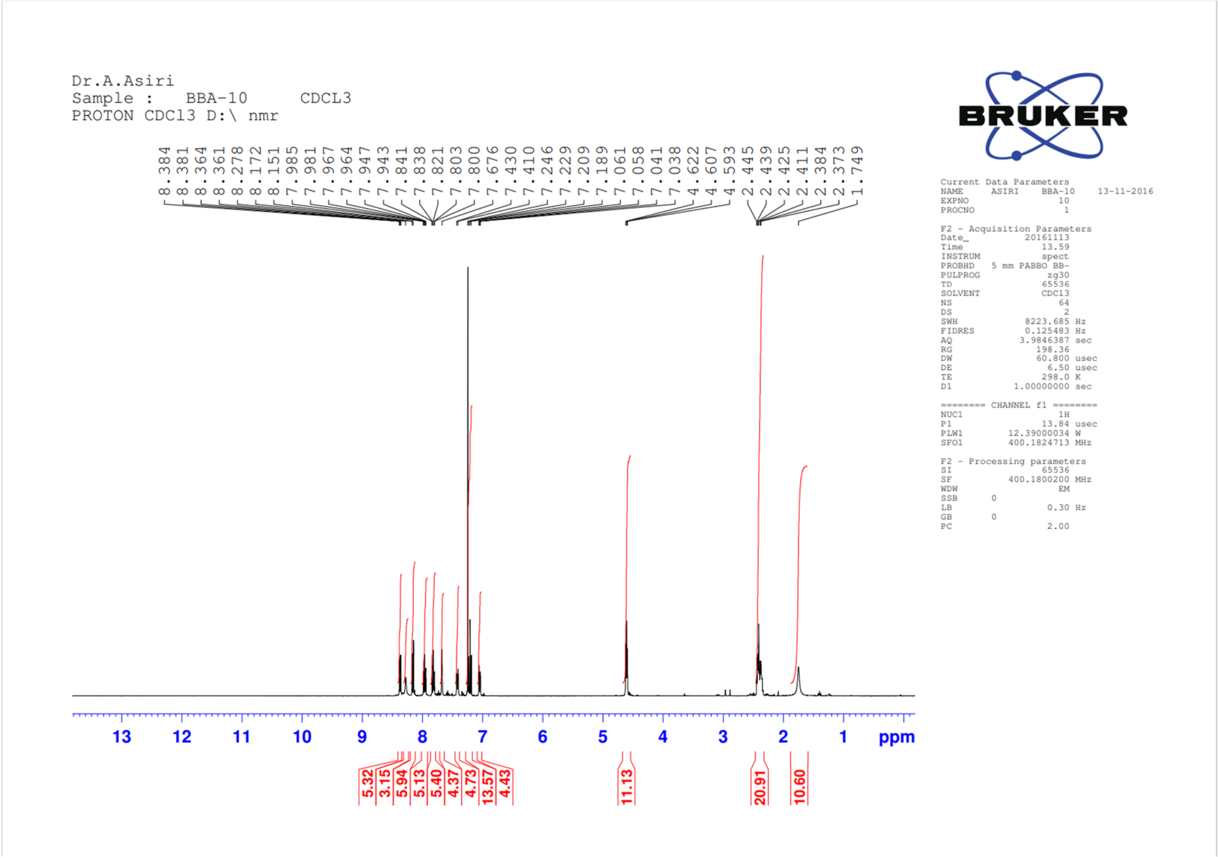

Figure S50. ^{1}H-NMR of 14j

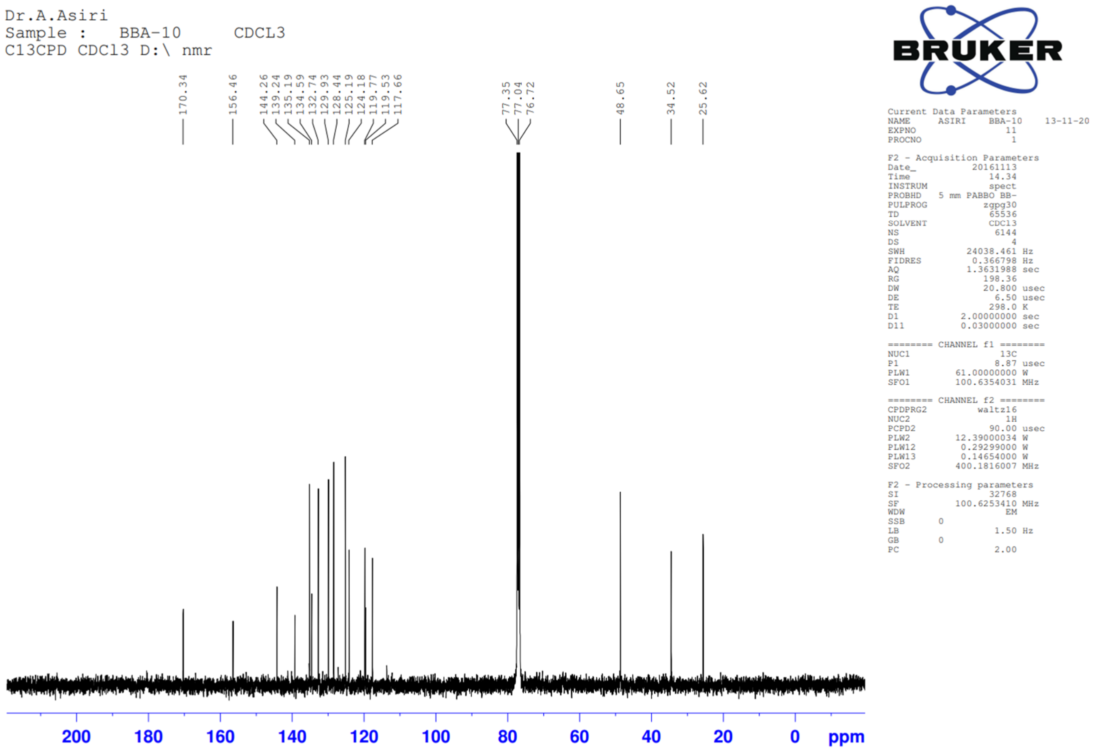

Figure S51. ^{13}C-NMR of 14j

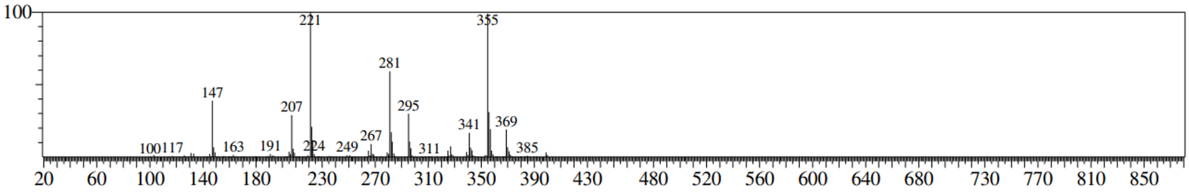

Figure S52. GC-MS of 14j

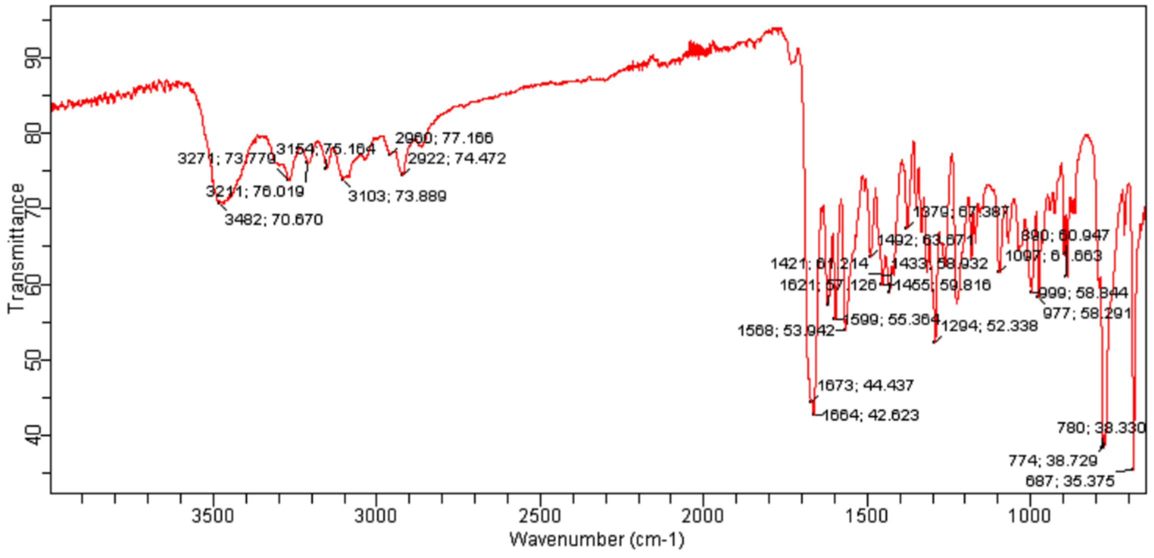

Figure S53. FT-IR of 14k

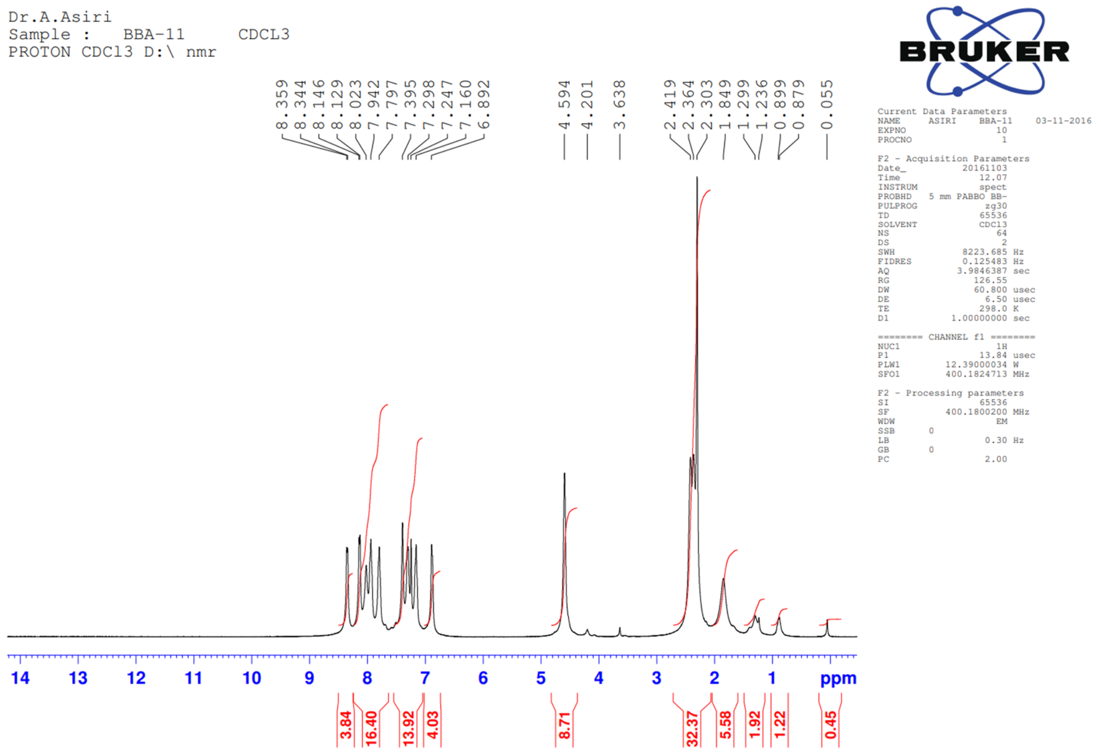

Figure S54. ^{1}H-NMR of 14k

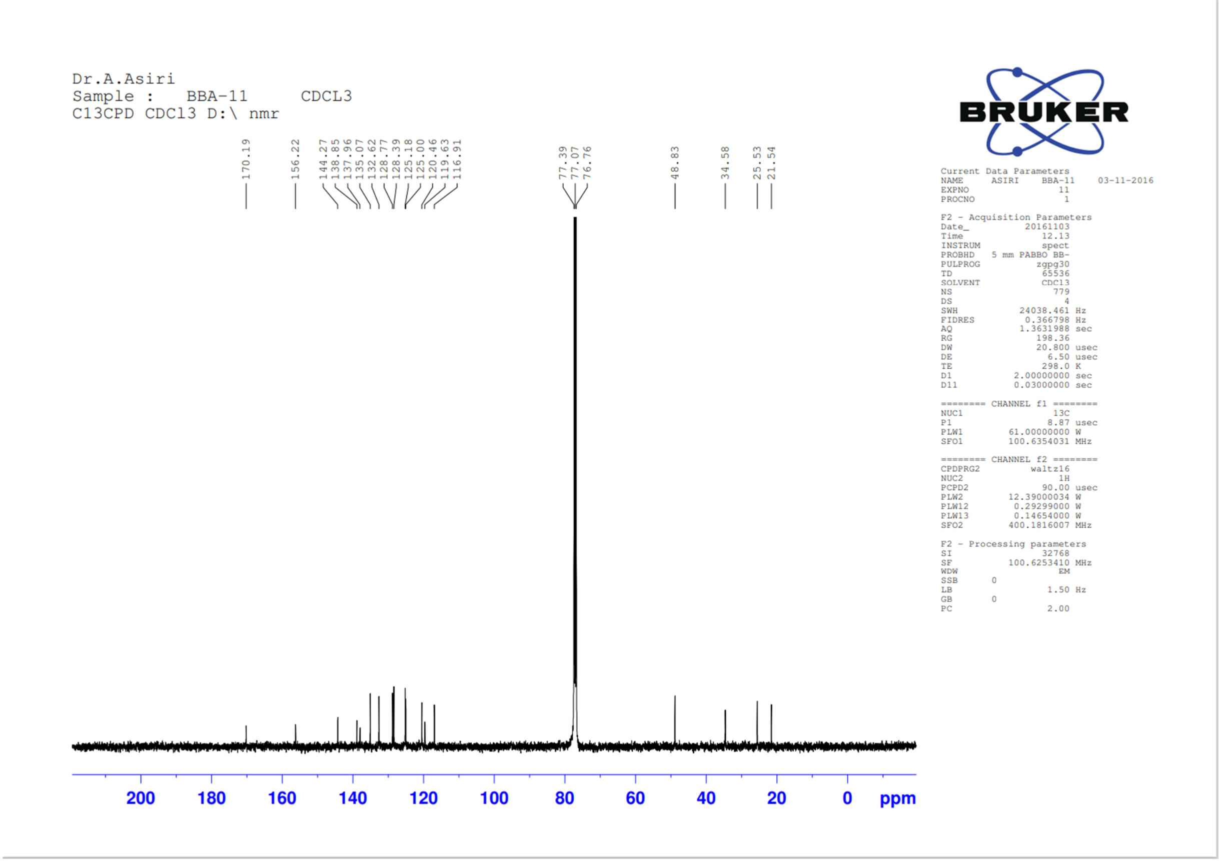

Figure S55. ^{13}**C-NMR of 14k**

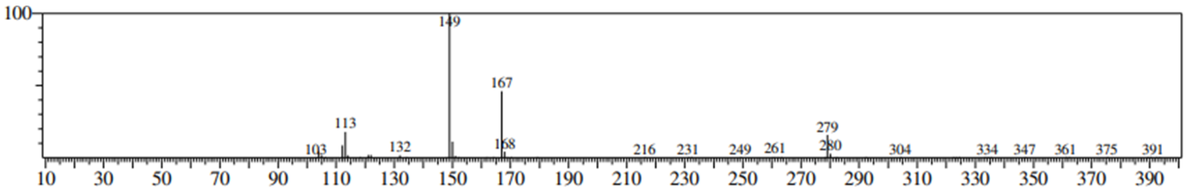

Figure S56. GC-MS of 14k

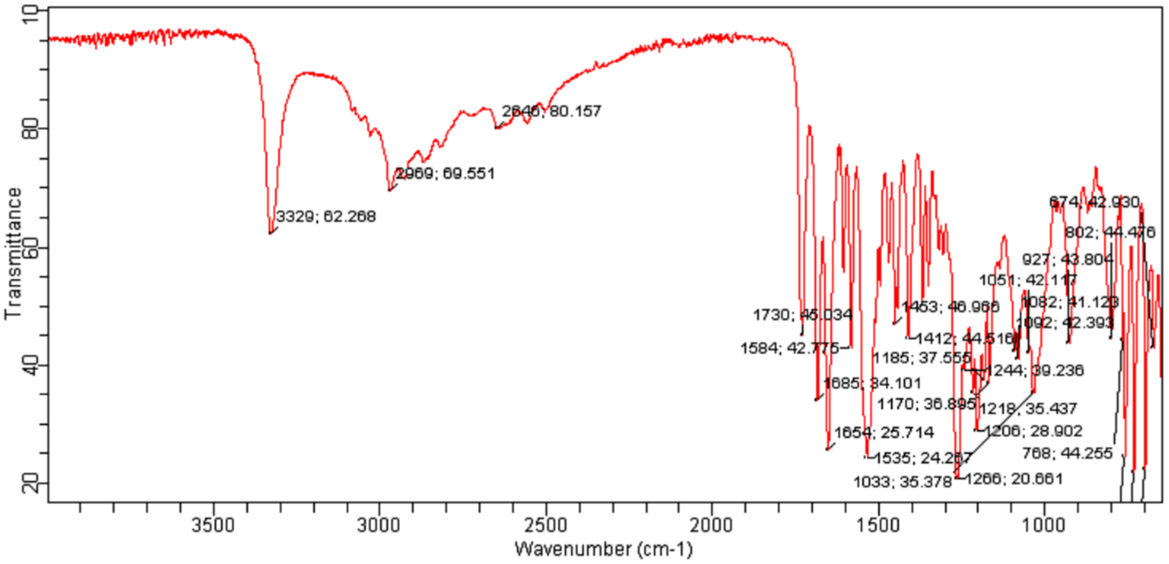

Figure S57. FT-IR of 14l

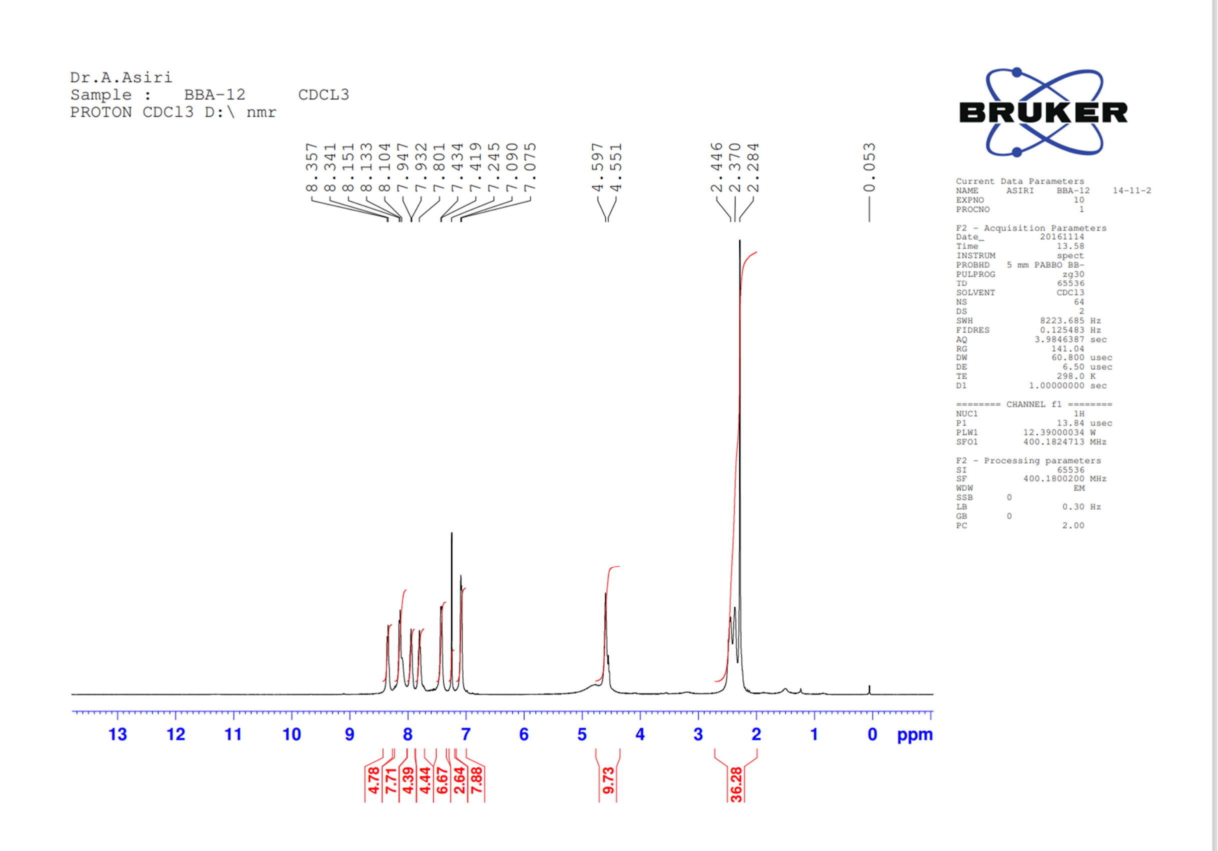

Figure S58. ^{1}H-NMR of 14l

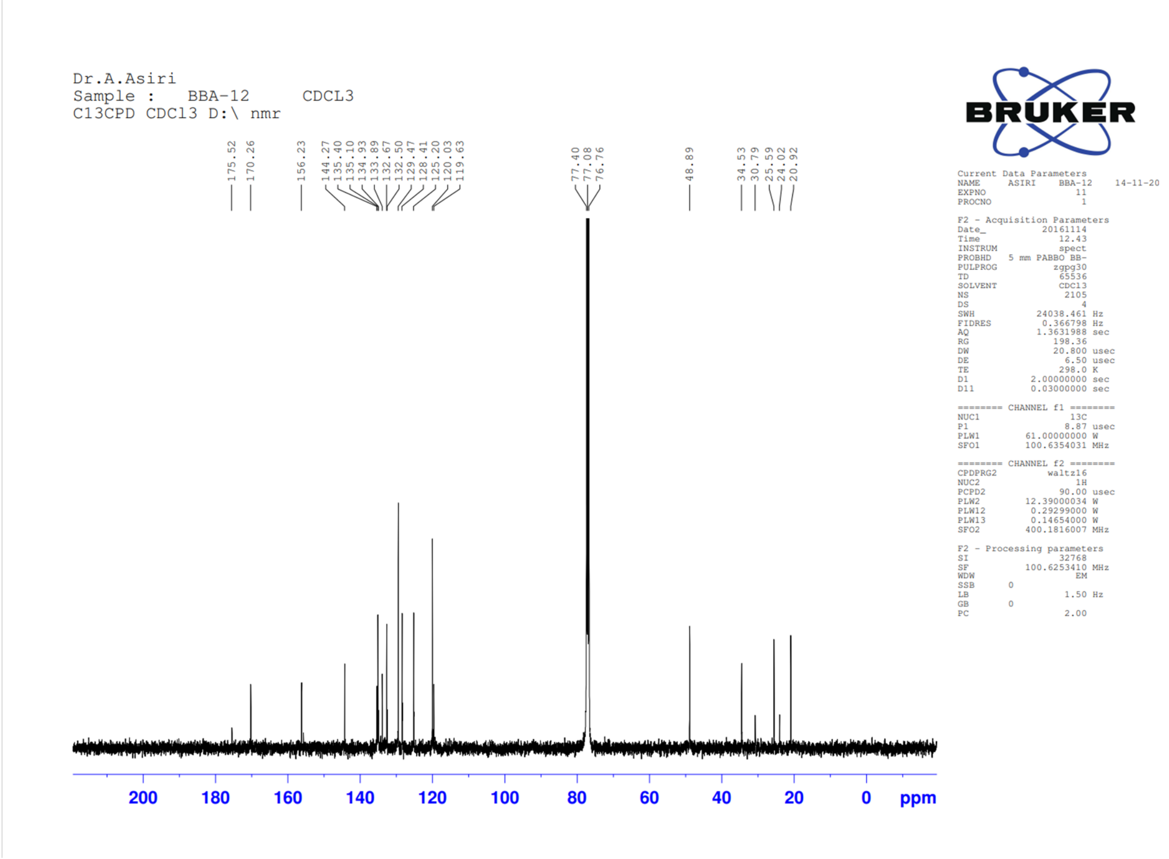

Figure S59. ^{13}C-NMR of 14l

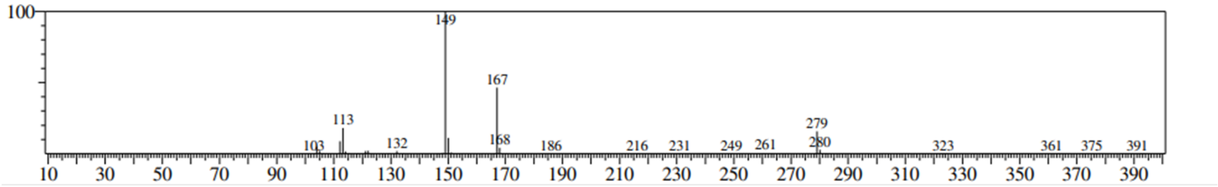

Figure S60. ^{13}C-NMR of 14l

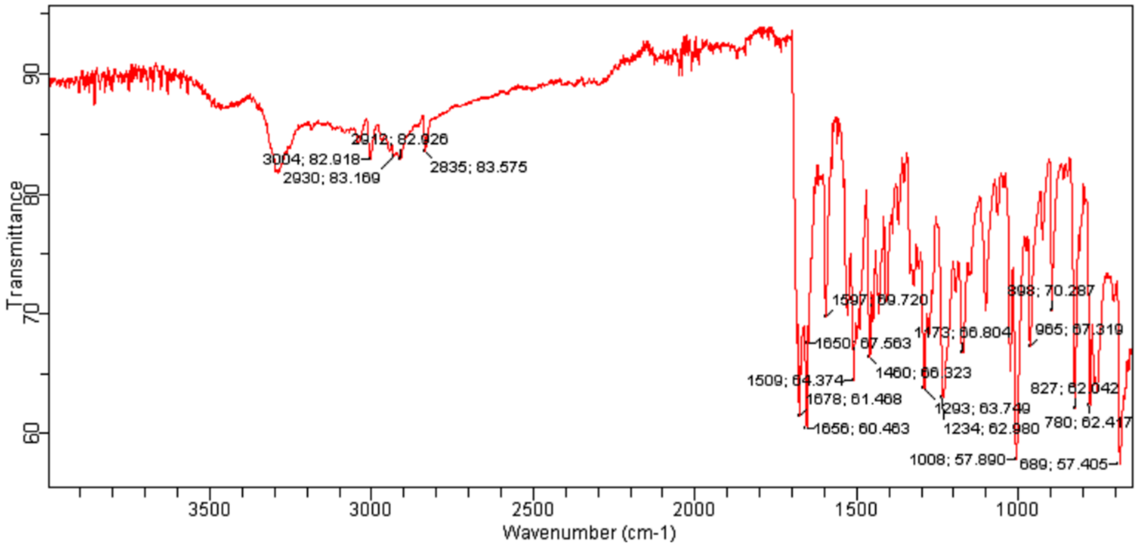

Figure S61. FT-IR of 14m

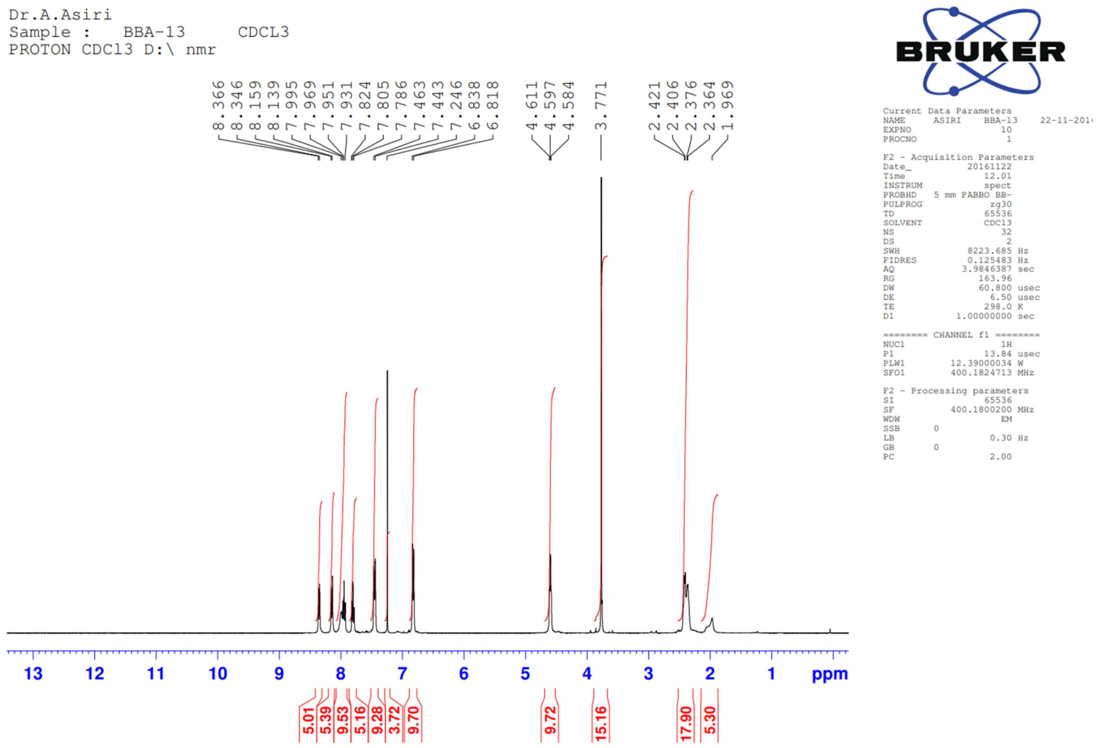

Figure S62. ^{1}H-NMR of 14m

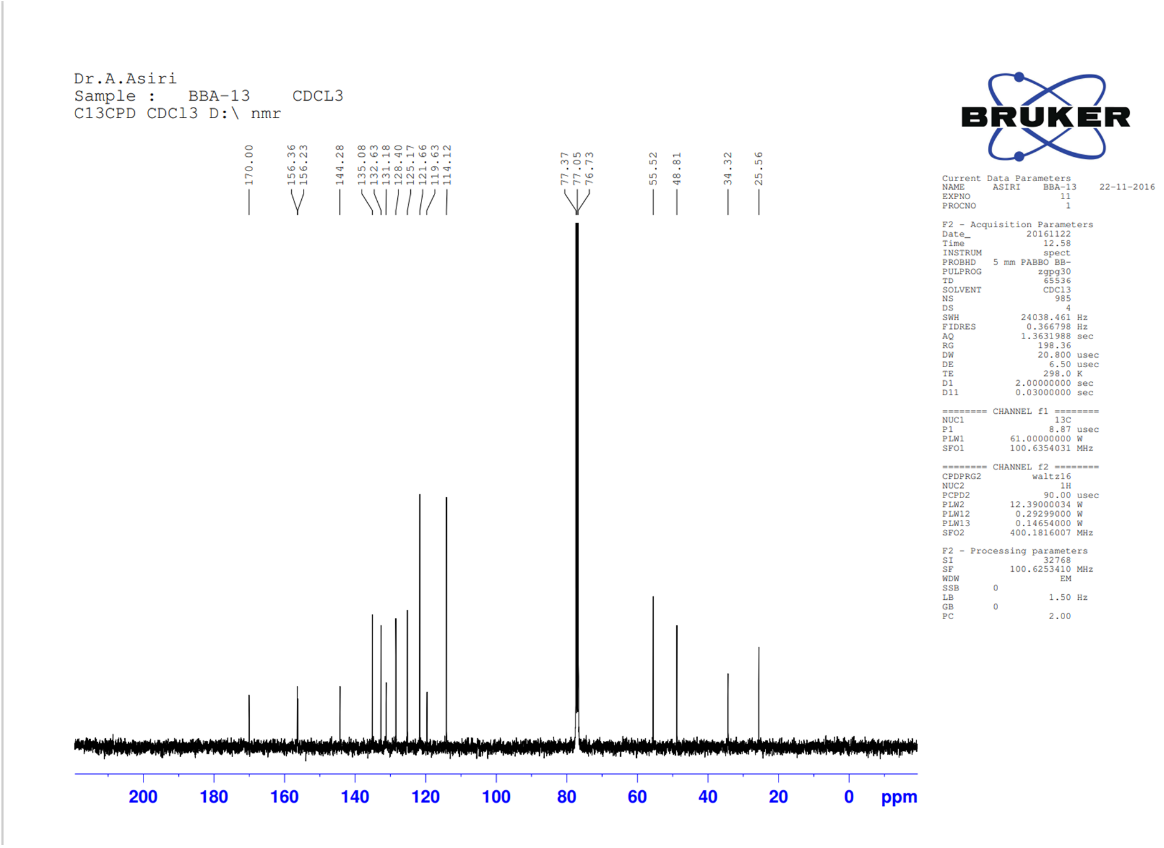

Figure S63. ^{13}**C-NMR of 14m**

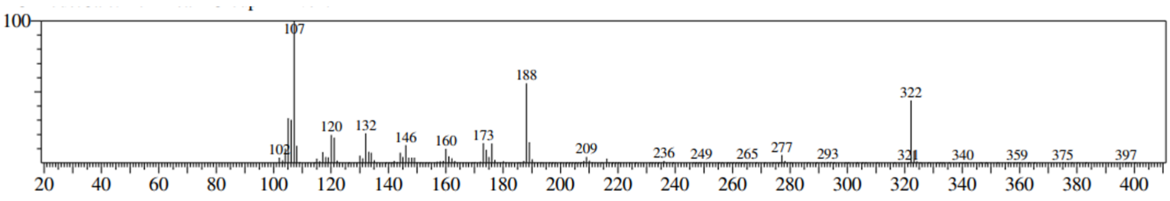

Figure S64. GC-MS of 14m

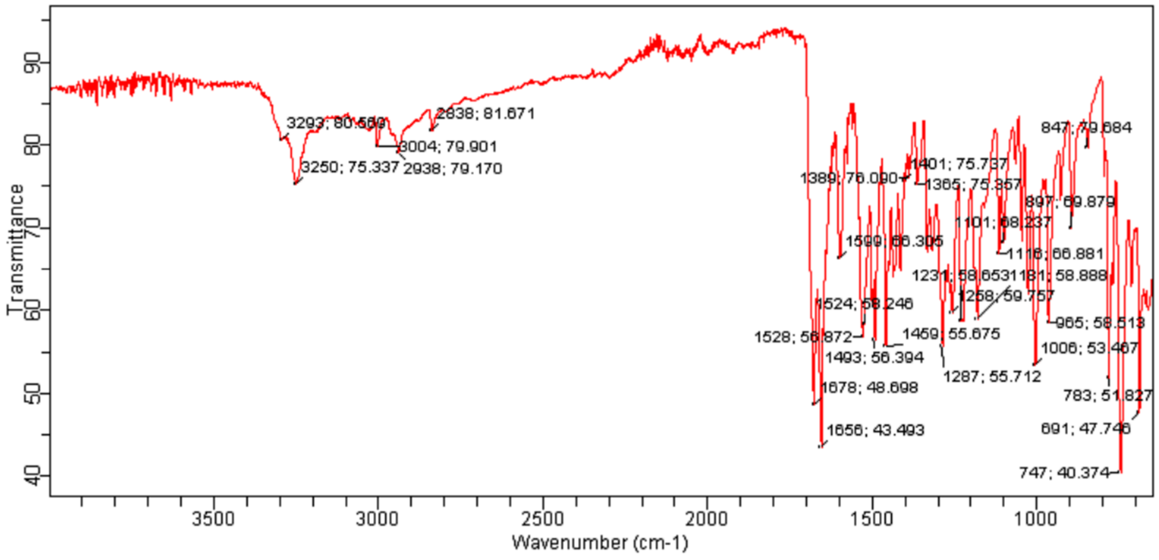

Figure S65. FT-IR of 14n

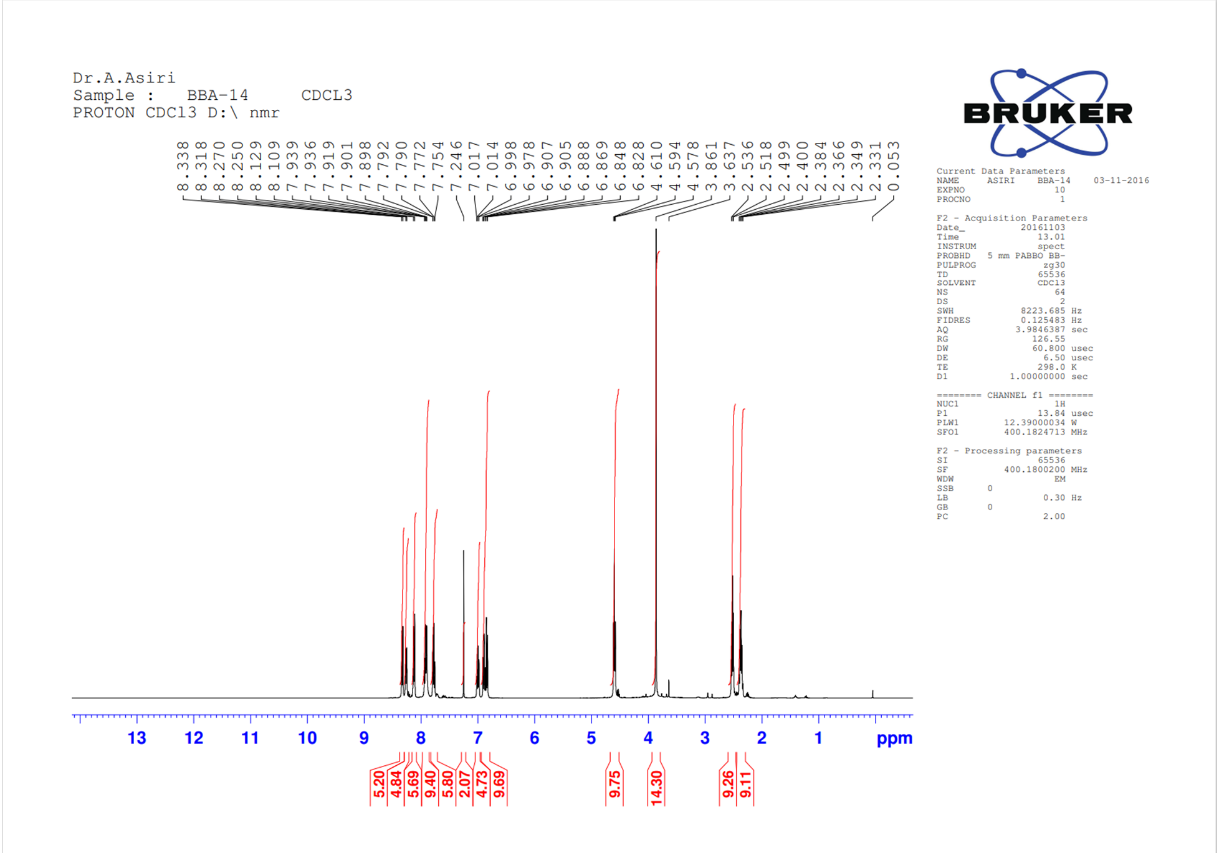

Figure S66. ^{1}H-NMR of 14n

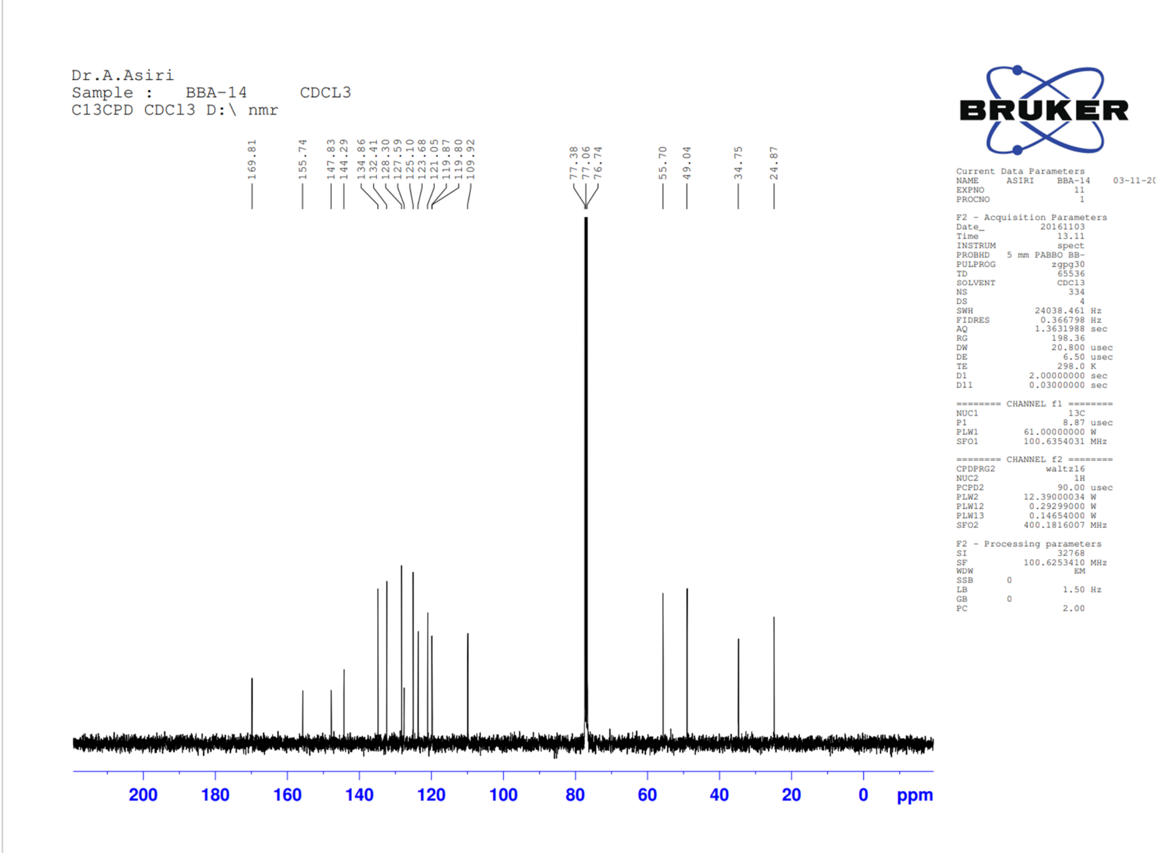

Figure S67. ^{13}C-NMR of 14n

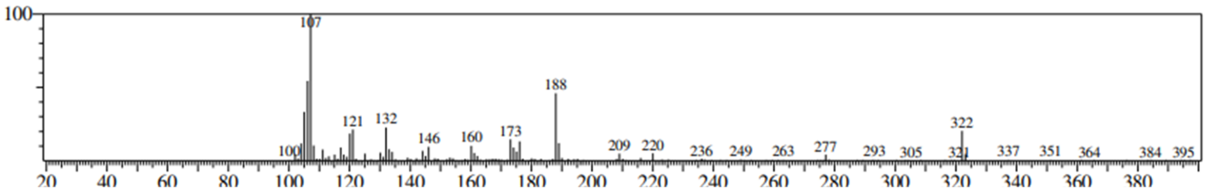

Figure S68. GC-MS of 14n
